# Elevated *Plasmodium falciparum* sexual conversion in HbAC and HbAS red blood cells in naturally infected malaria patients

**DOI:** 10.1371/journal.ppat.1014542

**Published:** 2026-09-29

**Authors:** Yasmina Drissi-El Boukili, Vera Kühne, H. Magloire Natama, Pieter Moris, Elisabet Tintó-Font, Antonia Windkouni Bere, Odin Goovaerts, Pieter Guetens, Ana Moreno-Murillo, Yarno Valgaerts, T. Edwig Traoré, D. Florence Ouedraogo, Halidou Tinto, Ann Van de Velde, Alfred Cortés, Guy Caljon, Anna Rosanas-Urgell

**Affiliations:** 1 Department of Biomedical Sciences, Institute of Tropical Medicine, Antwerp, Belgium; 2 Laboratory of Microbiology, Parasitology and Hygiene (LMPH), Infla-Med Centre of Excellence, University of Antwerp, Antwerp, Belgium; 3 Unité de Recherche Clinique de Nanoro, Institut de Recherche en Sciences de la Santé, Nanoro, Burkina Faso; 4 ISGlobal, Barcelona, Catalonia, Spain; 5 Laboratoire de Biochimie et d’Immunologie Appliquées (LaBIA), Ecole Doctorale Sciences et Technologies, Université Joseph Ki-Zerbo, Ouagadougou, Burkina Faso; 6 Department of Clinical Sciences, Institute of Tropical Medicine, Antwerp, Belgium; 7 Hematology and Hemostasis Unit, Universitair Ziekenhuis Antwerpen (UZA), Antwerp, Belgium; 8 ICREA, Barcelona, Catalonia, Spain; Kansas State University, UNITED STATES OF AMERICA

## Abstract

Malaria transmission relies on the differentiation of asexual parasites into gametocytes, a process initiated by sexual conversion (SC). Mutations in the host hemoglobin beta (HBB) gene are known to influence parasite growth and disease outcome, yet their impact on SC remains unclear. We investigated the effect of HBB mutant genotypes on *Plasmodium falciparum* (*P. falciparum*) SC and humoral immunity in individuals from Nanoro, Burkina Faso. To measure SC rates in natural human malaria infections, we developed a new *ex vivo* SC assay (*ev*SCA). We found that in human natural *P. falciparum* infections, SC rates were higher in individuals with HbAS or HbAC than in individuals with wild-type HBB (HbAA). Consistently, using an *in vitro* SC assay (*iv*SCA) based on the NF54-*gexp02-Tom* reporter line we found that cultures grown in HbAS red blood cells (RBCs) had higher SC rates than those grown in HbAA RBCs. Furthermore, IgG and IgM responses against trophozoite- and stage I gametocyte-infected RBC antigens, quantified by flow cytometry, did not differ between plasma from individuals with different HBB genotypes. These results demonstrate that exposure to RBCs with HBB mutations enhances SC, highlighting a host-genetic factor that may influence malaria transmission potential.

## Author summary

Transmission of *Plasmodium falciparum,* a malaria causing parasite, depends on the parasite’s ability to produce gametocytes, the stage that infects mosquitoes. In many African regions where malaria is common, hemoglobin (Hb) variants such as hemoglobin S (the sickle-cell trait) and C are highly prevalent reflecting their role in protecting against severe disease. However, it remains unclear whether these variants also influence the parasite’s early shift toward gametocyte development (*i.e.,* sexual conversion [SC]). In this study, we combined *ex vivo* and *in vitro* SC assays to measure SC in red blood cells with different hemoglobin genotypes. We found that SC was higher in parasites growing in red blood cells carrying hemoglobin AC and AS genotypes compared to normal AA hemoglobin. These findings provide experimental support for earlier epidemiological observations reporting that individuals with these hemoglobin variants often carry more gametocytes and are more infectious to mosquitoes. We also measured antibody levels in individuals with mutant RBCs and found no differences in immunity that could explain the variation in SC. Our results show that host genetic background can influence gametocyte development and may shape the infectious reservoir in high-burden settings. This knowledge is important for designing effective strategies for malaria elimination.

## Introduction

*Plasmodium falciparum* (*P. falciparum*) causes over half a million deaths annually, mostly in young children from low- and middle-income countries. Despite the substantial progress in reducing the global malaria burden during the last decade, this decline has stalled, with some countries reporting a resurgence in clinical cases [1]. Innovative approaches for malaria control and elimination are thus urgently needed.

Plasmodium infections have exerted strong evolutionary pressure on the human genome, with hemoglobin mutations providing a well-documented example [2–5]. Mutations in the human hemoglobin beta (HBB) gene, such as hemoglobin S (HbS) and hemoglobin C (HbC), result from a substitution of glutamic acid at position 6 with valine in HbS and lysine in HbC. Individuals can carry homozygous (HbSS or HbCC), heterozygous (HbAS or HbAC) or compound heterozygous (HbSC) mutations [6–8]. HBB mutations lead to significant changes in red blood cell (RBC) morphology and function, influencing malaria susceptibility and clinical outcomes [5,9,10]. HbS mutations induce hemoglobin polymerization under low oxygen, causing RBCs to reshape into sickle forms. HbSS causes ~60–80% RBC sickling [2] with severe complications including vaso-occlusion, hemolysis, and chronic anemia [11,12]. This results in early-life mortality, with estimates ranging from 50-90% among children in low-and middle income countries [13]. On the other hand, HbAS carriers are typically asymptomatic [2], with fewer sickled RBCs (~40%) [12,14]. HbC mutations cause hemoglobin crystallization without sickling but increase blood viscosity and shorten RBC life span [15]. HbCC and HbAC are usually a mild and asymptomatic disorder, with HbCC occasionally causing mild chronic hemolytic anemia in some individuals [2,7,15]. HbSC carriers display moderate disease severity and a mixed phenotype with ~50% sickled/crystallized and “pita-shaped” RBCs [2,11,16].

HbSS is relatively rare (~2%), likely due to its high mortality, whereas HbCC, although a mild disorder, is also relatively uncommon (~2%). HbAS and HbAC can reach frequencies of 10–30% in regions such as Mali and Burkina Faso, which has been linked to the protection they confer against *P. falciparum* malaria [4,5,8,17–20]. HbAC offers mild protection against severe malaria, whereas HbAS and HbCC provide strong protection against both uncomplicated and severe malaria [5,15,21–23]. How different HBB mutations protect against malaria is not fully understood, but several mechanisms have been proposed [4,24–31]. HbS infected-RBCs (iRBCs), especially in HbSS, exhibit elevated heme and HO-1 − mediated oxidative stress. This causes polymerization, disrupts the actin cytoskeleton, alters iRBCs morphology, and impairs the surface expression of *P. falciparum* erythrocyte membrane protein 1 (PfEMP1) on iRBCs [32–34]. In HbAS iRBCs, this reduces cytoadherence and sequestration, promoting splenic clearance and controlled parasite infection [25,35].

Beyond protection from disease, HbS and HbC RBCs may influence the transmission potential of *P. falciparum*. Population-based studies have reported conflicting evidence regarding whether HbAS influences gametocyte carriage. Some studies reported no significant association between HbAS and increased gametocyte carriage, while HbAC and HbCC were associated with increased gametocyte carriage in the same [36] and other populations [37]. In contrast, other studies found a link between HbAS and gametocyte carriage [38–40]. Some studies have also reported higher infectivity to mosquitoes in individuals carrying HbAS [36,37,40], HbAC [36,37] and HbCC [36] compared to wild-type HbAA iRBCs.

Gametocyte production is initiated by a process called sexual conversion (SC), in which a variable but typically small proportion (~1%) of asexual *P. falciparum* parasites commit to sexual development at each replication cycle, a proportion referred to as the SC rate (SCR), and initiate gametocytogenesis [41–43]. Sexual commitment is marked by epigenetic activation of the master regulator *pfap2g*, a transcription factor that irreversibly results in SC and gametocyte development [44–47]. Sexual rings circulate in blood [48–51], while maturation of gametocyte stages I to IV occurs mainly in the bone marrow over ~10 days [52]. Mature male and female stage V gametocytes re-enter the peripheral blood, resulting in gametocyte carriage, and are the only parasite stages capable of infecting *Anopheles* mosquitoes. Gametocyte carriage determines human to mosquito transmission and correlates with infectivity to mosquitoes [53–58].

The elevated gametocyte carriage reported in individuals with HbAC [36,37] and, in some studies, HbAS genotypes [38–40] could result from elevated SC but also from prolonged infections, leading to accumulation of gametocytes in peripheral blood over numerous replication cycles. Several host related factors have been shown to increase SC [59], including lysophosphatidylcholine depletion [60–62], reticulocyte-rich environments [63,64], and antimalarial treatment [65–68]. However, other studies did not find enhanced SC after antimalarial treatment [67]. To date, the link between SC and HBB genetics has been explored to a limited extent, with one study reporting that increased intracellular heme in HbS iRBCs promotes SC [32]. Another potential driver of elevated SC in HBB mutant RBCs is the host immunity against *P. falciparum*. HbS and HbC mutations appear to modulate host immunity against *P. falciparum* by increasing regulatory cytokines (*e.g.,* IL-10) [30], upregulating HO-1 [28], and dampening pro-inflammatory responses (*e.g.,* TNF-α, IFN-γ) [24], which both protect against severe malaria and enhance parasite clearance. While there is evidence of a differential humoral immune response between different HBB genotypes, differences were only detectable in certain populations and for very specific antigens [69–73].

Discrepancies in gametocyte carriage studies highlight the need to investigate upstream processes such as SC. Determining if HBB mutant RBCs enhance *P. falciparum* SC could confirm that carriers of these mutant RBCs are key contributors to the infectious reservoir – as proposed by mosquito feeding studies [36,37,40], informing strategies to reduce transmission.

In this study, we investigated the impact of host HBB mutant genotypes (HbAA, HbAC, HbAS, HbSS, HbCC and HbSC) on SC in naturally infected malaria patients and *in vitro P. falciparum* cultures, and on IgG and IgM reactivity against trophozoite- and stage I gametocyte-iRBCs by flow cytometry.

## Results

### HbAC and HbAS genotype prevalences in Nanoro, Burkina Faso

Using Sanger Sequencing, the HBB gene was genotyped in participants of a longitudinal cohort study (*i.e., InHost* study [74]) in Nanoro, Burkina Faso (*n* = 864) and the population frequencies of HbAA, HbAC, HbCC, HbAS, HbSS, and HbSC genotypes were determined. HbAA (wild-type) was the most frequent (*n* = 618; 71.5%), followed by HbAC (*n* = 173; 20%), HbAS (*n* = 64; 7.5%), HbSC (*n* = 7; 0.8%), and HbCC (*n* = 2; 0.2%). No HbSS individuals were identified.

### Novel *ex vivo* assay reveals elevated sexual conversion in HbAS/HbAC

To investigate the effect of HBB genotypes on *P. falciparum* SC we measured SCRs *in vivo* in *P. falciparum* isolates collected from naturally infected individuals with different HBB genotypes (14 HbAA, 12 HbAC, and 6 HbAS) living in the malaria-endemic area of Nanoro, Burkina Faso (Fig 1). For this, we developed a new *ex vivo* SC assay (*ev*SCA), optimized and validated as described in S1 Appendix. Sample selection criteria are described in S1 Fig. Specifically, only samples with a minimal detectable parasitemia of 0.05% were included in the *ev*SCAs. To assess the proportion of circulating rings in infected individuals that are sexual or asexual, parasites were cultured *ex vivo* in the presence of the cGMP-dependent protein kinase (PKG) inhibitor ML10 (to prevent reinvasion [75]) until the majority of parasites reached the schizont stage. At this time, sexual rings have progressed to Pfs16-positive stage I gametocytes. Next, parasites were concentrated using magnetic columns (MACS, magnetic activated cell separation), and thin blood smears were prepared from the enriched fractions. SCRs were quantified by immunofluorescent staining (IFAs) detecting the proportion of parasites that stained positive for Pfs16 (stage I gametocytes) [76–78] relative to the total number of parasites, stained for DNA/parasite nuclei (the total parasite population consisting of stage I gametocytes plus schizont stages) [45,79,80]. The experimental set-up of the *ev*SCAs and representative IFA images are shown in Fig 2A and 2B, respectively. The median SCR was 2.65% (IQR 1.72) in *P. falciparum* isolates from HbAA genotype, 5.05% (IQR 2.55) for HbAC and 18.40% (IQR 19.70) for HbAS genotypes. SCR increased modestly in HbAC (beta-binomial generalized linear mixed model [GLMM], odds ratio (OR)[95%CI] = 1.82 [1.17 − 2.82], *p (Bonferroni)* = 0.0032) and strongly in HbAS (beta-binomial GLMM, OR [95%CI] = 8.16 [4.34 − 15.37], *p (Bonferroni)* < 0.0001) genotypes compared to HbAA (Fig 1C, S1A Table in S1 Table). The fold-change between median SCR of *P. falciparum* isolates in HbAC and HbAS relative to the median SCR of wild-type HbAA RBCs was 2.04 (IQR 0.85) in HbAC and 7.07 (IQR 7.32) in HbAS, reflecting the increased SCRs in HbAC and HbAS RBCs compared to HbAA.

**Fig 1:**
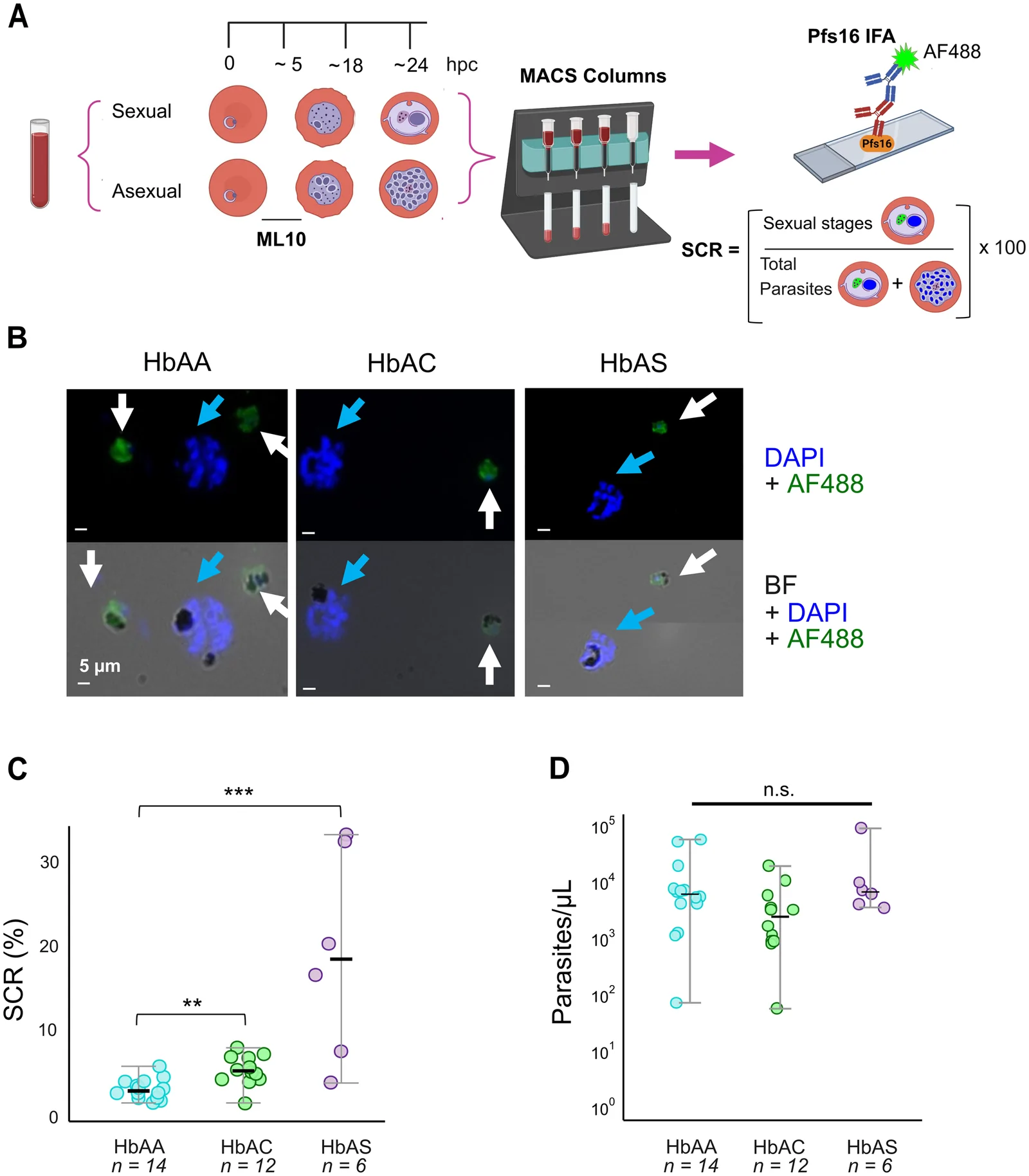
Effect of HbAA, HbAC and HbAS on SC determined with *ev*SCAs. **(A)** Schematic representation of the *ex vivo* SC assays (*ev*SCAs). Parasite isolates from RBCs with different HBB genotypes cultured *ex vivo*, ML10 added at 11 hpi., MACS-enriched stage I gametocytes and schizonts detected by IFA Pfs16 (sexual stages) and DAPI (total parasites) staining. SCR (%): number of sexual stages/number of total parasites. **(B)** Representative IFA images of parasites in HbAA, HbAC and HbAS RBCs. Top row with DAPI (all parasites; blue arrows: schizonts) and AF488 (white arrows: stage I gametocytes) overlay and bottom row including brightfield (BF) channel to visualize the parasite’s hemozoin. Objective: 40 × ; Scale bar: 5µm. **(C)** SCRs by HBB genotype determined using IFA. Individual dots, represent the mean SCR of three independent technical IFA replicates from one culture; the horizontal line and error bars indicate the median SCR and IQR of all cultures from the same HBB genotype. **(D)** Initial parasitemia at 0 hpc by HBB genotype Statistical tests: C, Beta-binomial GL(M)M with per-genotype dispersion parameter and Bonferroni correction for pairwise comparisons; D, Kruskal-Wallis test. Significant (*p* < 0.05*, *p* < 0.01**, *p* < 0.001***) and non-significant p-values (ns). Abbreviations: AF, Alexa Fluor; DAPI, 4’,6-diamidino-2-phenylindole; *ev*SCAs, *ex vivo* sexual conversion assays; GL(M)Ms, Generalized linear (mixed) models; Hb, hemoglobin; HBB, human hemoglobin beta; Hpc, hours post culture; hpi, hours post-invasion; IFA, immunofluorescence assays; MACS, magnetic activated cell separation; ML10, cGMP-dependent protein kinase inhibitor; RBCs, red blood cells; SC, sexual conversion; SCR, sexual conversion rate. Panel A was created in BioRender. Yasmina, **D.** (2026) https://BioRender.com/f0i2udf.

**Fig 2:**
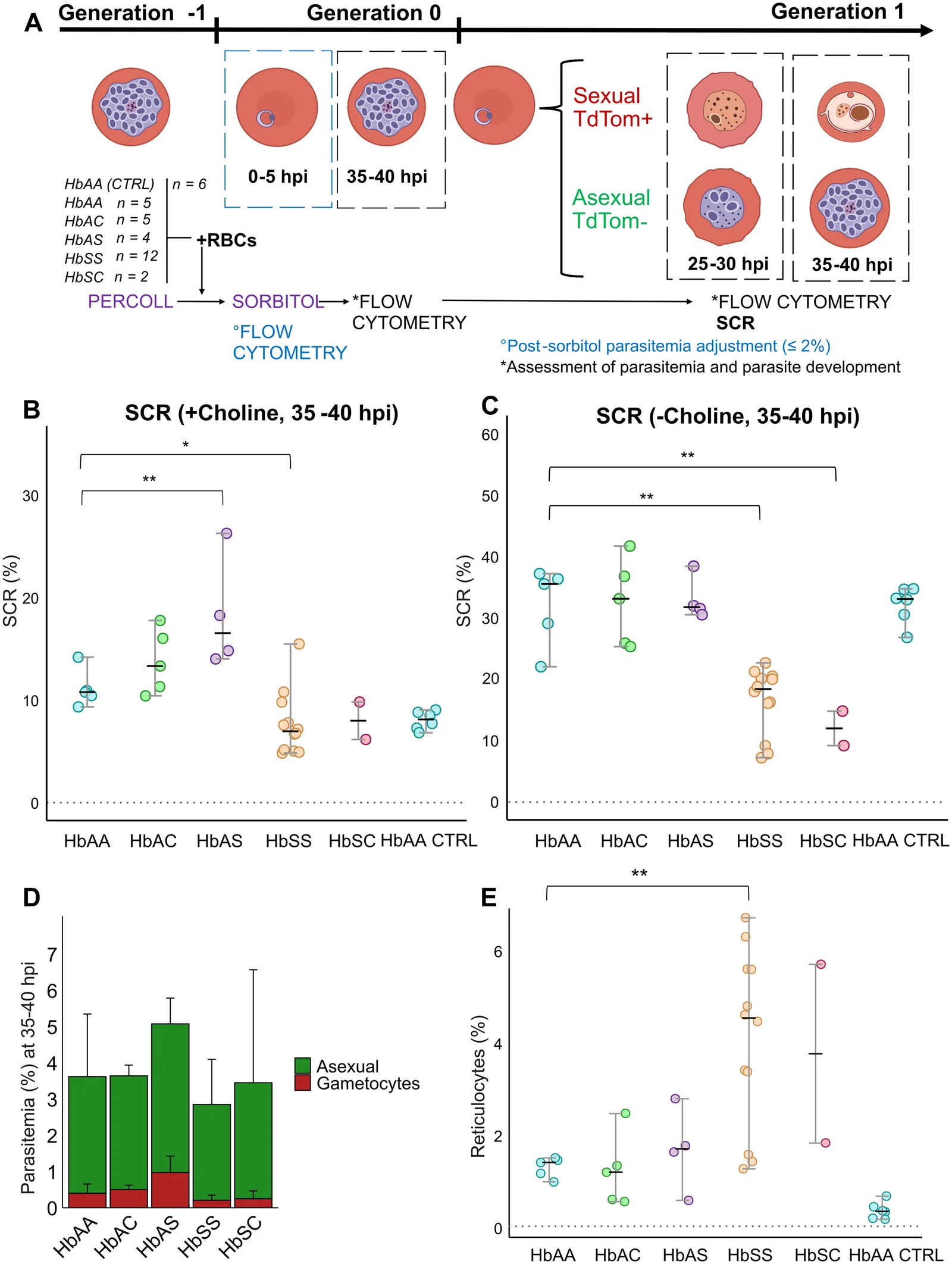
Effect of HbAA, HbAC, HbAS, HbSS and HbSC on SC by *iv*SCAs. **(A)** Experimental design of *in vitro* SC assays (*iv*SCAs) using the NF54*-gexp02-Tom P. falciparum* line. Tightly synchronized schizonts (Generation -1) were allowed to reinvade red blood cells (RBCs) of different HBB genotypes, and ring stages (0-5 hpi) were cultured under non-inducing (+Choline) and inducing (-Choline) conditions. Parasite development was assessed by flow cytometry at 35-40 hpi (Generation 0) and 25-30 hpi/35-40 hpi (Generation 1). SCRs were measured by flow cytometry at 25-30 hpi and 35-40 hpi (Generation 1). SCRs by HBB genotype at 35-40 hpi (Generation 1) in **(B)** +Choline cultures and (C) in -Choline cultures. **(D)** Proportion of asexual and gametocyte stages and total parasitemia by HBB genotype at 35-40 hpi (Generation 1) in +Choline cultures. **(E)** Reticulocytes percentage by HBB genotype. Statistical tests: B, C and E, Beta-binomial GLMs with Bonferroni correction for pairwise comparisons. Significant *p*-values are shown as: *p* < 0.05*, *p* < 0.01**, *p* < 0.001***; non-significant *p*-values as: ns. Abbreviations: CTRL, control sample; GLMs, Generalized linear models; Hb, hemoglobin; HBB, human hemoglobin beta; Hpi, hours post-invasion; SC, sexual conversion; SCR, sexual conversion rates. Panel A was created in BioRender. Yasmina, **D.** (2026) https://BioRender.com/ew8vbur.

Parasitemias did not significantly differ between mutant HBB RBCs (HbAC and HbAS) and wild-type HbAA RBCs (Kruskal-Wallis test, overall *p* = 0.72, Fig 1D); and SCRs and initial parasitemia did not correlate (Spearman correlation, ρ = 0.05, *p* = 0.62; (S2A Fig). These results indicate that differences in initial parasitemias do not have an impact on the observed elevated SCRs. A risk analysis of different variables in the study population (*i.e.,* gender, village, ethnicity, age, parasitemias, etc.) across HBB genotype groups, showed that SCRs is the only variable that significantly differs amongst the HBB genotypes HbAA, HbAC and HbAS (Kruskal-Wallis test, overall *p* < 0.001; S2 Table).

### *In vitro* sexual conversion in red blood cells of different HBB genotypes

To confirm the findings from the *ev*SCAs, we adapted a previously described *in vitro* SC assay (*iv*SCA) [65] to investigate SC in mutant RBCs (Fig 2A). The assay is based on the *P. falciparum* transgenic reporter line NF54-*gexp02-Tom* [65,81], which carries an integrated tandem Tomato (tdTom) fluorescent marker under the control of the promoter of the early gametocyte marker gene *gexp02*. Percoll-purified schizonts were grown in cryopreserved RBCs with different HBB genotypes from 28 wild-type and mutant donors, including 15 donors from the malaria-endemic area of Nanoro, Burkina Faso (5 HbAA, 5 HbAC, 4 HbAS, and 1 HbSC) and 13 patients with hemoglobinopathies (12 HbSS and 1 HbSC) from a non-endemic area (Antwerp University Hospital, UZA). In addition, non-cryopreserved (fresh) RBCs from 6 control HbAA donors (HbAA CTRL) from a non-endemic area were included (Fig 2A). After 5 hours, sorbitol lysis was performed to achieve tight synchronization (5 hours age window) and cultures were maintained either with (+Choline) or without (-Choline) a choline supplement, to measure basal (non-inducing) or induced SCRs, respectively [60,79,81]. At the next cycle (Generation 1), SCRs were estimated as the proportion of total parasites (identified by Hoechst33342 staining) that developed as sexual forms (TdTom positive), determined by flow cytometry [81]. A previous report described that the vast majority of sexual parasites expressed the TdTom reporter from 10-15 hours post-invasion (hpi) of Generation 1 onwards [81], and other studies using this assay typically measured SCRs at 20–25 hpi [65]. However, to account for the possible developmental delay observed in cultures grown in RBCs with mutant HBB genotypes (see next section), SCRs were systematically assessed at both 25–30 hpi and 35–40 hpi. The *iv*SCA assay (Fig 2A and S3 Fig) was validated for use with cryopreserved mutant RBCs as described in *S1 Appendix*. As detailed in the *Materials and methods*, analyses were intentionally restricted to parsimonious models due to sample size constraints, and results should be interpreted accordingly.

In non-induced cultures (+Choline), SCRs measured at 35–40 hpi were significantly higher in HbAS RBCs than in wild-type HbAA RBCs (median 16.50% vs. 10.80%, OR[95% CI] = 1.73 [1.12 − 2.66], *p* [*Bonferroni*] = 0.0056 using beta-binomial GLM) (Fig 2B, S1B and S3 Tables). The same trend was observed for HbAC RBCs, but in this case the difference was not significant (median 13.30%, *p[Bonferroni]* = 0.77 using beta-binomial GLM; S1B Table in S1 Table). In contrast, SCRs were significantly lower in HbSS RBCs (median 6.96%, OR[95% CI] = 0.64 [0.43 − 0.95], *p* [*Bonferroni*] = 0.021 compared to HbAA RBCs, using beta-binomial GLM) and non-significantly lower in HbSC RBCs (median 7.99%, *p[Bonferroni]* = 0.62, using beta-binomial GLM) (Fig 2B, S1B and S3 Tables). The fold-changes between median SCR in HbAC, HbAS, HbSS and HbSC relative to the median SCR of wild-type HbAA RBCs were 1.24 (IQR 0.44) in HbAC, 1.53 (IQR 0.53) in HbAS, 0.65 (IQR 0.29) in HbSS and 0.74 (IQR 0.17) in HbSC RBCs, reflecting the increased SCRs in HbAC and HbAS RBCs compared to HbAA. The same trends were observed when SCRs were measured at 25–30 hpi, but in this case none of the differences were statistically significant (S4A Fig, S1B and S3 Tables). However, SCRs estimated at 25–30 hpi were consistently lower than those obtained at 35–40 hpi (S3 Table), experimentally demonstrating that a proportion of sexually committed parasites had not started expressing TdTom yet at 25–30 hpi. Together, these findings indicate that SCR measurements at 35–40 hpi provide a more complete and reliable quantification of SC. This interpretation is further supported by the developmental delay analysis presented in the next section.

In cultures in which SC had been stimulated by choline depletion (-Choline), SCRs were significantly lower in cultures with HbSS or HbSC RBCs than in cultures in HbAA RBCs at both 25–30 hpi and 35–40 hpi (beta-binomial GLM; S1B Table in S1 Table; Fig 2C and S4B Fig). At 25–30 hpi, median SCRs were 24.00% in HbAA cultures, compared with 16.00% in HbSS (OR[95% CI] = 0.52 [0.35 − 0.79], *p[Bonferroni]* = 0.0003) and 10.20% in HbSC (OR[95% CI] = 0.36 [0.17 − 0.77], *p[Bonferroni]* = 0.0032) (S4B Fig, S3 Table). At 35–40 hpi, SCRs increased in HbAA cultures (median 35.50%) but remained lower in HbSS (median 18.40%, OR[95% CI] = 0.41 [0.27 − 0.61], *p[Bonferroni]* < 0.0001) and HbSC cultures (median 11.90%, OR[95% CI] = 0.30 [0.14 − 0.64], *p[Bonferroni]* = 0.0003) (Fig 2C, S3 Table). While the reduced SCRs observed in HbSS and HbSC RBCs under both basal (non-inducing) and inducing conditions may reflect impaired SC associated with these HBB genotypes, it is also possible that these results are explained by parasite developmental arrest in these RBCs. On the other hand, in choline-depleted cultures, SCRs were similar in HbAS or HbAC RBCs compared with HbAA RBCs (Fig 2C, S1B and S3 Tables), indicating that the high SCR observed after choline depletion does not further increase in mutant HBB RBCs. These findings suggest that the differences in SCR observed under non-induced (+choline) conditions become less apparent under choline depletion conditions, potentially because choline depletion strongly induces SC and masks more subtle HBB genotype-dependent effects.

The final total parasitemia (Generation 1) followed a similar trend as final gametocytemia between all genotypes, highest in cultures with HbAS RBCs and lowest in HbSS and HbSC RBCs (Fig 2D and S4C Fig). This indicates successful reinvasion of all RBC types, and confirms that the higher SCRs in HbAS reflect enhanced gametocyte production rather than reduced asexual parasite numbers. Furthermore, differences in SCRs could not be explained by variation in initial parasitemia at 0–5 hpi (Generation 0, all cultures were adjusted to a parasitemia below 2.0%), as no significant differences were found between HBB genotypes (beta-binomial GLM, *p [Bonferroni]* = 1; S1B Table in S1 Table). Additionally, no correlation was observed between SCR and initial parasitemia (Spearman ρ = 0.10, *p* = 0.63; S2B Fig). We assessed other possible confounders for the differences in SCR among HBB genotypes. A risk analysis of different variables in the study population (HBB genotype, ABO blood group, gender, village and ethnicity) confirmed that HBB genotypes are a risk factor for enhanced SCRs, in addition to gender (females) (Fisher’s exact test, overall *p* < 0.01 and *p* < 0.05, respectively; Table 1). Other RBC polymorphisms that may influence parasite development or SC, such as G6PD deficiency, were not assessed in the present study. Gender was not included in further analyses due to its imbalanced spread across HBB genotypes (S4 Table). Since reticulocytes have been proposed to enhance SCRs [63], and individuals with mutant HBB genotypes often exhibit anemia-associated reticulocytosis, we assessed reticulocyte percentages across all HBB genotypes. We observed significantly higher reticulocyte percentages in HbSS compared to HbAA RBCs (beta-binomial GLM, OR [95%CI] = 2.56 [1.20 − 5.44], *p [Bonferroni]* = 0.008, S1B Table in S1 Table and Fig 2E), which was confirmed in a risk analysis of reticulocytes, amongst other factors, across HBB genotype groups (Kruskal-Wallis test, overall *p* < 0.001; S4 Table). SCRs across HBB genotypes were negatively correlated with reticulocyte percentage (Spearman ρ = -0.50, *p* < 0.01; Table 1). Since SCR could not be readily measured in HbSS and HbSC genotypes due to parasite developmental delay/arrest in these RBCs (see next section), we performed correlation analysis without the HbSS and HbSC genotypes, only between HbAA, HbAC and HbAS. No correlation was observed between reticulocyte percentage and SCR (Spearman ρ = 0.10, *p* = 0.73; S4D Fig), suggesting that higher SCRs are likely due to intrinsic properties of mutant RBCs rather than to higher reticulocyte abundance. Next, to monitor potential RBC loss during the assays, we assessed RBC counts (RBCs/µl) across HBB genotypes at multiple timepoints, as shown in S5 Table. No significant differences were observed at any time point, suggesting that there were no major differences in RBC loss across HBB genotypes during the course of the experiment. Lastly, we analyzed inter- and intra-experimental variability across cultures with different HBB genotypes and independent experiments (*S1 Appendix*, S5 Fig). SCR variability was driven primarily by differences between individual donor samples rather than between experiments.

**Table 1:** Risk analysis of factors associated with elevated SCRs in the *iv*SCAs.

Median (IQR)	SCR at 35–40 hpi (Generation 1)	*p*-value^$^
**HBB genotype**		<0.01
HbAA	10.8 (0.49)	
HbAC	13.30 (4.71)	
HbAS	16.50 (5.64)	
HbSS	6.96 (3.17)	
HbSC	7.99 (1.83)	
**ABO blood group**		0.15
O+	7.57 (7.86)	
A+	8.80 (2.52)	
B+	17.80 (3.48)	
A-	9.34 (2.87)	
B-	10.90 (3.77)	
**Gender**		<0.05
Female	11.30 (3.75)	
Male	7.57 (4.09)	
**Village**		0.30
Nanoro	11.30 (3.75)	
Nazoanga	15.40 (0.60)	
Soum	0 (0.00)	
Séguédin	0 (0.00)	
**Ethnicity**		0.40
Gourounsi	17.80 (0.00)	
Mossi	12.30 (4.14)	
	**Median (IQR)**	***p*-value (rho)°**
**Reticulocytes (%)**	1.50 (5.63)	< 0.01 (-0.50)
**Age**	27.00 (30.00)	0.37 (-0.18)
***n* (%)**	28 (100.00)	–

Significant p-values (*p* < 0.05*, *p* < 0.01**, *p* < 0.001***). $Categorical variables test: Fisher’s exact test, Continuous variables test: Kruskal Wallis test, °Continuous variables: Spearman correlation test. SCRs measured at 35–40 hpi (Generation 1). Data from *P. falciparum* non-infected samples collected in Nanoro, Burkina Faso and the Antwerp University Hospital (UZA, Belgium) (total *n* = 34). Statistical tests performed are shown at the bottom of the figure with correspondent significant *p*-values as: *p* < 0.05*, *p* < 0.01**, *p* < 0.001***). **Abbreviations:** Hb, hemoglobin; HBB, human hemoglobin beta; hpi, hours post-invasion; IQR, Interquartile range; *iv*SCAs, *in vitro* sexual conversion assays; SCRs, sexual conversion rates.

### Red blood cells with mutant HBB genotypes delay parasite development

*P. falciparum* growing in HbAS RBCs exhibits developmental delay or arrest before DNA replication under low oxygen conditions (≤ 5% O_2_), as those in the bone marrow and spleen and in standard *P. falciparum* culture conditions as used for the *iv*SCA [25]. Because altered parasite development (delay or arrest) in a particular HBB genotype may affect the timing at which SC can be measured *in vitro,* we characterized parasite stage progression in parasite cultures containing RBCs with different HBB genotypes (HbAC, HbAS, HbSS and HbSC mutant RBCs vs. HbAA wild-type RBCs). First, parasite developmental stages (rings, trophozoites, and schizonts) were quantified using light microscopy (LM) across timepoints: 35–40 hpi at Generation 0; 25–30 hpi at Generation 1; and 35–40 hpi at Generation 1 (Fig 3A). Parallel flow cytometry analysis of the proportion of late stages (schizonts) revealed consistent patterns with LM observations (Fig 3B). We therefore used flow cytometry (gating strategy in Fig 3C) to analyze parasite development across all RBC samples.

**Fig 3:**
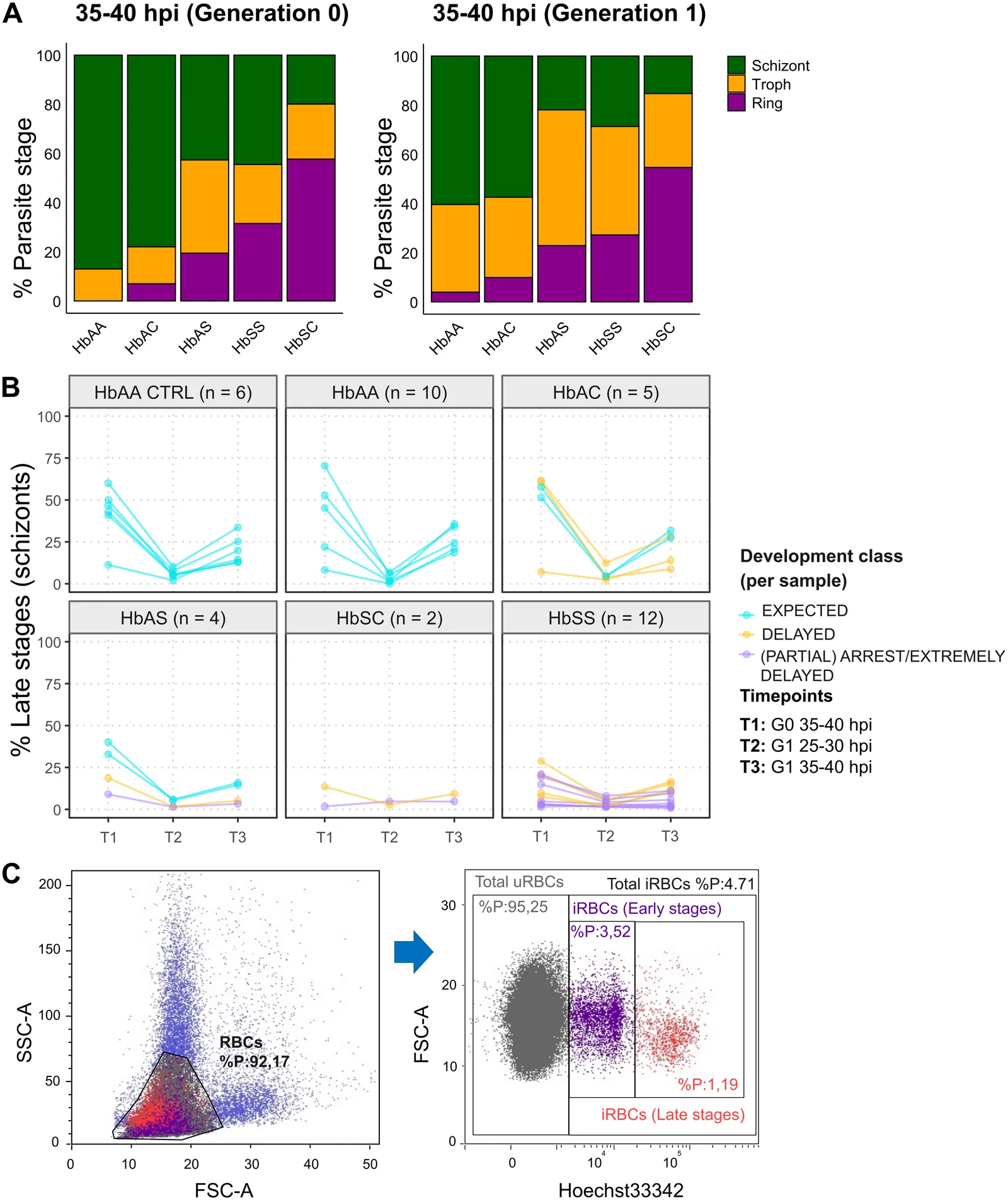
*P. falciparum* parasite development in RBCs with different HBB genotypes. (A) Microscopy-based quantification of parasite stages (rings, trophozoites and schizonts) expressed as percentages of a total of 100 counted parasites per sample. Y-axis: percentage of parasite stages (%); X-axis: HBB genotype groups (HbAA *n* = 1, HbAC *n* = 1 and HbAS *n* = 2, and HbSS *n* = 2 and HbSC *n* = 2 donors from a malaria-endemic area in Nanoro, Burkina Faso. Timepoints shown are where progression toward late stages (*i.e.,* schizont stages) is most clearly observed, namely 35–40 hpi (Generation 0; left graph and Generation 1; right graph). A shift towards earlier parasite stages indicates developmental delay.(B) Developmental progression of individual parasite cultures across timepoints 35–40 hpi (Generation 0), and 25–30 hpi and 35–40 hpi (Generation 1), shown as the proportion of late stages (schizonts) among total infected RBCs (iRBCs) measured by flow cytometry. Data are stratified by HBB genotype: HbAA (*n* = 5), HbAC (*n* = 5),HbAS (*n* = 4), and HbSC (*n* = 1) donors from a malaria-endemic area in Nanoro, Burkina Faso; and HbSS (*n* = 12), HbSC (*n* = 1) and *n* = 6 control HbAA donors [HbAA CTRL] from a non-endemic area. (C) Representative flow cytometry plots showing the gating strategy for the detection of late parasite stages. Left: total RBCs gated. Right: within the total RBCs gate in left plot, uninfected RBCs (uRBCs) and iRBCs are identified, with iRBCs further gated into early and late parasite stages by Hoechst 33342. Abbreviations: CTRL, control sample; FSC-A, Forward Scatter-Area; G0, Generation 0; G1, Generation 1; GLMs, Generalized linear models; Hb, hemoglobin; HBB, human hemoglobin beta; Hpi, hours post-invasion; RBCs, red blood cells; SSC-A, Side Scatter-Area; Timepoint abbreviations are specified in the figure legend.

Parasite developmental delay was estimated by comparing parasite stage distributions and mean parasite age with HbAA cultures, using the expected ~44 hours intraerythrocytic cycle duration to approximate developmental delay in hours. Individual cultures were classified as expected, delayed, or (partially) arrested/extremely delayed development, based on deviations from HbAA cultures across flow cytometry-derived parameters, including late-stage proportions at successive time points, their change between generations (Figs 2A and 3B, S1B Table in S1 Table), and parasite multiplication rates (PMRs) (S6 Table and S7 Fig). A detailed description of the analyses and classification criteria is provided in *S1 Appendix*, S6 Table and S7 Fig.

Cultures with very low late-stage proportions at 35–40 hpi likely reflect delayed development, with parasites remaining in earlier stages, rather than rapid development to ring stages in the next generation. Developmental delay was more pronounced in HbSC and HbSS RBCs than in HbAC and HbAS RBCs relative to HbAA RBCs (Fig 3A). Parasites in HbAA RBCs generally progressed normally from Generation 0–1 with no apparent delay; although unexpected high variability between individual donors was observed at 35–40 hpi in Generation 0 (Fig 3B). In HbAC RBCs, parasite development was slightly delayed (~3 hours vs. HbAA) but without arrest. HbAS RBCs showed a more pronounced delay (~10 hours), although most cultures continued to progress through the cycle and only one sample showed partial developmental arrest (Fig 3A-3B, S6 Table). Therefore, in HbAS and HbAC cultures, most parasites reached late stages by 35–40 hpi in Generation 0, progressed to ring stages and subsequently to late stages in Generation 1, allowing SCR measurements (at 35–40 hpi). In contrast, HbSS and HbSC RBCs presented a delay of ~9 and ~16 hours, respectively, with a higher frequency of partially arrested/delayed cultures (67% in HbSS and 50% in HbSC) (Fig 3A, S6 Table and S7 Fig). The (partial) arrest/extreme delay in HbSS and HbSC cultures may explain the apparent reduction in SCRs. Together, these findings demonstrate the suitability of measuring SCRs at 35–40 hpi in Generation 1 for HbAA, HbAS and HbAC cultures, while highlighting important limitations in HbSS and HbSC cultures.

### IgG and IgM reactivity in plasma of individuals with different HBB genotypes

We analyzed IgG and IgM reactivity against *P. falciparum* in individuals with different HBB genotypes using flow cytometry and selected plasma samples from humans living in the malaria-endemic area of Nanoro, Burkina Faso, collected as part of the *InHost* longitudinal cohort study [74] (see Materials and methods). IgG and IgM antibodies were measured against antigens on the surface of intact RBCs infected with the *P. falciparum* transgenic reporter line NF54-*gexp02-Tom* [65,81] at the early trophozoite/gametocyte stage (20–25 hpi; Generation 1; Fig 2A) using a flow cytometry protocol adapted from Dantzler KW., et al. (2019) [82]. Plasma samples from individuals living in malaria-endemic area were defined as “endemic plasma”, whereas samples from individuals living in non-endemic area were defined as “non-endemic plasma”. Detailed information on assay optimization and validation is provided in *S1 Appendix*. Flow cytometry gates were set to quantify total iRBCs (trophozoites and stage I gametocytes), asexual iRBCs (trophozoites), and sexual iRBCs (stage I gametocytes) based on Hoechst33342 and tdTomato (tdTom) fluorescence. IgG- and IgM-positive cells were identified using AF488 and PerCP-Cy5.5, respectively, resulting in six populations: total iRBCs (Hoechst + /tdTom+ or -), asexual iRBCs (Hoechst + /tdTom-), and sexual iRBCs (Hoechst + /tdTom+), each subdivided by IgG- or IgM-positivity (S6B Fig). To ensure robust ΔMean Fluorescence Intensity (ΔMFI) measurements, a minimum number of 200 IgG- or IgM-positive iRBCs (events) per gated population was required. This cut-off refers to the number of antibody-positive cells contributing to the ΔMFI calculation (S8A and S8C Fig).

In each assay, positive controls including three acute *P. falciparum*-infected non-endemic plasma samples from a traveler at days 0 (D0), 21 (D21) and 23 (D23), and a negative control (“naïve non-endemic plasma”) were analyzed. Endemic plasma samples were considered positive for IgG or IgM when both the event count and IgG- and IgM-positive total-iRBC proportions exceeded those observed in the “naïve non-endemic plasma”. The “naïve non-endemic plasma” sample consistently did not reach the 200 event counts threshold, confirming its suitability as a negative control. The negative control values served as a baseline for the IgG- and IgM-positive total iRBC proportions (% cells) and were lower than those of the endemic plasma samples of all HBB genotypes (Fig 4). Importantly, the event counts of IgM-positive total and asexual iRBCs in the positive controls at D21 and D23 exceeded both the event counts observed in the “naïve non-endemic plasma” and the threshold of 200 events, confirming that our flow cytometry assay can detect IgM-positive total and asexual iRBCs (S8C Fig). The time course of the “acute *P. falciparum*-infected non-endemic plasma at D0, D21 and D23” generally followed the expected IgG (D0 < D21 < D23) and IgM (D0 < D21 > D23) dynamics in IgG- and IgM-positive total iRBCs after a *P. falciparum* infection [83,84].

**Fig 4:**
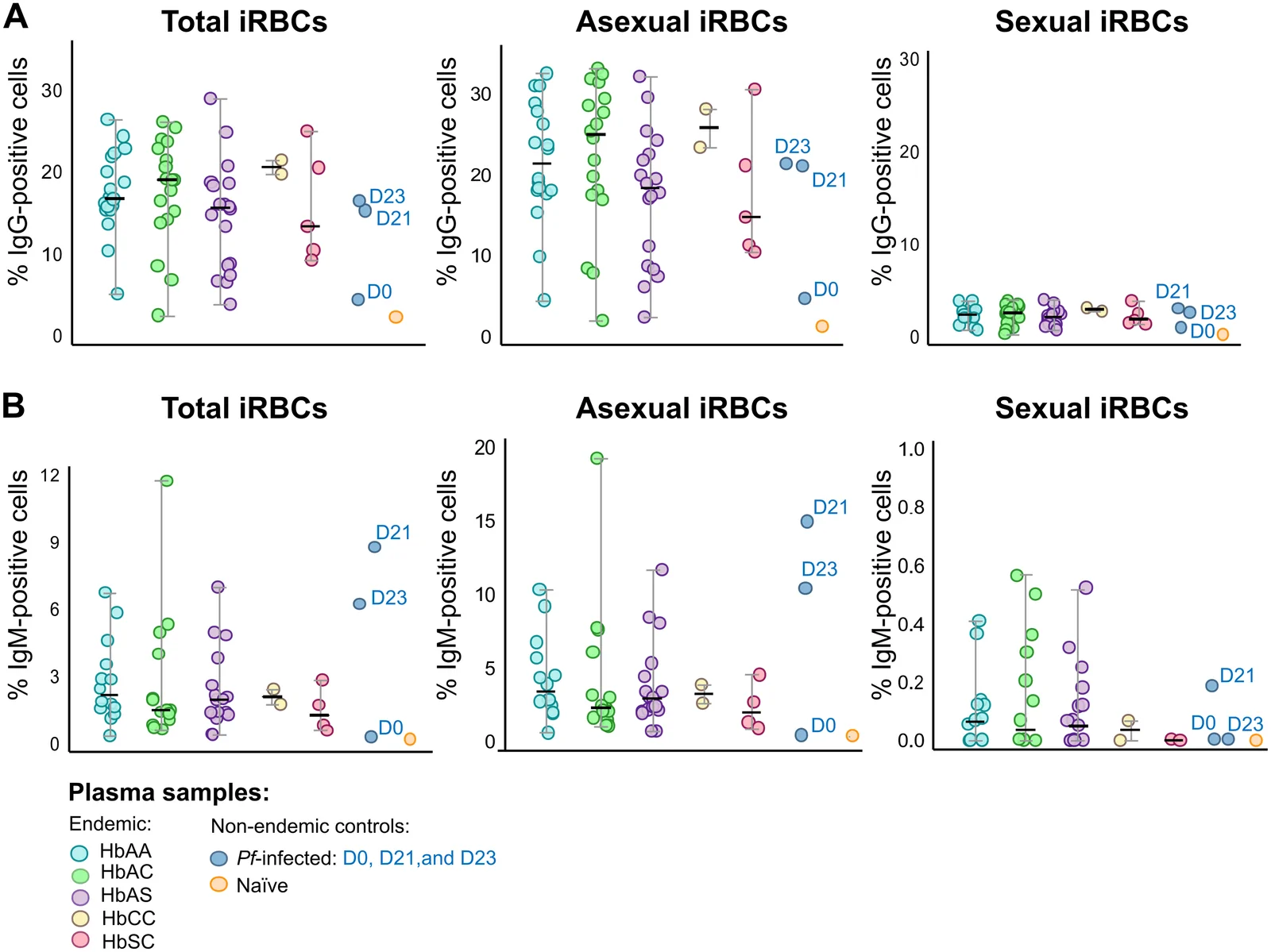
IgG- and IgM-mediated responses to *P. falciparum* trophozoites and stage I gametocytes across HBB genotypes. Data from *P. falciparum* infected (*n* = 44) and non-infected (*n* = 17) plasma samples collected at the Clinical Research Unit (CRUN) in Nanoro, Burkina Faso (total *n* = 61). **(A)** Percentage of IgG-positive iRBCs: total (left), asexual (middle) and sexual (right) iRBCs. **(B)** Percentage IgM-positive iRBCs: total (left), asexual (middle) and sexual (right) iRBCs. Positive controls (“*Pf*-infected non-endemic” *i.e.,* three acute *Pf-*infected non-endemic plasma samples from a traveler at D0, D21, and D23) and a negative control, i.e.*,* “Naïve non-endemic”: non-endemic plasma from a volunteer with HbAA genotype) are shown as individual dots per sample. The Y-axis range differs between graphs for visualization. No significant differences were found (beta-binomial GLM with Bonferroni adjustment for pairwise comparisons, *p* > 0.05). Abbreviations: asexual iRBCs, trophozoites; D, day(s); GLM, Generalized linear models; Hb, hemoglobin; HBB, human hemoglobin beta; IgG and IgM, Immunoglobulin G and M; iRBCs, infected-red blood cells; *Pf, P. falciparum*; sexual iRBCs, stage I gametocytes; total iRBCs, trophozoites and stage I gametocytes.

MFI could only be evaluated for IgG-positive total and asexual iRBCs in the statistical analyses (Fig S8B and S8D), because all endemic plasma samples (*n* = 61) exhibited event counts below 200 for the IgM and IgG-positive sexual iRBCs, thirteen samples (21%) had no detected (*i.e.,* zero) events for IgM-positive sexual iRBCs and the median event count of IgM-positive total and asexual iRBCs was below the 200 event threshold for all HBB genotype groups. The percentage of IgG- or IgM-positive iRBCs reflects the proportion of iRBCs recognized by antibodies, whereas the MFI reflects the relative level of antibody binding per positive cell. When IgM-positive sexual iRBCs were rare or absent, the proportion of positive cells could still be quantified, but MFI could not be reliably estimated due to insufficient antibody-positive events.

In endemic plasma samples, we detect low or absent IgG- and IgM-positive sexual iRBC proportions, and low or absent IgM-positive total and asexual iRBC proportions, as compared to high IgG-positive total and asexual iRBC proportions (Fig 4 and S8 Fig). We found no significant differences in the IgG- and IgM-positive total iRBC proportions (% of cells/∆MFI data are only considered robust for IgG-positive total and asexual iRBCs, reaching the minimum threshold of 200 events; Fig 4) (beta-binomial GLM, *p* > 0.05; S1C Table in S1 Table). When analyzing the response against sexual or asexual iRBCs separately, there was no significant difference across endemic plasma from individuals carrying different HBB genotypes in IgG- or IgM-positive asexual or sexual iRBC proportions (Fig 4, S7 Table) (beta-binomial GLM, *p* > 0.05; S1C Table in S1 Table).

The evaluation of variables in the study population (*n* = 61) such as: *P. falciparum* infection status at the time of sample collection, parasitemia (parasites per µl), presence of gametocytes in blood, age, gender and village; across HBB genotype groups (HbAA [*n* = 18], HbAC [*n* = 18], HbAS [*n* = 18], HbCC [*n* = 2], and HbSC [*n* = 5]) revealed no significant differences between categories (Fisher’s exact test and Kruskal-Wallis test; *p* > 0.05; S8 Table). In our study population, there is a homogeneous distribution of individuals across the categorical variables, with no observed sampling bias that could affect further analysis (S8 Table).

Further analysis of the IgG- and IgM-positive total iRBC proportions by “*P. falciparum* infection status”, showed higher antibody recognition in infected individuals (*n* = 44) compared to non-infected individuals (*n* = 17) (S9 Fig). IgG-positive total iRBC proportions were markedly higher in infected individuals (median[IQR]: 17.90[6.30]) than in non-infected individuals (9.10[9.66]) (beta-binomial GLM, OR[95%CI] = 1.81[1.35 − 2.43], *p* = 0.001; S1C Table in S1 Table). In contrast, IgM-positive total iRBC proportions showed only a modest increase in infected individuals (2.03[3.34]) compared to non-infected individuals (1.56[1.27]) (beta-binomial GLM, OR[95%CI] = 1.51[1.01 − 2.23], *p* = 0.043; S1C Table in S1 Table). These results confirm the expected IgM dynamics, where IgM-positive iRBC proportions are highest during acute or recent infections (*P. falciparum*-infected individuals) compared to past infections (non-infected individuals) [83,84].

Stratification analysis of the IgG- and IgM-positive total iRBC proportions by age-group (*i.e.,* younger age: “1 to 4 years” compared to older age: “5 to 19 years”) in *P. falciparum*-infected compared to non-infected individuals, showed that both *P. falciparum*-infected and non-infected individuals with younger age had lower IgG-positive total iRBC proportions (*Pf*-infected *n* = 22 and median[IQR]: 17.90[7.23]; non-infected *n* = 15 and median[IQR]: 9.30[10.80]) than those with an older age (*P. falciparum*-infected *n* = 22 and median [IQR]:18.60[5.64]; non-infected *n* = 2 and median[IQR]: 12.60[3.69]) (Welch’s ANOVA, *p* = 0.082 [IgG]; Kruskal-Wallis test, *p* = 0.38 [IgM]). *P. falciparum*-infected individuals with younger age had slightly higher IgM-positive total iRBC proportions (*n* = 22, median [IQR]: 2.19[2.33]) than those with an older age (*n* = 22, median [IQR]: 1.87[3.04]) (Kruskal-Wallis test, *p* = 0.94). In contrast, the IgM-positive total iRBC proportions in non-infected individuals with younger age were slightly lower (*n* = 15, median[IQR]: 1.56[1.12]) than those with an older age (*n* = 2, median [IQR]: 2.18[0.00]) (Kruskal-Wallis test, *p* = 0.16).

Overall, these findings demonstrate that the flow cytometry assay accurately detects IgM-positive asexual iRBC proportions, as validated by positive controls, whereas such responses are rare or absent in individuals from endemic areas (Fig 4). The increase of IgG with age and decrease of IgM with age explains the low IgM-positive total iRBC proportions in the endemic plasma: exposure leads to a build-up of immunity that replaces low-affinity IgM by an affinity-matured IgG response [84–88].

### *Pfsa* genotypes of parasites infecting individuals with different HBB genotypes

We performed whole-genome sequencing (WGS) on parasite DNA isolated from blood samples used in the *ev*SCA (*n* = 32), and jointly called variants in order to analyse genotypes at nine loci previously associated with reduced HbS-mediated protection against malaria *(P. falciparum sickle-associated [Pfsa+]* genotypes in bold compared to 3D7 reference): *Pfsa1+ (ACS8, chr2:631,190 T >* ***A***, *Pfsa2+ (0220300, chr2:814,288 C >* ***T***), *Pfsa3+ (1127000, chr11:1,058,035 T >* ***A***), *Pfsa4+ (FIKK4.2*, *chr4:1,121,472* ***T*** *> A*), *CLAG3.1+ (chr3: 140,167* ***T*** *> G)*, *FIKK3+ (chr3: 79,845* ***G*** *> A)*, *hyp2+ (chr2: 825,357 T >* ***C***), *PTP7+ (chr3: 96,476* ***C*** *> T)*, and *REX2+ (chr9: 1,428,875 A >* ***T***) [89–91]. All loci passed variant quality score recalibration (VQSLOD cutoff 99.5%). However, coverage, genotype quality and genotype call rates varied considerably across loci and samples (Zenodo repository: https://doi.org/10.5281/zenodo.18419143). Two samples failed genotyping across all loci due to poor overall sequencing quality. Among the retained samples, missing and low quality calls occurred at *hyp2*, *REX2*, *PTP7*, *Pfsa3* (number of missing genotypes per locus: 22, 12, 8, and 5, respectively). The *Pfsa2+* and *REX2 +* alleles were absent from the study population. *PTP7* positive, negative (wildtype), and mixed (both positive and negative alleles present) genotypes occurred at similar frequencies across HBB genotypes, indicating no apparent association between *PTP7* genotype and host haemoglobin status (Fig 5A).

**Fig 5:**
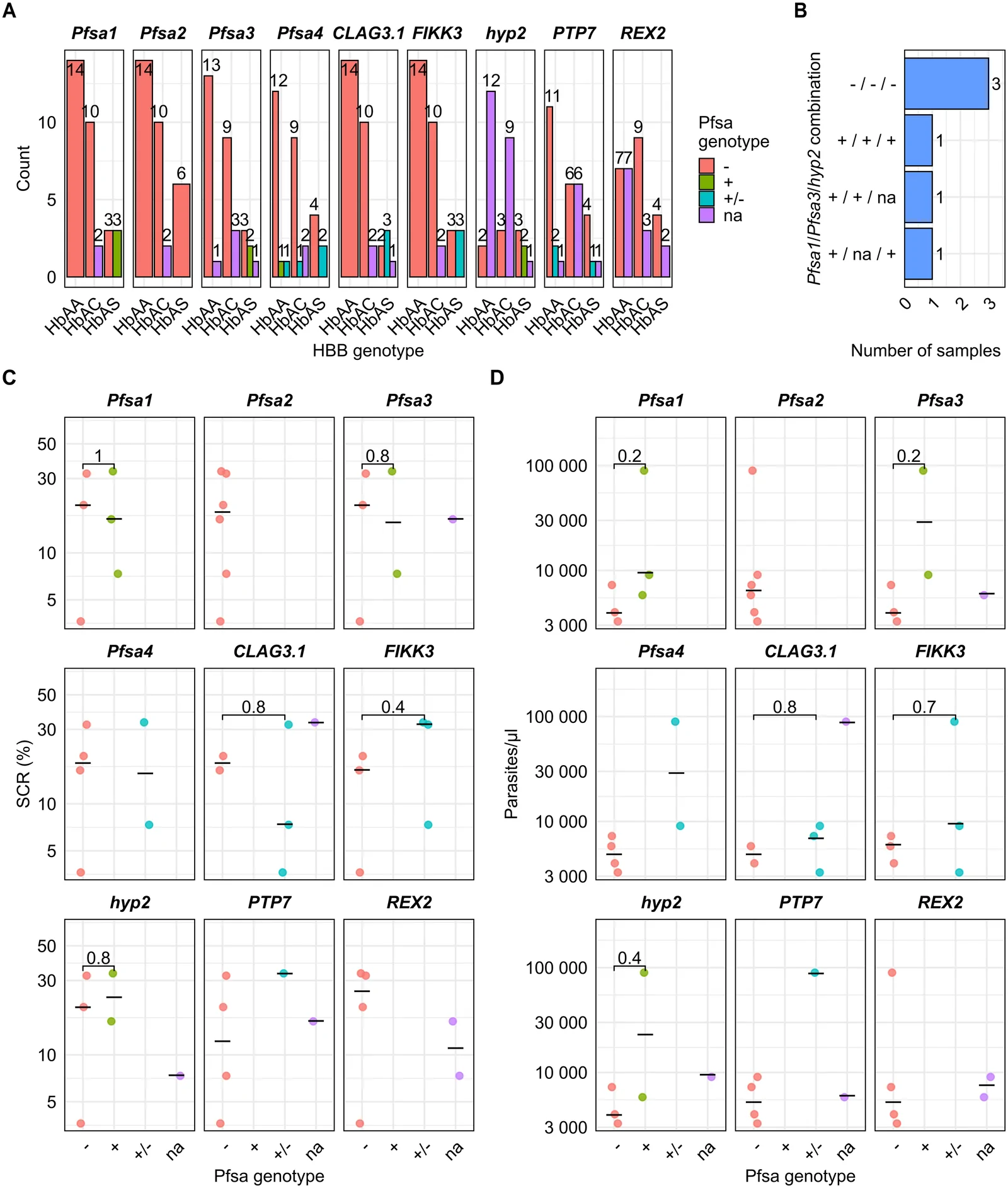
Distribution, SCR and parasite density across *Pfsa* genotypes of parasites infecting individuals with different HBB genotypes. **(A)** Numbers of samples with different parasite *Pfsa* genotypes across HBB genotypes. **(B)** Numbers of combinations of *Pfsa1, Pfsa3* and *hyp2* parasite genotypes in HbAS. **(C)** SCRs determined by *ev*SCA and parasite *Pfsa* genotype in HbAS. **(D)** Initial parasitemia at 0 hpc in samples used for *ev*SCA by parasite *Pfsa* genotype in HbAS. Statistical tests: Wilcoxon rank-sum test. Given small sample sizes per group (*n* ≤ 3), the minimum achievable two-sided *p*-value (*n* = 3 *vs n* = 3, complete separation) is 0.1; *p*-values are reported for completeness but cannot reach conventional significance thresholds at this sample size. Low quality calls occurred at *hyp2, REX2, PTP7, Pfsa3*. Abbreviations: Hb, hemoglobin; HBB, human hemoglobin beta; *ev*SCAs, *ex vivo* sexual conversion assays; SCR, sexual conversion rate; + , positive (*P. falciparum* sickle-associated [Pfsa+] genotypes; -, negative (wildtype, not equal to reference for *Pfsa4*, *CLAG3.1*, *FIKK3*, and *PTP7*); + /-, mixed = two different alleles present; na, missing calls due to missing sequencing coverage.

By contrast, *Pfsa1*, *Pfsa3*, *CLAG3.1*, *FIKK3*, and *hyp2* showed marked differences in genotype distribution between HbAA and HbAS samples (Fig 5A). *Pfsa1 +* , *Pfsa3 +* , and *hyp2 +* genotypes occurred exclusively in HbAS samples, with all HbAA samples negative (wildtype) at these three loci. Similarly, all HbAA samples were *CLAG3.1-* and *FIKK3-*, whereas only HbAS samples showed a mixed genotype at these two loci. The *Pfsa4* mixed genotype appeared more frequently but not exclusively in HbAS (2/6) compared to HbAA (1/14), with the remaining samples showing the negative genotype. *Pfsa1 +* , *Pfsa3 + ,* and *hyp2 +* genotypes co-occurred within the same HbAS samples, with two exceptions in which sequencing coverage was insufficient to determine the *Pfsa3* and *hyp2* genotype (Fig 5B).

Within HbAS samples, SCR (as measured by the *ev*SCA) was similar between positive and negative (wildtype) genotypes at *Pfsa1*, *Pfsa3*, and *hyp2* (mean SCR 19.0% *vs.* 18.7% for *Pfsa1*, 20.3% *vs.* 18.7% for *Pfsa3*, and 24.9% *vs.* 18.7% for *hyp2*; positive *vs.* negative, respectively) and between negative and mixed genotypes for *Pfsa4 (*20.3% *vs.* 18.7%). For *CLAG3.1* and *FIKK3*, mean SCR differed between mixed and negative genotypes, but in opposite directions (14.5% *vs.* 18.5% for *CLAG3.1*, and 24.3% *vs.* 13.4% for *FIKK*; mixed *vs.* positive, respectively) (Fig 5C, S9 Table). In HbAS samples, parasitaemia (total parasitaemia on day 0) was 7–10-fold higher in infections with *Pfsa1 +* , *Pfsa3 + , Pfsa4 + /-* and *hyp2 +* parasites than in their negative (wildtype) counterparts (Fig 5C, S9 Table). Sample sizes were too small to support formal statistical testing of these comparisons.

## Discussion

This study demonstrates that *P. falciparum* SC is significantly influenced by host hemoglobin genotype. SC was higher in HbAS RBCs than in wild-type HbAA RBCs both in natural infections *in vivo* (in the *ev*SCAs) and *in vitro* (using the *P. falciparum* transgenic reporter line NF54*-gexp02-Tom*), whereas a slight increase was observed for HbAC RBCs *in vivo* that could not be confirmed *in vitro*. However, we found no differences in IgG or IgM responses against trophozoite- and early gametocyte-iRBC surface antigens across HBB genotypes. Together, these findings indicate that host RBC intrinsic properties, rather than humoral immunity, underly altered parasite reproductive strategies in HBB mutant backgrounds.

Amoah et al. (2020) [92] analyzed the effect of HBB genotype on SC of field isolates from Ghanaian children aged 6–15 years with uncomplicated malaria, and reported a non-significant trend of decreasing SCRs from HbAA to HbAC and HbAS. The discrepancy with our results is most likely methodological, because quantifying SC in natural infections is challenging as parasites in freshly collected blood include morphologically indistinguishable sexual and asexual rings. The authors used prolonged culture (≥ 4 days) to distinguish sexual stages from asexual forms, similarly to Usui et al. (2019) [49]. The limitation of this approach is that SCRs are measured as the ratio of stage II gametocytes on day 4 (D4) by the total parasitemia on day 0 (D0). This can underestimate SCR because parasites contributing to the denominator (D0) are counted before culture adaptation affects viability, whereas the numerator (D4) includes only gametocytes that survive and mature after several days in *ex vivo* conditions, during which parasite loss can occur.

We addressed these limitations by adapting an *ev*SCA to quantify SC [45] for experiments with mutant RBCs. We measured whether infection of individuals with RBCs with different HBB genotypes leads to alterations of SCR. We quantified the proportion of parasites in patient samples that differentiated as asexual or sexual forms after *ex vivo* maturation. Quantification is done by immunofluorescence, with stage I gametocytes detected by Pfs16 IFA and overall parasites by DAPI [80]. Key aspects for the success of our new *ev*SCA were the inclusion of the PKG inhibitor ML10 that prevented reinvasion, allowing numerator and denominator measurements for the SCR at the same time point, while enrichment using LD columns improved sensitivity. This approach reliably quantified SC in natural human infections with densities as low as 0.05% (~2,500 parasites/µl) and detected a minimum of 38 stage I gametocytes per sample, with no gametocyte-negative cultures.

We confirmed the *in vivo* findings in the *ev*SCAs using an *in vitro* SC assay (*iv*SCA) with a gametocyte reporter line [81], which allows early detection of sexual rings. In this assay, parasites growing in HbAS RBCs showed higher SCRs than those in HbAA RBCs, whereas parasites in HbSS and HbSC RBCs were associated with lower SCRs *in vitro*. For HbAC RBCs, a slight increase was observed although not statistically significant. The fold-change SCR observed for HbAS relative to HbAA RBCs in the *iv*SCA was consistent with that reported by Flage, B., et al. (2023) [32] (1.5 vs. 1.5 in HbAS), although the results are not directly comparable due to clear methodological differences. In our assay, SCR was defined as the proportion of *gexp02*-expressing parasites among total parasites in the next cycle (generation 1), whereas in Flage, B., et al. (2023) [32], sexual parasites were quantified at generation 1 and total parasitemia at generation 0, precluding direct comparison. For HbSS and HbSC RBCs, strong conclusions regarding SCR cannot be drawn, as parasite development appeared to be impaired after reinvasion and no corresponding data from natural infections were available. This contrasts with findings in Flage, B., et al. (2023) [32], who reported markedly higher SCRs in HbSS RBCs (35% vs. 6.96% in our study) and no apparent developmental delay at day 2 of culture, although this was not quantitatively assessed. Therefore, methodological differences in SCR quantification likely contribute to the discrepancies observed between studies.

Parasite developmental progression differed across HBB genotypes. We observed a partial developmental delay in parasites infecting HbAS and HbAC RBCs, and a more pronounced arrest or extreme delay in HbSS and HbSC RBCs under low-oxygen conditions (5% O_2_), as assessed by both light microscopy and flow cytometry, consistent with Archer, N.M., et al. (2018) [25]. Later time-point measurements at 35–40 hpi resulted in higher SCRs in endemic HbAA, HbAC, and HbAS RBCs compared with 25–30 hpi, suggesting delayed sexual marker expression and indicating that the 35–40 hpi window more accurately captured SCR following parasite developmental progression. In contrast, parasites grown in control non-endemic HbAA RBCs showed stable SCRs across both time points. The differences observed between endemic and non-endemic HbAA RBCs may reflect unmeasured host genetic factors, such as blood group variants or other RBC polymorphisms.

Under choline-depleted conditions, SCRs increased markedly in HbAA, HbAC, and HbAS RBCs, reducing the differences observed under choline-supplemented conditions. This suggests that strong induction by choline depletion may mask more subtle HBB genotype-dependent effects on parasite SC. Consistent with this interpretation, Portugaliza et al. (2020) [65] showed that dihydroartemisinin-induced SC under choline-supplemented conditions but not under choline-depleted conditions, indicating that parasites already induced by choline depletion cannot be further stimulated. Similarly, our findings suggest that choline depletion may already induce SC to a level at which additional enhancement by HBB genotype-dependent effects is no longer observed. In contrast, SCRs in HbSS and HbSC under choline-depleted conditions, increased only modestly (from <10% to 10–20%) and remained below levels observed in HbAA, HbAC, and HbAS RBCs (>30–40%). This likely reflects the severely impaired intracellular environment and developmental delay causing under-detection of sexually converted stages at the measured time points, rather than inhibited sexual commitment *per se* [25,32–34]. These results indicate that accurately assessing SC in HbSS and HbSC RBCs is challenging with this experimental approach due to impaired parasite development.

We further evaluated host-related factors previously associated with SC, including reticulocyte abundance, parasitemia, and ABO blood group [63–65,93–97], but none of these potential confounders influenced SCRs in either the *ev*SCA or *iv*SCA. These results indicate that the observed increase in SC is specifically associated with HBB genotype rather than these other host-related variables. Interpretation of risk/GL(M)M analyses was limited by the small sample size and unbalanced group structure in terms of potential confounding factors, which constrained model complexity and precluded fully adjusted multivariable analyses. Although residual confounding or interactions cannot be excluded, the direction and magnitude of the observed HBB genotype effect were largely consistent across models, supporting its biological relevance.

The increase in SC observed in mutant HBB RBCs under *in vitro* conditions, in the absence of plasma and white blood cells, supports a role for RBC-intrinsic factors rather than immune or soluble host factors. Previous studies have shown that HbS and HbC RBCs are associated with altered intracellular environments characterized by increased oxidative stress, elevated free heme, impaired parasite development, altered actin remodeling, and reduced cytoadherence [25,27,28,31,32,34,35,98–100]. In particular, Flage, B., et al. (2023) [32] demonstrated that free heme can directly stimulate SC *in vitro*.

In this context, altered redox balance and intracellular stress conditions in mutant HBB RBCs may contribute to the increased SC observed in HbAS and HbAC RBCs. However, the present study was not designed to directly test these mechanisms, and additional experimental studies will be required to establish causality. The stronger effect observed *in vivo* compared with -*in vitro* further suggests that additional host or parasite factors not captured *in vitro* may also modulate SC. These may include parasite genetic diversity, host metabolic factors, or additional RBC polymorphisms [49,80,101].

To further investigate potential *in vivo* influences, we assessed the humoral immune response in the different HBB genotypes. Altered antigen presentation, cytoadherence, and increased splenic clearance [35] could plausibly lead to a modified immune response in HbAC and HbAS. However, epidemiological studies examining immunity in individuals with HbAS and HbAC genotypes have yielded conflicting results. Older children with HbAS have been shown to be more protected against the establishment of parasitemia, suggesting an acquired mechanism of protection [29]. In contrast, another study found no differences in antibody responses between individuals with HbAA, HbAS, and HbAC genotypes against their panel of 491 immunoreactive *P. falciparum* antigens [102]. More recent studies have shown that serological responses tend to be different in individuals with HbAC and HbAS when focusing on specific surface antigens and populations [70–73]. Given that differences in serological responses appear specific to certain surface antigens, we chose to measure IgG and IgM reactivity against intact iRBCs. This approach enables the assessment of immune responses to native surface antigens, minimizing potential artifacts arising from non-native protein folding, glycosylation, or conformation. We focused on trophozoite-iRBCs (25–30 hpi), as surface antigen presentation (*i.e.,* PfEMP1 expression) begins around 16 hpi and plateaus at approximately 24 hpi [103]. Moreover, commitment to SC occurs between 17–37 hpi [45,65], suggesting that any antibody-mediated induction of sexual commitment would need to occur at this stage.

We compared IgG and IgM responses to trophozoite- and gametocyte-iRBCs using plasma samples from Burkinabé individuals carrying HbAA, HbAC, HbAS, HbCC, and HbSC genotypes, assessed by flow cytometry. Our analyses revealed no significant differences in IgG or IgM reactivity against trophozoite- and stage I gametocyte-iRBCs across genotypes. IgM responses, as well as responses against gametocyte-iRBCs, were generally low or absent. Therefore, we could not confirm genotype-dependent differences in humoral responses to these parasite stages and consider it unlikely that antibodies directly drive the differential SC observed in these individuals. However, our analysis was limited to the antigens expressed by the parasite reporter line used in the assay. Responses against the immunodominant variant surface antigen PfEMP1 are notoriously difficult to decipher in *in vitro* systems as it is encoded by approximately 60 different *var* genes which are expressed mutual exclusively [104] and expression is significantly reduced *in vitro* compared to *in vivo* [105]. Moreover, we cannot exclude the possibility that genotype-specific antibody responses to later parasite stages, particularly during gametocytogenesis, as suggested by recent studies on gametocyte antigenicity [82,106], could influence transmission potential, either by promoting clearance of developing gametocytes in the human host or by inducing gametocyte sterility within the mosquito vector [107].

We next considered whether parasite genetic factors, might instead account for these differences. We performed WGS on parasite DNA isolated from the *ev*SCA samples and analysed genotypes across loci previously reported to be associated with reduced HbS-mediated protection against malaria—*Pfsa1 + , Pfsa2 +* , *Pfsa3+* and *Pfsa4 +* which have been reported in association with HbS by multiple studies [89–91,108,109]*,* and putative HbS associated loci *CLAG3.1*, *FIKK3*, *hyp2, PTP7,* and *REX2* reported in one preprint [91]. No *Pfsa2* + alleles were detected in any of the samples, consistent with the literature describing *Pfsa2*+ as extremely rare in West-Africa [90,91]. The *Pfsa1 +* , *Pfsa3 +* , and *hyp2 +* genotypes occurred exclusively among HbAS samples, with all HbAA samples negative at these loci, while *CLAG3.1* and *FIKK3* showed a different pattern, with all HbAA samples negative and only HbAS samples displaying a mixed (–/+) genotype. Within HbAS samples, however, SCR did not differ meaningfully between *Pfsa*-positive/mixed and *Pfsa*-negative infections at *Pfsa1*, *Pfsa3*, *Pfsa4* or *hyp2*, suggesting that the more established *Pfsa1*, *Pfsa 3* and *Pfsa4* loci [89–91,108,109] and *hyp2* are unlikely to underlie the elevated SC we observed in HbAS. Findings at *CLAG3.1* and *FIKK3* were less consistent, with SCR differing in opposite directions between mixed and positive genotypes; given the small number of samples and the comparatively weaker evidence linking these two loci to the HbS allele [91], we interpret this signal with caution as the small sample sizes precluded statistical testing. However, our complementary *in vitro* findings (using the same parasite line across HBB genotypes) suggests an *Pfsa+* independent effect of HbAS on SC.

Notably, parasite density was substantially higher (7–10-fold) in HbAS infections carrying *Pfsa1 +* , *Pfsa3 +* , *Pfsa4 + /-* and *hyp2 +* parasites than in their genotype-negative counterparts. This may explain why they are more frequently detected in HbAS compared with HbAA individuals. Because our study applied a parasite-density threshold of 0.05% iRBC for inclusion, it is possible that this criterion preferentially captured genotype-positive parasites within the HbAS group. We propose that the higher parasite densities associated with these genotypes in HbAS individuals could, in turn, help explain their reported association with severe malaria in the context of HbAS [89]. Such elevated densities might reflect more efficient invasion, survival, or intra-erythrocytic multiplication of these parasites within HbAS erythrocytes relative to genotype-negative parasites. Although ongoing research continues to characterise the biology of the proteins encoded at these loci [110], a conclusive mechanism underlying the protective effect of HbAS against severe malaria has yet to be established. Taken together, these results indicate that the increased SC observed in HbAS is more likely driven by RBC-intrinsic host factors than by either humoral immunity or parasite genetic background at these specific loci

Our study has some limitations that should be acknowledged. First, sample size was limited for some HBB genotype groups. Because the *ev*SCAs requires a minimum parasite density for reliable culture and downstream analyses, infections with very low parasitemia (frequently observed in malaria-protective HBB genotypes such as HbAS [25]) were necessarily excluded and thus, reduced the study sample size. Nevertheless, despite these constraints, the increased SCR observed in HbAS RBCs was independently supported by the *iv*SCAs performed under controlled conditions, and significant differences were detected using conservative statistical approaches. Furthermore, additional RBC polymorphisms frequently co-inherited with HbS, including G6PD deficiency [111,112], were not assessed but may also influence parasite SC. Disentangling their relative contribution will require larger cohort studies and higher-throughput approaches for measuring SC.

A second limitation is the use of cryopreserved RBCs in the *iv*SCAs. While cryopreserved RBCs have been successfully used for *P. falciparum* culture assays and provide important logistical advantages, RBC storage and cryopreservation may alter RBC physiological and metabolic properties, potentially influencing parasite development and SC [113,114]. The use of fresh RBCs from all HBB genotypes in parallel was not feasible, making cryopreservation necessary for controlled comparisons across genotypes. However, all HBB genotype groups were processed under comparable cryopreservation and thawing conditions, minimizing bias between groups. Validation experiments did not identify major genotype-specific effects attributable to cryopreservation or storage conditions, and variability in SCRs was primarily driven by differences between donor samples rather than experimental conditions (*S1 Appendix*, S5 Fig).

Future studies using frequent sampling with validated sexual ring biomarkers, such as *ap2-g* and *gexp02* [66], may facilitate larger-scale *in vivo* analyses of SC across HBB genotypes. However, the extent to which sexual ring biomarkers transcript abundance quantitatively reflects SCR in natural infections remains insufficiently characterized and warrants further investigation [66,81].

This study demonstrates elevated SC of *P. falciparum* parasites in HbAS RBCs both in natural human infections and *in vitro,* suggesting that stress-related intraerythrocytic conditions may promote SC. These observations are consistent with the ability of *P. falciparum* to adjust its reproductive investment in response to cues. In mutant HBB RBCs, elevated free heme levels have been associated with developmental delay and reduced parasite fitness [25]. Increased SC may therefore represent an adaptive response to stressful intraerythrocytic conditions, as previously suggested in studies linking heme-induced stress to an increased investment in SC [25,32,115].

Previous studies have shown that microenvironmental factors such as LysoPC depletion, nutrient limitation and exposure to antimalarial drugs can modulate malaria transmission by changing the parasite’s SCR. How factors such as free heme translate into a signal received by epigenetic regulators controlling the sexual commitment genes *ap2-g* or *gdv1* remains an open question [32,45]. At the population level, HBB mutant genotypes have been linked to increased gametocyte carriage and higher mosquito infectivity. In Burkina Faso, a two- to four-fold higher infection rates in HbAS, HbAC, and HbCC carriers was reported [36]. Similar trends were observed in Mali and Cameroon, where HbAC and HbAS individuals carried more gametocytes and were more infectious to mosquitoes than HbAA [37,40]. These population-level associations are consistent with our findings and suggest that increased SC in mutant HBB RBCs may contribute to enhanced transmission potential.

## Materials and methods

### Ethics statement

The *InHost* study, a longitudinal cohort in Burkina Faso, was approved by the ethical review board of the Comité d’Éthique pour la Recherche en Santé (CERS: 2018/10/131) in Burkina Faso, and by the IRB-ITM (1261/18) and IRB-UZA (19/06/064) in Belgium. The amendment for our nested study in the *InHost* study was approved by CERS (N°2022000140/MSHP/ MESRI/CERS), the IRB at ITM (1261/18), and the IRB at UZA (3347) in Belgium. Ethical approval for additional sample collection at the Hematology Unit at UZA was granted by the IRBs at ITM (1538/21) and UZA (3215). Written informed consent was obtained from the parents or legal guardians of participating children and from all adult participants.

To minimize the risk of releasing sensitive human genomic information, laboratory and computational procedures were implemented to reduce host DNA content in the deposited sequencing reads. Parasite DNA was enriched prior to sequencing using selective whole-genome amplification. In addition, sequencing reads were subjected to in silico host read removal using Deacon [116].

Details about reagents and tools are available in S10 Table.

### Study design

Sample collection for the present study was embedded in a longitudinal cohort study assessing the effect of host factors on transmission potential (*InHost* study, ethical advice reference 1261/18 and 19/06/064), conducted in the Nanoro health district, a rural malaria-hyperendemic region, in the Central-West of Burkina Faso [74,117]. Briefly, 871 study participants of all ages from four villages (Nanoro, Nazoanga, Séguédin and Soum) of the district were followed-up for two years (March 2019 - February 2021) through six cross-sectional surveys conducted during both high and low transmission season to assess asexual and sexual parasite carriage and risk factors. Active case detection was complemented by passive case detection at peripheral health centers.

For this study, from all participants of the *InHost* study (*n* = 871), 864 individuals were genotyped for their human HBB genotype (see section on “Hemoglobin beta sequencing”). Participants were then selected for this sub-cohort based on their genotype. A venous blood sample was collected from all selected participants, and screened for *P. falciparum* infection. Samples from *P. falciparum-*infected individuals were used to perform *ex vivo* SC assays (*ev*SCAs) and samples from non-infected individuals were preserved to perform *in vitro* SC assays (*iv*SCAs). We included all individuals carrying the HBB genotypes HbAC (*n* = 72), HbAS (*n* = 50), HbCC (*n* = 2), HbSC (*n* = 7), and a random subset of individuals with the HbAA genotype (*n* = 94) (S1 Fig). In total, 225 individuals were listed (age groups “5 - 19 years” [*n* = 166] and “more than 20 years” [*n* = 59]). Listed individuals were visited by a nurse at home and were invited to the Clinical Research Unit of Nanoro (CRUN, Burkina Faso) for screening and enrolment into the study. Population screening for *P. falciparum* infections and enrolment took place over a two-month period (August-September 2022).

At CRUN, the eligibility was assessed based on inclusion and exclusion criteria for the *ev*SCAs (*P. falciparum*-infected individuals) and *iv*SCAs (non-infected individuals). Inclusion criteria consisted of: being enrolled in the main *InHost* study, a confirmed *P. falciparum* malaria infection (for the *ev*SCAs) or a confirmed negative *P. falciparum* malaria infection (for the *iv*SCAs), willing to participate in this study and undergo additional sampling, and signed informed consent. Exclusion criteria consisted of: mixed infection with other *Plasmodium* species (only for *P. falciparum*-infected individuals in the *ev*SCAs), antimalarial treatment within the last two weeks, signs of severe malaria (*e.g.,* prostration), severe chronic illness besides hemoglobinopathies (*e.g.,* cancer, heart disease, kidney or liver disorders, HIV/AIDS, etc.), and planning to leave the study region during the study period. Of the 225 listed individuals, 162 were screened at CRUN, of whom 161 participants were selected as eligible and were included within this sub-cohort (S1 Fig). A 15 ml venous blood sample was collected from each participant. After sample collection, blood samples were screened for *P. falciparum* infections (see section on “*P. falciparum* diagnosis”). Samples with confirmed *P. falciparum* infection and parasitemia by light microscopy (LM) ≥ 0.05% (*i.e.,* ≥ 2500 parasites/µl) were processed for *ev*SCAs at CRUN (S11 Table). Blood samples negative for *P. falciparum* infection were processed and preserved for *iv*SCAs conducted at the Institute of Tropical Medicine (ITM), Antwerp (S11 Table, see section on “*iv*SCAs”). *P. falciparum*-infected individuals, as detected by LM and confirmed with *P. falciparum var* gene acidic terminal sequence (*var*ATS) quantitative PCR (qPCR), were treated with Artemether-Lumefantrine (AL) following national guidelines and were asked to return three days later to confirm infection clearance.

Sample size was calculated based on a power analysis assuming an expected effect size of 50%, a 5% significance level, and 90% power. This sample size was considered sufficient to detect differences in SC by HBB genotype, based on effect sizes reported in previous studies [63]. To account for potential variability in HBB genotype groups, a minimum of 8 participants per HBB genotype group was required. To ensure sufficient sampling and successful assays per HBB genotype group, the sample size was upscaled to a maximum of 15 participants per HBB genotype group.

To increase the number of HbSC and HbSS blood samples for *iv*SCA, additional blood samples were collected from patients with hemoglobinopathies (HbSS and HbSC) visited at the UZA, Belgium. A total of 13 adults (aged 19–37 years), routinely followed at UZA’s Hematology Unit, were invited to donate a blood sample with exclusion criteria of fever. The inclusion period was between October 2023 and April 2024.

### *Plasmodium falciparum* diagnosis

*P. falciparum* malaria diagnosis was done by RDT using the Biolinemalaria antigen P.f test (05FK50, Abbott), as recommended by the National malaria Control Programme (NMCP) in Burkina Faso and following the manufacturer instructions. In addition, malaria parasites were detected and quantified by LM following standard protocols at CRUN [118]. In brief, venous blood smears were stained with Giemsa and examined until 200 white blood cells were counted. Parasitemia was calculated based on the number of asexual parasites per white blood cells, expressed as asexual parasites per microliter of blood, assuming a white blood cell count of 8,000 per microliter. The presence of gametocytes and other *Plasmodium* species (*P. ovale and P. malariae*) was assessed in all positive blood smears [117]. Blood samples that tested positive for *P. falciparum* by both RDT and LM were classified as *P. falciparum*-infected. Samples that were negative by LM (regardless of the RDT result), were identified as non-infected (S11 Table, S1 Fig). Negative infection with *P. falciparum* was confirmed by *var*ATS qPCR, using five μl of DNA (extracted with QIAamp DNA Mini Kit, Qiagen Germany), as previously described [117,119] (S1 Fig). The limit of detection of the *var*ATS qPCR was 0.1 parasite/μl (in our hands), with samples showing Ct > 39.7 considered negative. DNA from the *P. falciparum* 3D7 strain served as a positive control, while negative controls included DNA from non-infected human blood and a no-template control (NTC).

### Sample collection and processing

At CRUN, a 15 ml venous blood sample was collected in an Acid Citrate Dextrose (ACD) tube and the residual blood in the needle was used for *P. falciparum* diagnosis by RDT and LM (see section on “*P. falciparum* diagnosis”). The sample was then immediately transported to the laboratory for processing. Samples were centrifuged (1,648 g for 5 minutes) and washed two times with RPMI to isolate the RBC pellet. RBC pellets from *P. falciparum*-infected blood samples were immediately put in culture for the *ev*SCAs (see section on “*Ex vivo* SC assays (*ev*SCAs)”. RBC pellets from non-infected samples were cryopreserved (1:3 dilution in cryopreservation solution containing 3% sorbitol and 0.65% NaCl) and stored directly at -80°C until shipment to ITM, where preserved in liquid nitrogen until use in *iv*SCAs. Two hundred microliters of whole blood were used for DNA extraction and confirmation of *P. falciparum* infection by *var*ATS qPCR (see section on “*P. falciparum* diagnosis). At UZA, 15–20 ml of non-infected venous blood samples were collected in EDTA tubes and immediately transported at 4°C to ITM for processing, done as described at CRUN. The resulting RBC pellets were cryopreserved (1:3 dilution in cryopreservation solution containing 3% sorbitol and 0.65% NaCl) by direct snap-freezing in liquid nitrogen for subsequent *iv*SCAs. Additionally, ABO and Rhesus D blood groups were determined with agglutination tests (BIO-RAD, REF: 801320, 801345, 802032).

### Hemoglobin beta sequencing (HBBseq)

In samples collected at CRUN, PCR amplification of the HBB gene (NCBI_Gene:3043) was performed to identify single nucleotide polymorphisms (SNPs) using PCR conditions adapted from Wittwer CT,. et al. (2020) [120] and MMV & WHO [121]. Primer pairs were designed to amplify a 599 bp product (S10 Table). PCR reactions were performed in a total volume of 25 µl, containing 2.5 µl of DNA template, 10 × Buffer B, 5 mM of each dNTP, 0.3 µM of each primer, 25 mM of MgCl_2_, and FIREPol DNA Polymerase (Solis Biodyne, Ref: 01-01-00500). Thermal cycling conditions were as follows: an initial denaturation at 94°C for 6 minutes, followed by 40 cycles of 60.6°C for 30 seconds, and 72°C for 1 minute, with a final extension at 72°C for 10 seconds. After Sanger sequencing, chromatograms were analyzed using BioEdit 7.2. (Informer Technologies) to identify the wild-type and the mutant HBB codons. The wild-type HbA allele was defined by the β-globin codon GAG (Glu6). The HbS allele was identified by the c.20A > T substitution resulting in the Glu6Val mutation (codon GTG), whereas the HbC allele was defined by the c.19G > A substitution resulting in the Glu6Lys mutation (codon AAG). Samples carrying both HbS and HbC mutations were classified as HbSC [122]. In samples collected at UZA, the HBB genotype sequences were determined by capillary hemoglobin electrophoresis at UZA as described elsewhere [123].

### Whole genome sequencing of parasite DNA

DNA extracted for *varATS* qPCR on blood samples used in *ev*SCAs (see section on “*Ex vivo* SC assays (*ev*SCAs)” was used for selective whole genome amplification (sWGA) to enrich the parasite fraction of the DNA [124].

Whole-genome sequencing was performed on the NovaSeq X platform to generate 150 bp paired-end reads while targeting ~ 100x parasite genome coverage (~3 Gbp).

Sequencing data were processed using the inhouse plasmovar (v0.3.0) workflow developed in Nextflow (v26.04.3) [125] leveraging the nf-core framework and resources [126]. All workflow code and configuration files are openly available at https://github.com/ITMmalaria/plasmovar [127] and this manuscript’s Zenodo repository (DOI: https://doi.org/10.5281/zenodo.18419143), respectively. The pipeline integrates read quality control, trimming, host read removal, alignment, variant discovery, and functional annotation steps. A detailed overview of the steps and options used in the pipeline is available in *S2 Appendix*.

### *Ex vivo* sexual conversion assays in *P. falciparum* field isolates

*P. falciparum*-infected fresh venous blood samples (15 ml) were collected from individuals carrying different HBB genotypes (S11 Table; HbAA *n* = 14, HbAC *n* = 12, HbAS *n* = 6; total *n* = 32) and were cultured *ex vivo* to measure SCRs in natural human infections. Assay optimization, validation, and sample selection criteria are described in *S1 Appendix* and S1 Fig. Cultures at 3% Hct and 20 ml volume were prepared by mixing 600 µl of *P. falciparum*-iRBC pellet with 20 ml of RPMI 1640 medium containing 0.5% Albumax II (Gibco, 11021–029) and 2 mM choline chloride (Sigma-Aldrich, C7527) [65,81]. Parasites at the ring stage were incubated using candle jar method under static conditions until ~24 hours post-culture (hpc). At this time, sexually committed rings present in the venous blood sample are expected to have developed into stage I gametocytes expressing Pfs16, while asexual parasites will have progressed to the schizont stage (Fig 1A). During culture, at ~5 hpc, ML10 (80 nM), a cGMP-dependent protein kinase inhibitor, was added to prevent schizont rupture and reinvasion [45,75]. Giemsa-stained smears were examined by LM to confirm that most parasites had reached either stage I gametocytes or schizonts stages. At ~24 hpc, parasites were harvested and purified using MACS LD columns (Miltenyi Biotec). In brief, cultured parasites are centrifuged (1,648 g, 5 min) and pelleted iRBCs were diluted 1:4 with culture medium, loaded onto an LD-MACS column, and washed four times with 1 ml medium to separate non-iRBCs and ring-stage-iRBCs (first elution). The column was then removed from the magnet, and stage I gametocytes and schizonts were recovered by gravity elution, followed by gentle plunger flushing twice with 3 ml medium (second elution). Recovered parasites were pelleted (1,648 g, 5 min), resuspended 1:2 with non-iRBCs, and diluted 1:2 in PBS. From the second elution, four thin blood smears (3 µl each) were prepared and left to air-dry; three were used for on-site IFAs, and one was Giemsa-stained for LM to verify MACS enrichment. Immunofluorescence assays

Immunofluorescence assays (IFAs) were performed at CRUN (Burkina Faso) as previously described [45,65,81], with modifications. Slides were stained in batches of four to eight cultures with three slides per culture, to optimize processing and staining consistency. Primary antibody mouse anti-Pfs16 (1:400, kindly provided by R. Sauerwein and T. Bousema, Radboud University) [76] and secondary antibody goat anti-mouse IgG (H + L) Alexa Fluor 488 (1:1000; Abcam, ab150113) were used, with incubations and washes under static conditions. Slides were mounted with Aqueous Fluoroshield (Abcam, ab104139) containing DAPI (0.0002%) and stored at 4°C until shipment to ITM (Belgium). At ITM, slides were visualized within six months of preparation on an ECHO Revolve epifluorescence microscope (ECHO, San Diego, CA) to determine SCRs (Figs 1B and 1C). Analysis was blinded to HBB genotype. Stage I gametocytes were identified as Pfs16-positive cells (green) and counted among a minimum of 1,000 DAPI-positive cells (blue) per slide (stage I gametocytes and schizonts). Slides with fewer than 1,000 DAPI-positive cells were excluded from further analysis during the first reading. For each culture (*n* = 35), three of the four prepared slides were stained by IFA and examined by a primary reader. Slides from three cultures were excluded due to insufficient DAPI-positive cells. From the remaining cultures (*n* = 32) a random subset of ~10% (4/32), with three IFA slides per culture, was examined by a second reader to assess inter-reader reliability via intraclass correlation coefficient (ICC) analysis, based on a two-way random effects model, absolute agreement, single measures [ICC(2,1) [128]]. For each IFA slide, SCRs were calculated as the proportion of Pfs16-positive parasites among total DAPI-positive cells. Across the three replicate IFA slides per culture, the mean SCR (±SD) was computed and used to calculate and report the median SCR (IQR) by HBB genotype (Fig 1C), statistical differences were assessed as described for proportions in section “Data analysis”. Fold-change in SCR for HbAC and HbAS was calculated relative to HbAA by dividing each mutant genotype’s median SCR by that of the wild-type. Statistical analysis was performed to assess differences in SCRs and in initial parasitemia across HBB genotypes (continuous not normally distributed variables) (Fig 2C-2D), as well as the potential confounding effect of initial parasitemia on SCRs using non-parametric correlation (S2A Fig), as described in section ”Data analysis”.

Stable Pfs16 fluorescence was confirmed by the counting of ≥1 stage I gametocyte per 10 fields. For each culture, the three replicate IFA slides were analyzed to quantify SCRs. The mean, minimum and maximum SCR were calculated across replicates. To assess inter-slide reproducibility (technical repeatability) of SCR measurements, an ICC (2,1) analysis (two-way random effects, single measures, consistency) was performed using SCR values from the three replicate slides per culture (*n* = 32). Counts of stage I gametocytes and total parasites were recorded for each IFA slide and summarized as median (Q1-Q3). From 5 out of 32 cultures (15.6%) specificity controls were performed by staining one slide per culture without primary antibody (negative controls). Stage V gametocytes (also express Pfs16), identified by their characteristic shape, were excluded from the counting as they are a product of SC occurring >10 days prior to the sexual commitment cycle under investigation. Giemsa-stained slides from each *ev*SCA were examined by LM to estimate parasite purification by MACS and confirm approximate parasite age/stage to assure parasites were sufficiently mature to express Pfs16 (starts expression at ~20 hpi [45]).

### *In vitro* sexual conversion assays

*In vitro* SC assays (*iv*SCAs) were performed as follows (Fig 2A). NF54-*gexp02-Tom* parasites [65,81] were routinely maintained with parasitemias of less than 2% in O + RBCs (3% Hct) in RPMI 1640-based medium supplemented with 0.5% Albumax II (Gibco, ref: 11021–029) and 2 mM choline chloride (+Choline; Sigma-Aldrich, C7527), analogous to LysoPC supplementation [60,79,81]. Cultures were incubated at 37°C with shaking conditions (70 rpm) in a gas mixture (5% O_2_, 5.5% CO_2_, balance N_2_). To minimize bias due to prior culture duration, all cultures were initiated from parasites of the same cryopreserved batch of an expanded culture.

To obtain cultures of a well-defined age window, we used Percoll/sorbitol synchronization (Fig 2A). Percoll-purified schizonts (Generation -1) were used to establish fresh cultures with thawed cryopreserved RBCs (day 3 post thawing) with different HBB genotypes (exposure, Generation 0), now cultured in static conditions. Thawing of RBCs was performed using a three-step protocol, sequentially adding three volumes of 3.5% NaCl solution (10 × the RBC volume each time), followed by RPMI washes and centrifugation (1,648 g, 5 min) as per standard procedure [129]. Approximately 5 hours later, when parasites have reinvaded the new RBCs, cultures were subjected to 5% D-sorbitol lysis [45,81] to obtain cultures of a defined 0–5 hpi age window. Post-sorbitol initial parasitemia was then measured by flow cytometry, and parasites were cultured in two parallel subcultures (~200 µl final volume) per sample. One sub-culture was maintained in +Choline medium (non-inducing SC conditions), and the other in choline-free medium (-Choline; inducing conditions). Cultures were maintained under these respective conditions for two generations (48 hpi, until 35–40 hpi in Generation 1, Fig 2A, hpi calculations are based on a 44 hours replication cycle empirically established for this parasite line in our lab). In each experiment we tested RBCs with at least two different HBB genotypes. Samples with different HBB genotypes were used in the experiments if the recovered RBC pellet volume after thawing was ≥ 30 µl after dilution with RPMI to 50% Hct. From a total of 20 cryopreserved non-iRBC samples from CRUN that were confirmed to be negative for *P. falciparum* by *var*ATS qPCR, 15 samples had a RBC pellet volume ≥ 30 µl after thawing and dilution in RPMI to 50% Hct (S1 Fig). The mean recovered RBC pellet volumes after thawing were: HbAA, 178 µl (range: 50 − 596 µl); HbAC, 111 µl (range: 30 − 320 µl); HbAS, 246 µl (range: 64 − 630 µl); HbSC, 558 µl (range: 35 − 1080 µl); and HbSS, 600 µl (range: 300 − 1120 µl). These samples were included within the *iv*SCAs, in addition to non-infected, cryopreserved RBC samples from the UZA HbSS and HbSC (S1 Fig). Total numbers of samples tested per HBB genotype and experiment are shown in S12 Table. Flow cytometry - analysis was used to measure parasitemia, assess parasite stage development (*i.e.,* proportion of parasites reaching the intraerythrocytic schizont stage or late-stage proportions), and quantify SC at defined time points during the assay (Fig 2A). Parasitemia was determined at 0–5 hpi (rings), together with (1) the proportion of schizont stages at 35–40 hpi in Generation 0 and 1, and (2) SCRs at 25–30 hpi (large rings/trophozoites) and 35–40 hpi (schizonts) in Generation 1 (Fig 2A). The standard timepoint to measure SC with this parasite line is 25–30 hpi to ensure detection of all sexually committed parasites, as TdTom-expression under the *gexp02* promoter starts around 10–15 hpi [81]. The 35–40 hpi timepoint was included at Generation 1 to account for potential delayed stage development in mutant HBB genotypes (see section on “Red blood cells with mutant HBB genotypes delay parasite development”). Four independent experiments were conducted, with RBC samples included in each experiment detailed in S12 Table. Each experiment included a fresh HbAA blood sample to control the effect of RBC storage after cryopreservation.

Samples for flow cytometry analysis (using a CytoFLEX LX flow cytometer, Beckman Coulter) were prepared by resuspending parasite cultures (1:3) in PBS containing 1% BSA, followed by staining with Hoechst 33342 (1:500, BD Pharmingen) in a final volume of 100 µl per well. Samples were then incubated at 37°C for 30 minutes. To measure parasitemia and proportion of schizont stages, flow cytometry analysis (with 100,000 events collected per sample) was set to detect Hoechst 33342 (350 nm laser; 450/40 filter). RBCs were gated by plotting side scatter area (SSC-A) versus forward scatter area (FSC-A), with singlets selected using FSC-H versus FSC-A. Parasites were identified within the singlet gate by Hoechst fluorescence. Hoechst-positive events were defined as exceeding background levels from unstained negative controls. To quantify SCRs, Hoechst fluorescence was combined with the fluorescent reporter TdTom (561 nm laser; 610/20 filter) that is integrated in the NF54-*gexp02-Tom* line to distinguish sexual (Hoechst + /tdTom+) from asexual (Hoechst + /tdTom-) parasites. SCRs were calculated as the proportion of Hoechst + /tdTom+ events among all Hoechst+ events.

SCRs were expressed as a percentage (median [IQR]) per HBB genotype (Fig 2B-2C and S4A-S4B Fig). Fold-changes in SCRs for HbAC, HbAS, HbSS and HbSC were calculated relative to HbAA by dividing median SCR of each mutant genotype group by that of the wild-type HbAA group. Statistical analysis was performed as described in the section on ”Data analysis”, to assess differences in SCRs (Fig 2B and S4A-S4B Fig) and potential confounding effect of post-sorbitol initial parasitemia (S2B Fig) across HBB genotypes. Reticulocyte percentages were measured for all RBC samples on the same day as Percoll synchronization (Generation -1, Fig 2A). Briefly, RBC suspensions were mixed 1:1 with reticulocyte stain (Sigma-Aldrich), incubated 15 min at room temperature, and used to prepare a thin blood smear for LM estimation of reticulocyte percentage. We determined the ABO and Rhesus D blood groups in all samples using agglutination tests with Seraclone Anti-A, Anti-B, and Anti-D (RH1) reagents (Bio-Rad).

### Parasite development in red blood cells with different HBB genotypes

Parasite stage development was evaluated during the *iv*SCAs (Fig 2A) at 35–40 hpi (Generation 0) and 25–30 hpi/35–40 hpi (Generation 1) using LM and flow cytometry. The proportion of rings, trophozoites and early/late schizonts was quantified at 35–40 hpi in Generation 0 and 25–30 hpi/35–40 hpi in Generation 1 by LM of Giemsa-stained thin blood smears (100 parasite counts per sample; Fig 3A) in a subset of RBC samples (HbAA *n* = 1; HbAC *n* = 1; HbAS *n* = 2; HbSS *n* = 2; HbSC *n* = 2). Flow cytometry was used as described earlier to determine the percentage of late stages. For this a distinct population of cells were gated with increased Hoechst 33342 fluorescence (increased DNA content due to multiplication of the nuclei in schizont stages), within the Hoechst+ cells (Fig 3B). Upon confirming consistent results between LM and flow cytometry after screening the same samples by flow cytometry, the same technique was subsequently used in all *iv*SCA samples (HbAA *n* = 5, HbAC *n* = 5 and HbAS *n* = 4, and HbSC *n* = 1 donors from a malaria-endemic area in Nanoro, Burkina Faso; HbSS *n* = 12, HbSC *n* = 1 and *n* = 6 control HbAA donors [HbAA CTRL] from a non-endemic area) to determine the proportion of late-stages (*i.e.,* schizonts) by HBB genotype (Fig 3B and 3C).

From LM data we calculated: (1) the percentage of rings, trophozoites and early/late schizonts (Fig 3A), (2) the mean parasite age using the age in hpi [130] of each stage (rings, trophozoites, schizonts) and the observed proportions; and (3) the developmental delay (in hours) by subtracting mean parasite age of the mutant genotypes from the wild-type HbAA genotype. Finally, for each genotype, we compared the developmental delay between Generations 0 and 1 by calculating the difference in mean parasite age at 35–40 hpi between both generations. From flow cytometry data, we derived six parameters to assess parasite development: (1) PMRs, (2) late-stage proportions at 35–40 hpi in Generation 0; (3) late-stage proportions at 25–30 hpi in Generation 1, (4) late-stage proportions at 35–40 hpi in Generation 1, and (5–6) fold changes in late-stage proportions between consecutive time points (fold changes 1 and 2) (S6 Table and S7 Fig). Based on these six parameters, the development of each independent NF54-*gexp02-Tom* culture was classified as: *expected development*, *delayed development* or *(partially) arrested/extremely delayed development*, according to the number of parameters outside the HbAA reference Q1-Q4 range, which was calculated on all 11 HbAA cultures including the controls (see detailed analysis in *S1 Appendix*, S6 Table and S7 Fig).

### IgG and IgM detection by flow cytometry against *Plasmodium falciparum*

To study the IgG and IgM reactivity against *P. falciparum* in individuals with different HBB genotypes by flow cytometry, we selected plasma samples from humans living in a malaria-endemic area from the *InHost* longitudinal cohort study [74]. We classified samples as “endemic” (endemic plasma) or “non-endemic” (non-endemic plasma) for malaria based on the country of origin. An “endemic” country has consistent, stable *P. falciparum* transmission, while a “non-endemic” country has no local transmission, with infections typically imported [131]. As the expected variation and magnitude of potential differences among HBB genotypes were unknown, we based the sample size on the minimum of 8 individuals per HBB genotype used in the *ev*SCAs and *iv*SCAs. A maximum of 15–20 individuals per genotype was included to ensure sufficient sampling. Selection criteria included equal representation of the most frequent genotype groups (HbAA [*n* = 18], HbAC [*n* = 18], HbAS [*n* = 18], HbCC [*n* = 2], and HbSC [*n* = 5]), *P. falciparum* infection status with a large proportion of active *P. falciparum* infections (*n* = 10–15) and a smaller proportion of non-infected individuals (*n* = 2–5), age range (1–19 years to exclude maternal antibodies), and homogeneous distribution amongst the ethnicity (all Mossi) and village (Nanoro, Nazoanga, Soum, and Séguédin) groups. S11 Table shows the number of participants per HBB genotype included in the assays. The *P. falciparum* transgenic line NF54-*gexp02*-Tom [65,81] was cultured under the same conditions as described in the *iv*SCAs section and Fig 2A, with the following modifications: (1) parasites were cultured only in wild-type HbAA fresh RBCs and (2) flow cytometry assays were performed on early trophozoite/stage I gametocyte-iRBCs at 20–25 hpi (Generation 1), following the protocol reported in Dantzler KW., et al. (2019) [82] with the adaptations described below.

Human plasma samples were pre-diluted in PBS with 1% Bovine Serum Albumin (BSA, Sigma Aldrich) and heparin (2,500U; Sigma Aldrich) (PBS-BSA-Hep) at dilutions of 1:5000 (IgG) and 1:100 (IgM). *In vitro* generated trophozoite- and stage I gametocyte-iRBCs (25–30hpi; Generation 1) were pre-diluted to 0.6% Hct in PBS-BSA-Hep. Pre-diluted human plasma samples were incubated at a 1:10 dilution with pre-diluted trophozoite- and stage I gametocyte-iRBCs, in separate 96-well plates for IgG and IgM assays for 1.5 hours at room temperature. After 2 washes with PBS-BSA-Hep, secondary antibodies were added to the trophozoite- and stage I gametocyte-iRBCs for 30 minutes at 4°C at 0.6% hematocrit. AlexaFluor488 (AF488) mouse anti-human IgG (Jackson ImmunoResearch Europe) and PerCP/Cy5.5 mouse anti-human IgM (Abcam) were used at final dilutions of 1:640 and 1:320 in PBS-BSA-Hep for the IgG and IgM assays, respectively. After 2 washes with PBS-BSA-Hep, the DNA stain Hoechst33342 (BD Pharmingen) was added to the trophozoite- and stage I gametocyte-iRBCs at a final dilution of 1:5000 in PBS-BSA-Hep at 0.6% hematocrit for 30 minutes at 37°C. To verify that the trophozoite- and stage I gametocyte-iRBCs measured contained exclusively RBCs (no white blood cells), each batch pre-diluted trophozoite- and stage I gametocyte-iRBCs (to 0.6% hematocrit in PBS-BSA-Hep) were stained with APC anti-human CD236 (Glycophorin C) Recombinant Antibody (Biolegend) in triplicate at a 1:40 final dilution (S6C Fig). Immunostained samples were analyzed on the flow cytometer CytoFLEX LX (Beckman Coulter), collecting 100,000 cells per culture. We analyzed the median proportions (% cells) of total (trophozoites and stage I gametocytes), asexual (trophozoites) and sexual (stage I gametocytes) iRBCs that were positive for IgG and IgM binding to surface antigens on iRBCs. Samples were considered positive for IgG or IgM if the total iRBC proportions exceeded that of a naïve non-endemic plasma from a volunteer (negative control with HbAA genotype with no prior *P. falciparum* infection, from a non-endemic country [*i.e.,* Belgium] collected at the clinic of the Institute of Tropical Medicine [ITM] in Antwerp). Three non-endemic samples from an acute *P. falciparum*-infected traveler collected at the ITM clinic at days 0, 21 and 23 of *P. falciparum* diagnosis (*i.e.,* acute *P. falciparum-infected* non-endemic plasma D0, D21 and D23) were used as positive controls. A schematic of the gating strategy to measure the surface reactivity of asexual (tdTom-) and sexual (tdTom+) iRBCs (Hoechst33342+) to IgG (IgG-positive trophozoite-iRBCs: Hoechst33342 + /tdTom-/AF488+ and IgG-positive stage I gametocyte-iRBCs: Hoechst33342 + /tdTom + /AF488+) or IgM (IgM-positive trophozoite-iRBCs: Hoechst33342 + /tdTom-/PerCP-Cy5.5.+ and IgM-positive stage I gametocyte-iRBCs (Hoechst33342 + / tdTom + /PerCP-Cy5.5.+) in the analyzed endemic human plasma samples is represented in Fig S6B and S6C.

To determine the optimal plasma dilution factor, a pool of five endemic and hyperimmune plasma samples from *P. falciparum*-infected individuals in a sub-cohort within the *InHost* study [74] (*i.e., P. falciparum*-infected hyperimmune endemic plasma pool of symptomatic individuals with high *P. falciparum* parasitemia ranging from 45,667–175,000 parasites per µl) [66] was titrated for the IgG and IgM assay separately. For the IgM assay, four additional plasma samples were titrated (*i.e.,* “naïve non-endemic plasma” and “acute *P. falciparum*-infected non-endemic plasma at D0, D21 and D23”) (S6A Fig).

During flow cytometry analysis, the IgG- (Hoechst33342 + /tdTom-/ + /AF488+) and IgM-positive (Hoechst33342 + /tdTom-/ + /PerCP-Cy5.5.+) total iRBC proportions for each sample were expressed as total event counts, percentage of positive cells, and median fluorescence intensity (MFI) per HBB genotype group. The IgG- and IgM-positive iRBC proportions and signal were compared by HBB genotype group for the total (trophozoites and stage I gametocytes), asexual (trophozoites) and sexual (stage I gametocytes) iRBCs (Fig 4 and S8 Fig). To correct for the background signal from the non-iRBCs in the IgG and the IgM channel, the MFI of all samples (including the positive and the negative controls) was expressed as a ΔMFI. For the asexual and sexual iRBCs, ΔMFI was calculated as ΔMFI (AF488 + /PerCP-Cy5.5.+) = (“MFI IgG+/IgM+ of asexual [tdTom-]/sexual [tdTom+] trophozoite-iRBCs [Hoechst33342+]”) - (“MFI IgG+/IgM+ of non-iRBCs [tdTom-/Hoechst 33342-]”) within each plasma sample. For the total iRBCs, ΔMFI was calculated as ΔMFI (AF488 + /PerCP-Cy5.5.+) = (“MFI IgG+ or IgM+ iRBCs [Hoechst33342+]”) - (“MFI IgG+ or IgM+ of non-iRBCs [Hoechst33342-]” of trophozoite stages incubated with PBS-BSA-Hep, instead of plasma).

### Data analysis

All flow cytometry data were analyzed using FlowLogic version 8.7 (Inivai Technologies). Statistical analyses were performed in R version >4.5.1 (R Core Team 2025), RStudio software version 2025.09.1 + 401(2025 Posit Software; RRID:SCR_000432) and the following R packages: brms v. 2.23.0 [132–134], DHARMa v. 0.4.7 [135], emmeans v. 1.11.2.8 [136],ggplot2 v.4.00 [137], glmmTMB v. 1.1.13 [138,139], here v. 1.0.2 [140], lme4 v. 1.1.37 [141], performance v. 0.15.2 [142], renv v. 1.1.5 [143], rmarkdown v. 2.30 [144–146], scales v. 1.4.0 [147], sjPlot v. 2.9.0 [148], tidyverse v. 2.0.0 [149], with p-values (*p* < 0.05) considered significant.

Risk analysis was conducted to examine associations of demographic and clinical variables (*e.g.,* gender, age, village, ethnicity) across HBB genotype groups (S2, S4 and S8 Tables for the *ev*SCAs, *iv*SCAs, and IgG and IgM detection, respectively). An additional risk analysis for factors associated with elevated SCRs included the same covariates with the addition of HBB genotype (Table 1, for the *iv*SCAs). Continuous variables were analyzed using the Kruskal-Wallis test when parametric assumptions were not met, and when significant, this was followed by Wilcoxon rank sum post-hoc tests for pairwise comparisons with Holm correction. Otherwise, One-way ANOVA or Welch’s ANOVA was applied depending on whether Bartlett’s test indicated equal or unequal variances, respectively. When significant, Bonferroni-corrected post-hoc pairwise comparisons were performed. Associations between categorical variables were evaluated using Fisher’s exact test. Spearman correlation was used for correlation between continuous variables with no normal distribution.

For modelling SCR, parasite stage proportions, initial parasitemia and reticulocyte proportions in *ev*SCAs, and *iv*SCAs, and IgG/IgM proportions in the flow cytometry assays, the statistical analysis type was chosen based on the nature of the variables: proportions based on counts were assessed using beta-binomial generalized linear models (GLM) and mixed models (GLMM) with Bonferroni adjustment for the odds ratio contrasts in pairwise comparisons. We chose beta-binomial models due to (possible) violations of the assumptions of the binomial model (namely overdispersion). For *ev*SCA, random factors were used to account for technical replicates and a per-HBB-genotype dispersion factor was added to the GLMM model to account for heteroskedasticity. To address the liberal parametric Wald Z estimates and confidence intervals (asymptotic approximations) provided by the Generalized linear (mixed) models [GL(M)M models], we also computed more conservative Bayesian estimates, odds ratios and credible intervals using a Markov chain Monte Carlo (MCMC) approach when model specification and convergence allowed. GLM and GLMM model specifications and analysis results are reported in S1A-S1C Table in S1 Table. When the assumptions of the parametric beta-binomial models were violated (*e.g.,* dispersion or quantile deviations as assessed by DHARMa), we instead performed non-parametric Kruskal-Wallis and pairwise Wilcoxon rank-sum tests as described below.

Variable selection was guided by a focus on the primary biological variable of interest while avoiding model overspecification and multicollinearity, given the small sample size and unbalanced group structure in terms of potential confounding factors. Our primary analyses therefore focused on the association between SCR, HBB genotype, parasite stages, parasitemia and reticulocyte proportions.

For each analysis, we considered additional demographic and clinical variables as potential confounders. Univariate risk analysis of the *iv*SCA data suggested possible associations between SCR and other variables (S4 Table). Originally correcting for confounding factors was not part of the experimental design and sample size calculations which resulted in low number of observations per level and sparse and uneven representation in the *iv*SCA. Consequently we were unable to properly control for those variables in the GL(M)M analyses (or without violating modelling assumptions in the case of *ev*SCA), Moreover, likelihood ratio tests did not indicate improved model fit when additional variables were included, compared to models using HBB genotype as the sole independent variable. We therefore focused on a descriptive modelling approach and prioritized parsimonious models with a single parameter of interest, which provided the most interpretable inference given the constraints posed by the available data.

Although this approach cannot fully exclude confounding or interaction effects, the direction and magnitude of estimated effects were broadly consistent across alternative model specifications. Sample size and power limitations should still be kept in mind when interpreting some of these GL(M)M results, with emphasis on effect sizes over exact p-values.

## Supporting information

S1 Appendix Assay optimization and validation of *ex vivo* and *in vitro* SC assays, and IgG/IgM detection by flow cytometry. (DOCX)

S2 Appendix Whole genome sequencing analysis of parasite DNA. (DOCX)

S1 Fig Flow chart of participant selection in the *ev*SCAs and *iv*SCAs. Infection status determined by RDT, LM, and *var*ATS qPCR. Samples meeting parasitemia and RBC volume criteria were included in respective assays.
(PDF)

S2 Fig Correlation analysis between parasitemia and SCRs. (A) *ev*SCAs and (B) *iv*SCAs. Data points are colored by HBB genotype. Spearman’s rank correlation was used; non-significant correlations are indicated as ns.
(PDF)

S3 Fig Representative flow cytometry plots showing tdTomato and Hoechst33342 signals. (A) HbAA fresh blood controls cultured under +Choline and -Choline conditions, with and without Hoechst33342. (B-C) Parasites cultured in cryopreserved RBCs with different HBB genotypes under +Choline (B) or -Choline (C) conditions.
(PDF)

S4 Fig Effect of HBB genotype on SC in *iv*SCAs. Related to Fig 2. SCRs were measured by flow cytometry at 25–30 hpi in +Choline and -Choline cultures (A-B). (C-D) Show the proportions of asexual versus sexual stages and the correlation between reticulocyte percentage and SCRs. Statistical tests: (A) Kruskal-Wallis test; (B) beta-binomial GLMs with Bonferroni correction; (E) Spearman correlation.
(PDF)

S5 Fig Experimental variability of SCR measurements in +Choline cultures, grouped by HBB genotype. Timepoint of measurement: at 35–40 hpi (Generation 1).
(PDF)

S6 Fig Flow cytometry detection of IgG and IgM responses against *P. falciparum*-iRBCs. (A) Plasma titration curves with positive and negative controls. (B-C) Gating strategy for measuring antibody reactivity to asexual (tdTom-) and sexual (tdTom+) iRBCs and verification of RBC-only populations using Glycophorin C.
(PDF)

S7 Fig Flow chart illustrating the complete workflow from the flow cytometry-derived parameters to the developmental classification reported in S6 Table. Figure in point 1 (Flow-cytometry derived data) is adapted from Fig 2A, created in BioRender. Yasmina, D. (2026) https://BioRender.com/ew8vbur. Definitions: Reference, all HbAA cultures (HbAA [*n* = 5] and HbAA CTRL [*n* = 6]).
(PDF)

S8 Fig IgG- and IgM-mediated responses to *Plasmodium falciparum* trophozoites and stage I gametocytes across HBB genotypes. (A-D) Event counts and MFI for IgG- and IgM-positive total, asexual, and sexual iRBCs in plasma from *P. falciparum*-infected and non-infected individuals. Statistical comparisons were performed with beta-binomial GLMs with Bonferroni correction.
(PDF)

S9 Fig IgG- and IgM-positive total iRBCs in plasma from *P. falciparum*-infected and non-infected individuals. Median proportions of IgG-positive (A) or IgM-positive (B) total-iRBCs for *Pf*-infected versus non-infected groups. Statistical comparisons were performed with beta-binomial GLMs with Bonferroni correction.
(PDF)

S1 Table S1A Table. Statistical analysis outcomes from GLMs or GLMMs and Kruskal-Wallis tests in *ev*SCAs. S1B Table. Statistical analysis outcomes from GLMs or GLMMs and Kruskal-Wallis test in the *iv*SCAs. S1C Table. Statistical analysis outcomes from GLMs or GLMMs and Kruskal-Wallis tests in the IgG/IgM detection assays.
(PDF)

S2 Table Risk analysis of SCRs and demographic/clinical variables across HBB genotypes in *ev*SCAs. Data from *P. falciparum*-infected samples (parasitemia ≥ 0.05% or 2500 parasites/µl) collected in the Nanoro Department, Burkina Faso. Statistical tests: Chi Square/Fisher exact test, Kruskal Wallis test.
(PDF)

S3 Table Median SCRs (IQR) by HBB genotype measured in *iv*SCAs with flow cytometry. Timepoints measured: at 25–30 hpi and 35–40 hpi (Generation 1).
(PDF)

S4 Table Risk analysis of factors across HBB genotype groups, in *iv*SCAs. (PDF)

S5 Table RBC counts per µl analyzed during flow cytometry to exclude rule hemolysis of fragile mutant RBCs. Measurements were done at three timepoints: 35–40 hpi (Generation 0), and 25–30 hpi and 35–40 hpi (Generation1), for HbAC; HbAS, HbSS and HbSC RBCs. Statistical test: Kruskal-Wallis.
(PDF)

S6 Table Analysis of parasite stage development by individual parasite culture within each HBB genotype group. Parameters (PMR, late-stage proportions, fold changes) compared to HbAA reference and color-coded. Cultures classified as expected, delayed, or (partially) arrested/extremely delayed. SCR at 35–40 hpi (Generation 1) also shown.
(PDF)

S7 Table Median IgG- and IgM-positive proportions of total, asexual, and sexual iRBCs by HBB genotype. Measurements done by flow cytometry.
(PDF)

S8 Table Demographics of participants in IgG/IgM detection assays across HBB genotype groups. Data was analyzed in *P. falciparum* infected and non-infected plasma samples collected in the Nanoro Department, Burkina Faso within the context of the *InHost* study.
(PDF)

S9 Table Overview *Pfsa* genotypes detected in samples used in *ev*SCA grouped by HBB genotype: count, mean *ev*SCR and mean parasitemia (D0). (PDF)

S10 Table Key resources, primers, and probe sequences. (DOCX)

S11 Table Number of samples (n) per *Plasmodium falciparum* infection status and donor HBB genotype at collection. (DOCX)

S12 Table Overview of experiments and number of cryopreserved RBC samples by HBB genotype in *iv*SCAs. (DOCX)
