# Supplementary material for: Elevated Plasmodium falciparum sexual conversion in HbAC and HbAS red blood cells in naturally infected malaria patients
Source: PLoS Pathog. 2026 Sep 29;22(9):e1014542. doi: 10.1371/journal.ppat.1014542 (PMC13623122; doi:10.1371/journal.ppat.1014542)
Supplement: Supplementary file 1 [file ppat.1014542.s003.pdf]

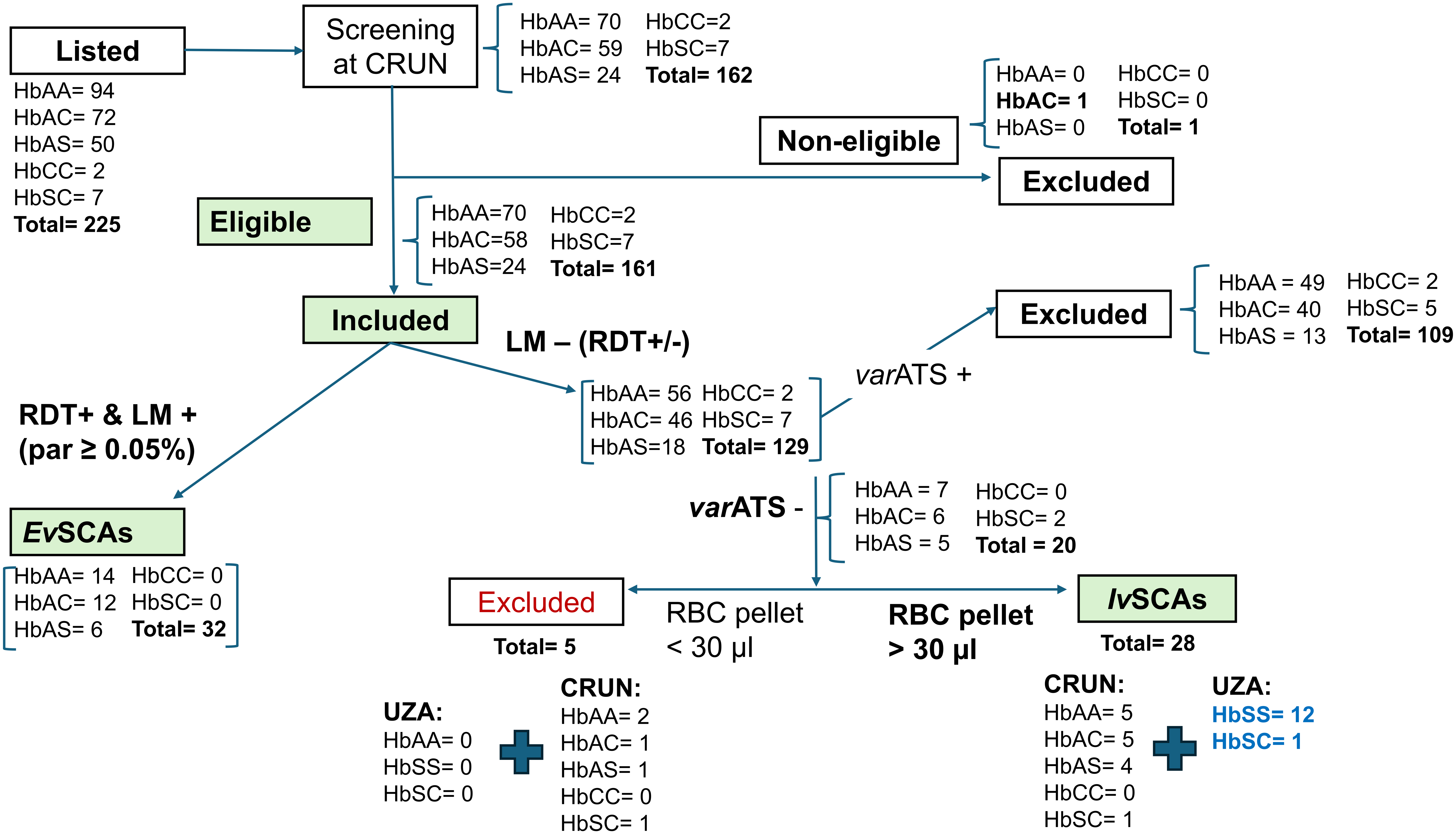

Listed
HbAA= 94
HbAC= 72
HbAS= 50
HbCC= 2
HbSC= 7
Total= 225
Screening at CRUN
HbAA= 70 HbCC=2
HbAC= 59 HbSC=7
HbAS= 24 Total= 162
Non-eligible
HbAA= 0 HbCC= 0
HbAC= 1 HbSC= 0
HbAS= 0 Total= 1
Excluded
Eligible
HbAA=70 HbCC=2
HbAC=58 HbSC=7
HbAS=24 Total= 161
Included
LM – (RDT+/-)
Excluded
HbAA = 49 HbCC= 2
HbAC= 40 HbSC= 5
HbAS = 13 Total= 109
varATS +
HbAA= 56 HbCC= 2
HbAC= 46 HbSC= 7
HbAS=18 Total= 129
RDT+ & LM +
(par ≥ 0.05%)
varATS -
HbAA = 7 HbCC= 0
HbAC= 6 HbSC= 2
HbAS = 5 Total = 20
EvSCAs
HbAA= 14 HbCC= 0
HbAC= 12 HbSC= 0
HbAS= 6 Total= 32
Excluded
Total= 5
RBC pellet
< 30 µl
RBC pellet
> 30 µl
IvSCAs
Total= 28
UZA:
HbAA= 0
HbSS= 0
HbSC= 0
CRUN:
HbAA= 2
HbAC= 1
HbAS= 1
HbCC= 0
HbSC= 1
CRUN:
HbAA= 5
HbAC= 5
HbAS= 4
HbCC= 0
HbSC= 1
UZA:
HbSS= 12
HbSC= 1
