# Supplementary material for: Elevated Plasmodium falciparum sexual conversion in HbAC and HbAS red blood cells in naturally infected malaria patients
Source: PLoS Pathog. 2026 Sep 29;22(9):e1014542. doi: 10.1371/journal.ppat.1014542 (PMC13623122; doi:10.1371/journal.ppat.1014542)
Supplement: Supplementary file 2 [file ppat.1014542.s004.pdf]

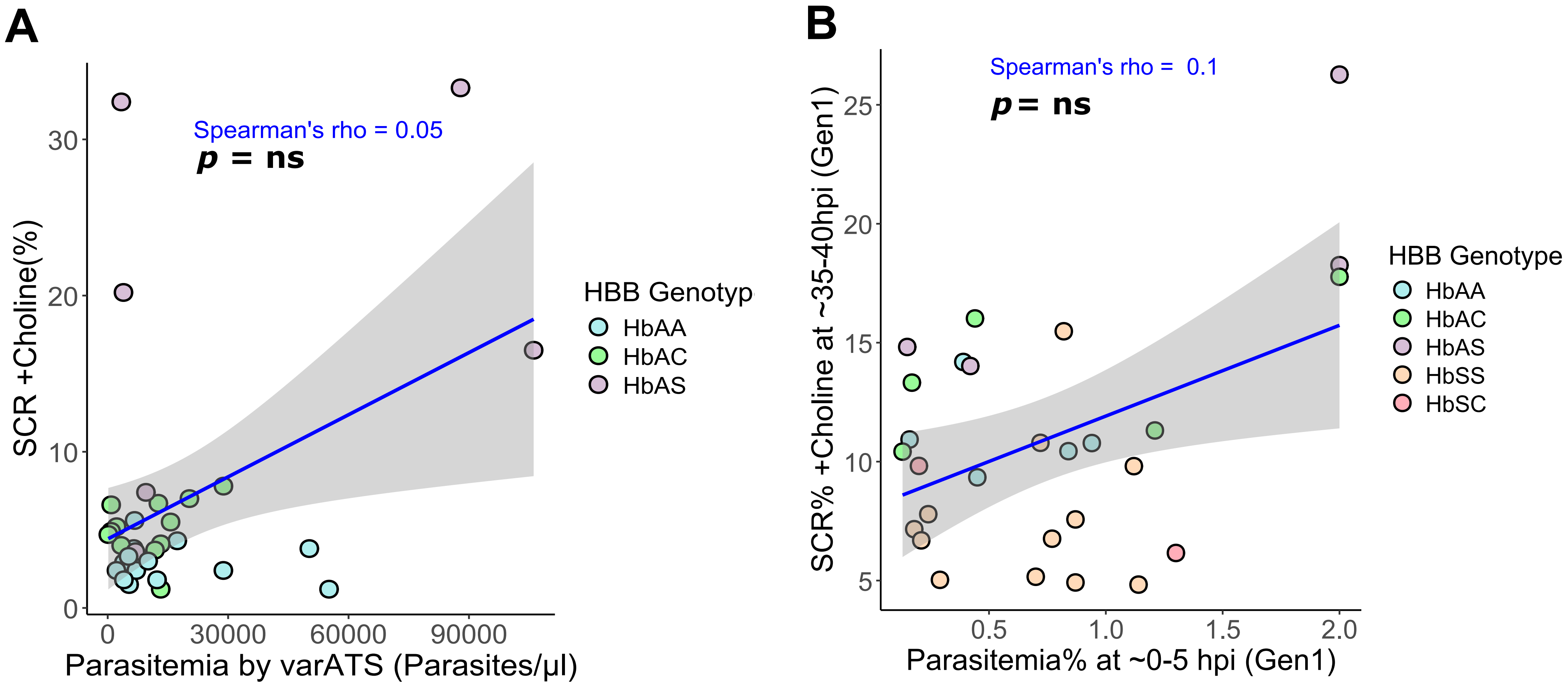

A
Spearman's rho = 0.05
p = ns
SCR +Choline(%)
0
10
20
30
0
30000
60000
90000
Parasitemia by varATS (Parasites/µl)
HBB Genotyp
HbAA
HbAC
HbAS
B
Spearman's rho = 0.1
p = ns
SCR% +Choline at ~35-40hpi (Gen1)
5
10
15
20
25
0.5
1.0
1.5
2.0
Parasitemia% at ~0-5 hpi (Gen1)
HBB Genotype
HbAA
HbAC
HbAS
HbSS
HbSC
