# Supplementary material for: Elevated Plasmodium falciparum sexual conversion in HbAC and HbAS red blood cells in naturally infected malaria patients
Source: PLoS Pathog. 2026 Sep 29;22(9):e1014542. doi: 10.1371/journal.ppat.1014542 (PMC13623122; doi:10.1371/journal.ppat.1014542)
Supplement: Supplementary file 3 [file ppat.1014542.s005.pdf]

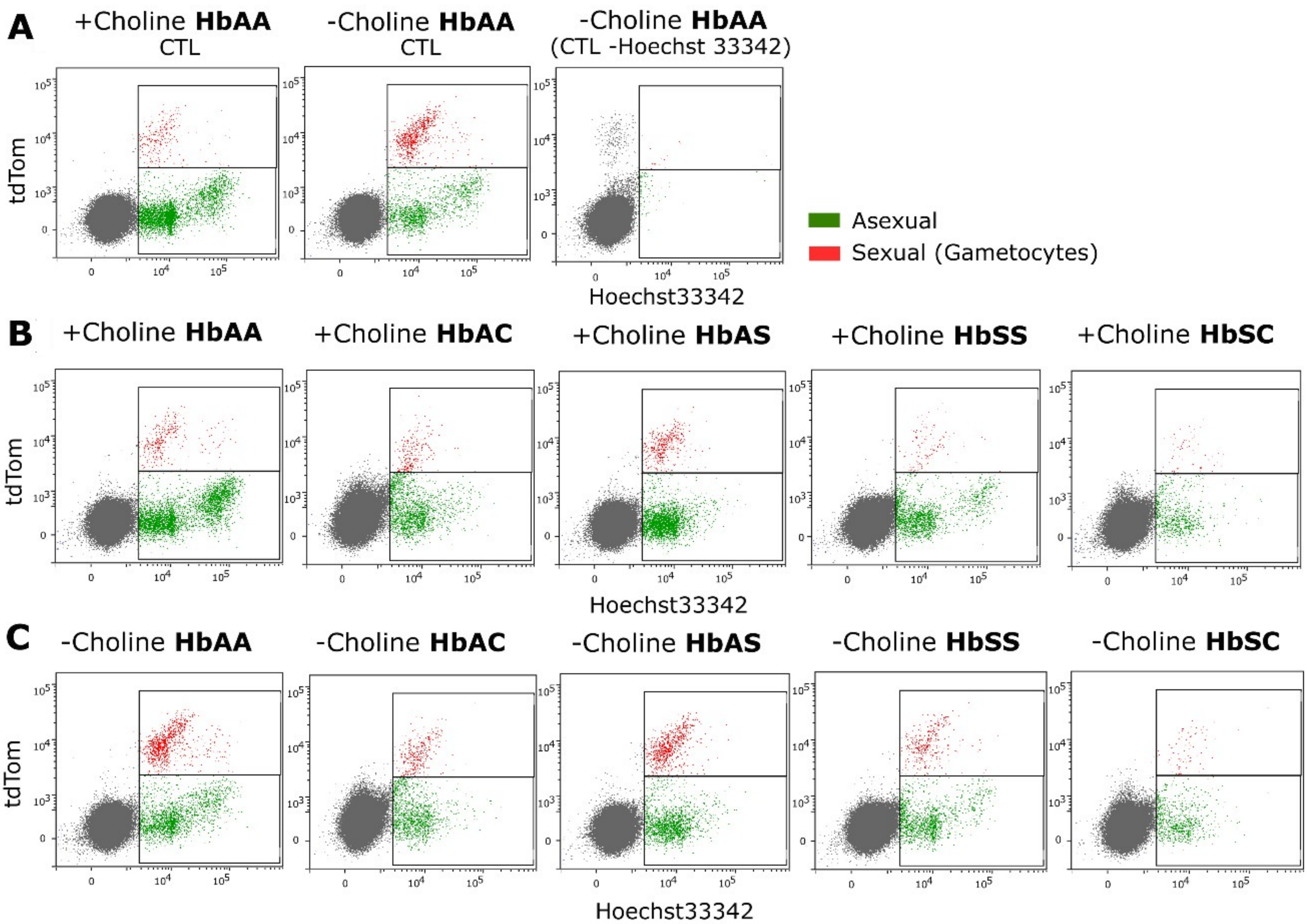
A
+Choline HbAA CTL
-Choline HbAA CTL
-Choline HbAA (CTL -Hoechst 33342)
tdTom
Hoechst33342
Asexual
Sexual (Gametocytes)
B
+Choline HbAA
+Choline HbAC
+Choline HbAS
+Choline HbSS
+Choline HbSC
C
-Choline HbAA
-Choline HbAC
-Choline HbAS
-Choline HbSS
-Choline HbSC
