# Supplementary material for: Elevated Plasmodium falciparum sexual conversion in HbAC and HbAS red blood cells in naturally infected malaria patients
Source: PLoS Pathog. 2026 Sep 29;22(9):e1014542. doi: 10.1371/journal.ppat.1014542 (PMC13623122; doi:10.1371/journal.ppat.1014542)
Supplement: Supplementary file 4 [file ppat.1014542.s006.pdf]

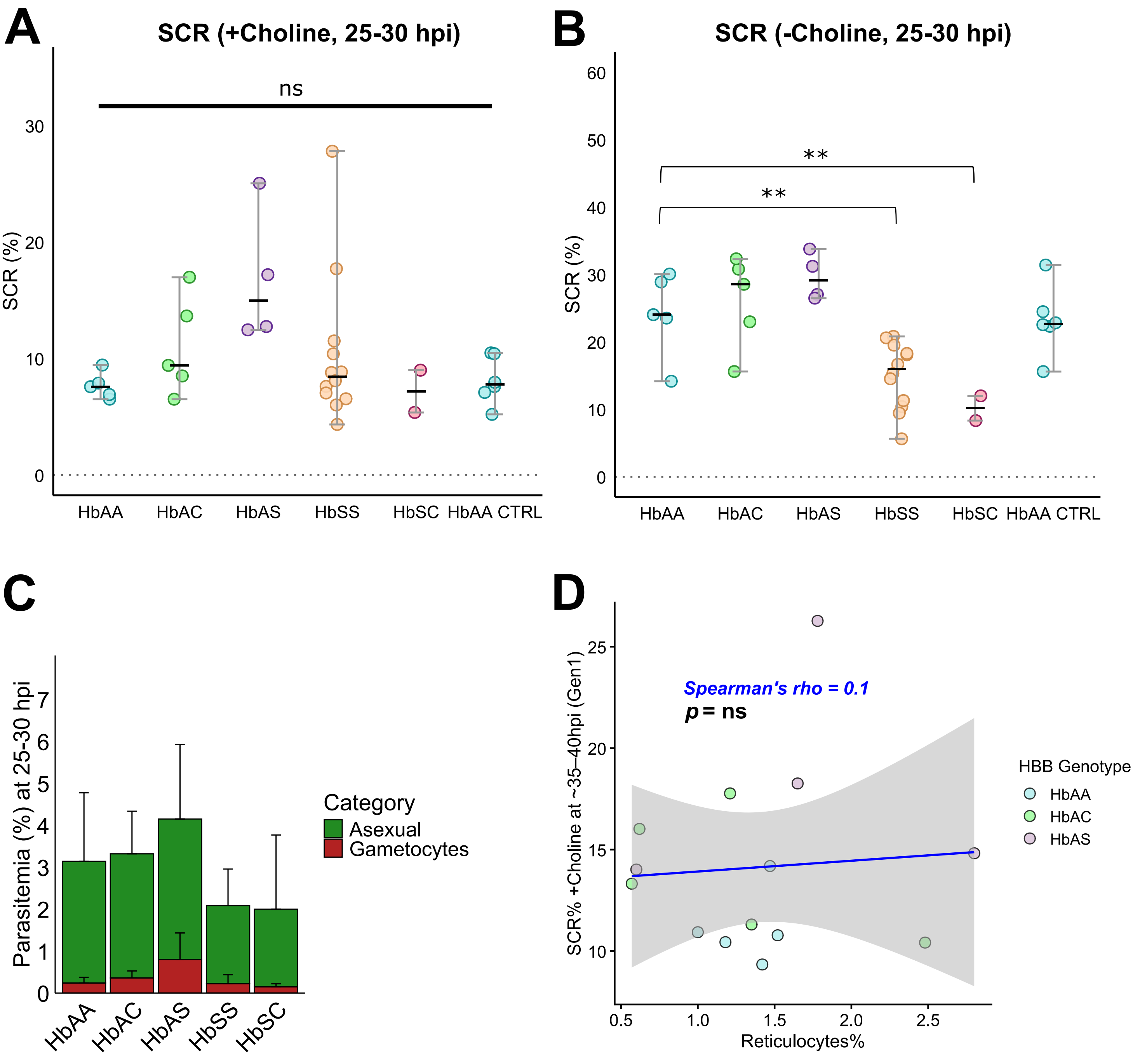

A
SCR (+Choline, 25-30 hpi)
ns
SCR (%)
0
10
20
30
HbAA
HbAC
HbAS
HbSS
HbSC
HbAA CTRL
B
SCR (-Choline, 25-30 hpi)
**
**
SCR (%)
0
10
20
30
40
50
60
HbAA
HbAC
HbAS
HbSS
HbSC
HbAA CTRL
C
Parasitemia (%) at 25-30 hpi
0
1
2
3
4
5
6
7
HbAA
HbAC
HbAS
HbSS
HbSC
Category
Asexual
Gametocytes
D
Spearman's rho = 0.1
p = ns
SCR% +Choline at ~35–40hpi (Gen1)
10
15
20
25
0.5
1.0
1.5
2.0
2.5
Reticulocytes%
HBB Genotype
HbAA
HbAC
HbAS
