# Supplementary material for: Elevated Plasmodium falciparum sexual conversion in HbAC and HbAS red blood cells in naturally infected malaria patients
Source: PLoS Pathog. 2026 Sep 29;22(9):e1014542. doi: 10.1371/journal.ppat.1014542 (PMC13623122; doi:10.1371/journal.ppat.1014542)
Supplement: Supplementary file 5 [file ppat.1014542.s007.pdf]

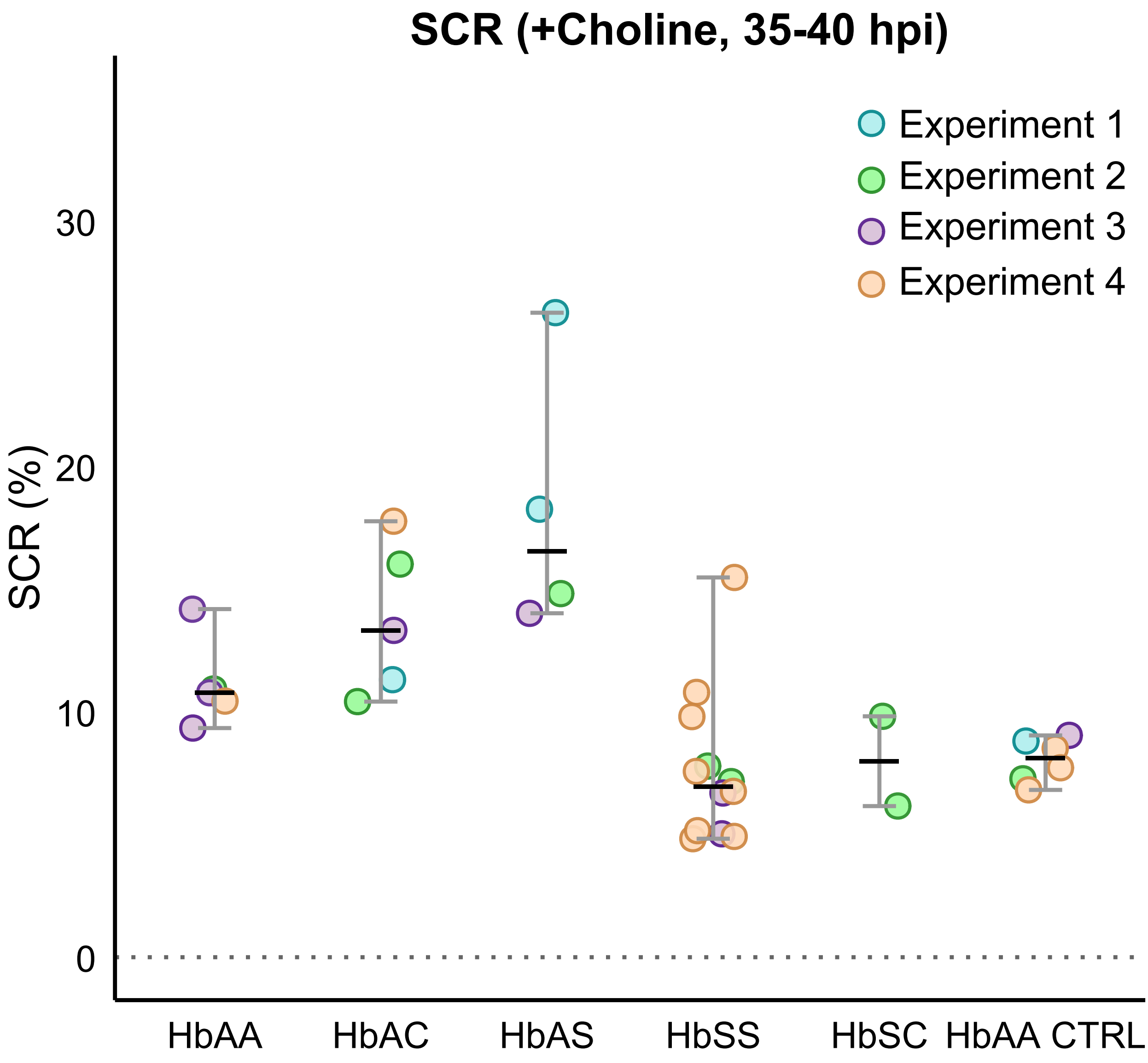

SCR (+Choline, 35-40 hpi)
Experiment 1
Experiment 2
Experiment 3
Experiment 4
SCR (%)
30
20
10
0
HbAA
HbAC
HbAS
HbSS
HbSC
HbAA CTRL
