# Supplementary material for: Elevated Plasmodium falciparum sexual conversion in HbAC and HbAS red blood cells in naturally infected malaria patients
Source: PLoS Pathog. 2026 Sep 29;22(9):e1014542. doi: 10.1371/journal.ppat.1014542 (PMC13623122; doi:10.1371/journal.ppat.1014542)
Supplement: Supplementary file 6 [file ppat.1014542.s008.pdf]

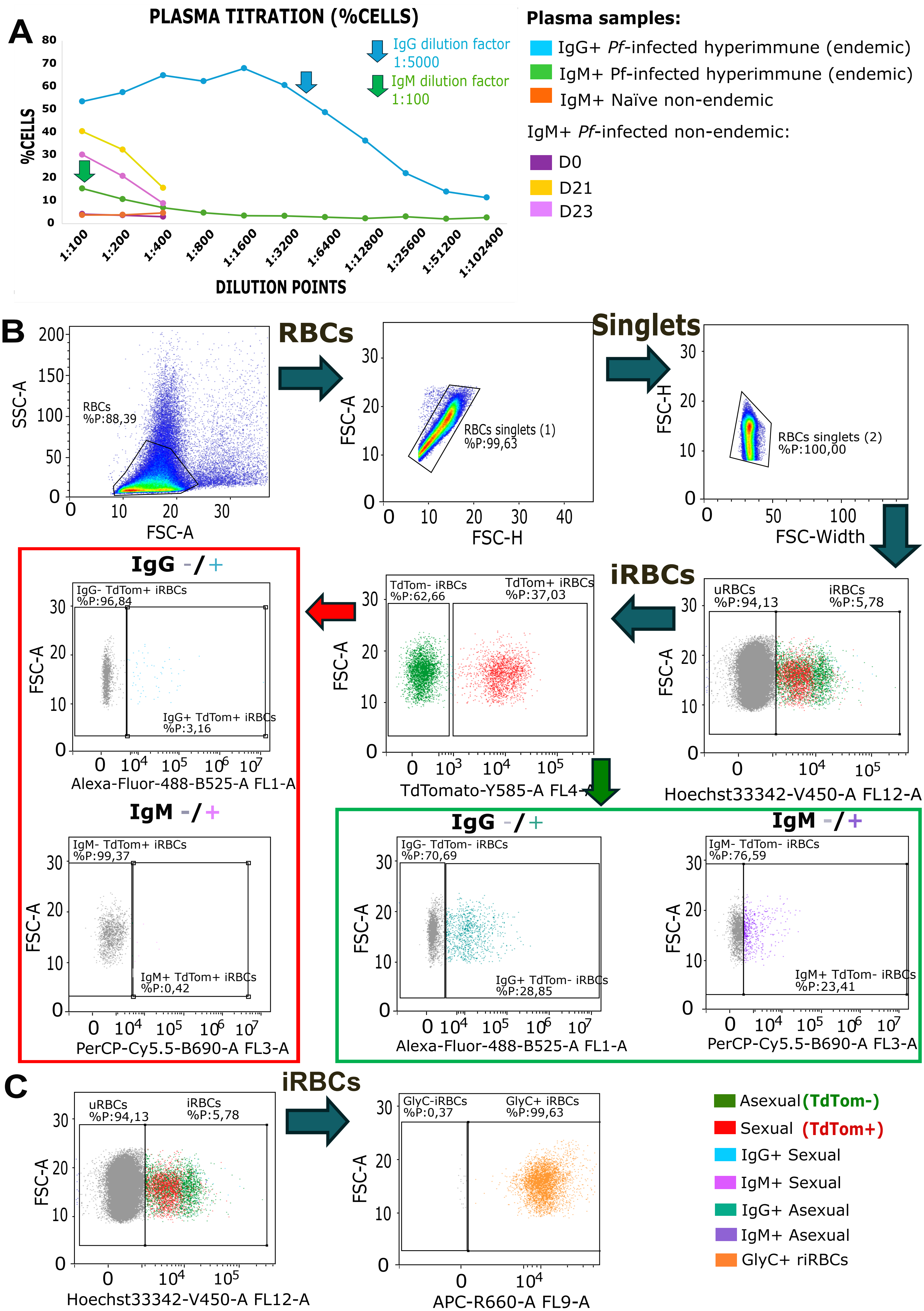

A
PLASMA TITRATION (%CELLS)
%CELLS
DILUTION POINTS
IgG dilution factor 1:5000
IgM dilution factor 1:100
Plasma samples:
IgG+ Pf-infected hyperimmune (endemic)
IgM+ Pf-infected hyperimmune (endemic)
IgM+ Naïve non-endemic
IgM+ Pf-infected non-endemic:
D0
D21
D23
B
RBCs
RBCs %P:88,39
SSC-A
FSC-A
Singlets
RBCs singlets (1) %P:99,63
FSC-H
RBCs singlets (2) %P:100,00
FSC-Width
iRBCs
uRBCs %P:94,13
iRBCs %P:5,78
Hoechst33342-V450-A FL12-A
TdTom- iRBCs %P:62,66
TdTom+ iRBCs %P:37,03
TdTomato-Y585-A FL4-A
IgG -/+
IgG- TdTom+ iRBCs %P:96,84
IgG+ TdTom+ iRBCs %P:3,16
Alexa-Fluor-488-B525-A FL1-A
IgM -/+
IgM- TdTom+ iRBCs %P:99,37
IgM+ TdTom+ iRBCs %P:0,42
PerCP-Cy5.5-B690-A FL3-A
IgG -/+
IgG- TdTom- iRBCs %P:70,69
IgG+ TdTom- iRBCs %P:28,85
Alexa-Fluor-488-B525-A FL1-A
IgM -/+
IgM- TdTom- iRBCs %P:76,59
IgM+ TdTom- iRBCs %P:23,41
PerCP-Cy5.5-B690-A FL3-A
C
uRBCs %P:94,13
iRBCs %P:5,78
Hoechst33342-V450-A FL12-A
iRBCs
GlyC-iRBCs %P:0,37
GlyC+ iRBCs %P:99,63
APC-R660-A FL9-A
Asexual (TdTom-)
Sexual (TdTom+)
IgG+ Sexual
IgM+ Sexual
IgG+ Asexual
IgM+ Asexual
GlyC+ riRBCs
