# Supplementary material for: Elevated Plasmodium falciparum sexual conversion in HbAC and HbAS red blood cells in naturally infected malaria patients
Source: PLoS Pathog. 2026 Sep 29;22(9):e1014542. doi: 10.1371/journal.ppat.1014542 (PMC13623122; doi:10.1371/journal.ppat.1014542)
Supplement: Supplementary file 7 [file ppat.1014542.s009.pdf]

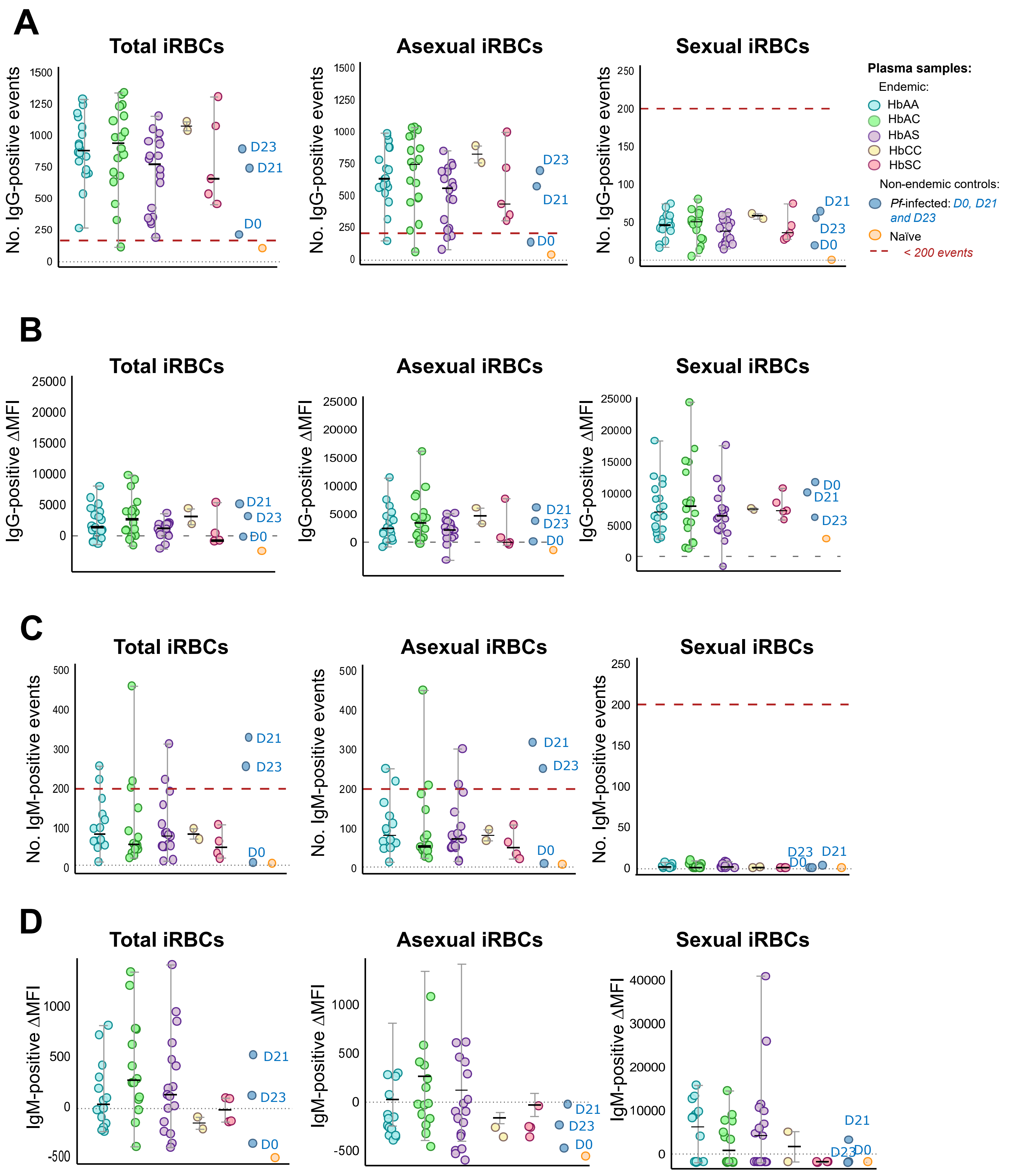
A
Total iRBCs
Asexual iRBCs
Sexual iRBCs
No. IgG-positive events
Plasma samples:
Endemic:
HbAA
HbAC
HbAS
HbCC
HbSC
Non-endemic controls:
Pf-infected: D0, D21 and D23
Naïve
< 200 events
D23
D21
D0
B
IgG-positive ΔMFI
C
No. IgM-positive events
D
IgM-positive ΔMFI
