# Supplementary material for: Elevated Plasmodium falciparum sexual conversion in HbAC and HbAS red blood cells in naturally infected malaria patients
Source: PLoS Pathog. 2026 Sep 29;22(9):e1014542. doi: 10.1371/journal.ppat.1014542 (PMC13623122; doi:10.1371/journal.ppat.1014542)
Supplement: Supplementary file 8 [file ppat.1014542.s010.pdf]

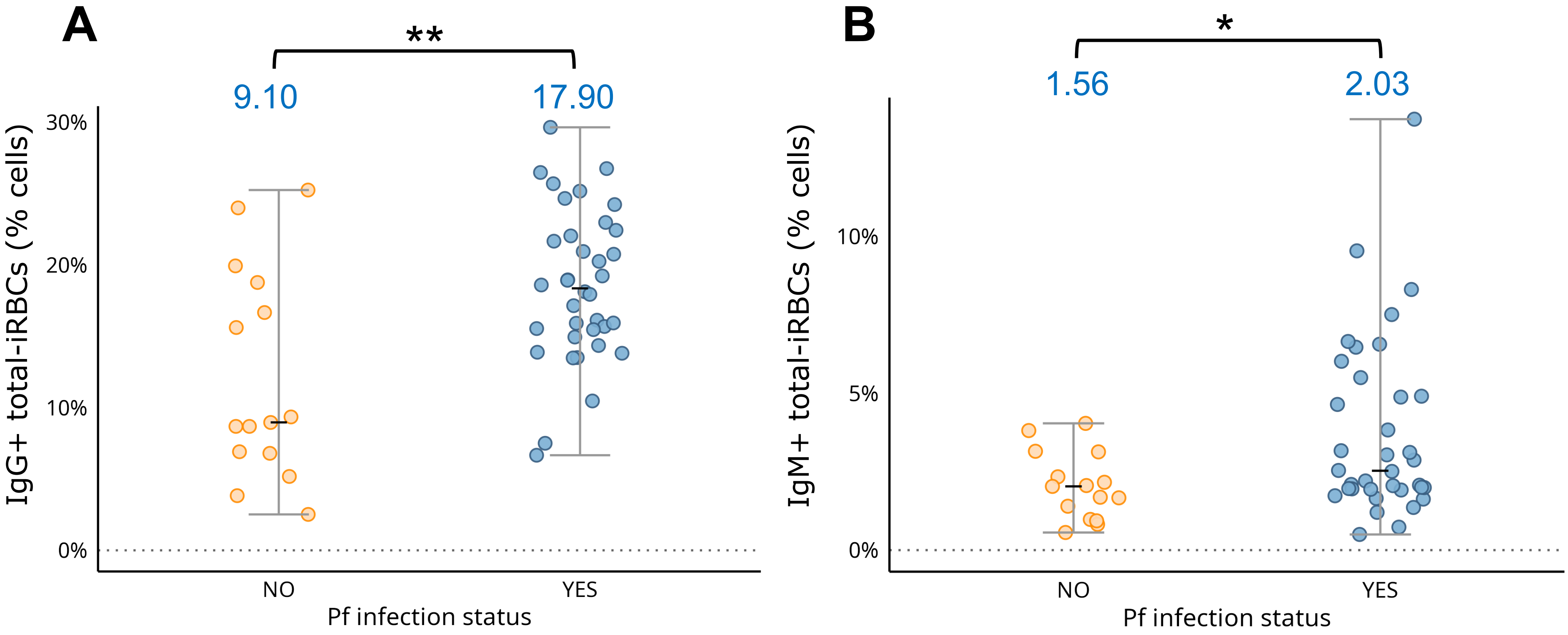
A
**
9.10
17.90
30%
20%
10%
0%
IgG+ total-iRBCs (% cells)
NO
YES
Pf infection status
B
*
1.56
2.03
10%
5%
0%
IgM+ total-iRBCs (% cells)
NO
YES
Pf infection status
