# Supplementary material for: Elevated Plasmodium falciparum sexual conversion in HbAC and HbAS red blood cells in naturally infected malaria patients
Source: PLoS Pathog. 2026 Sep 29;22(9):e1014542. doi: 10.1371/journal.ppat.1014542 (PMC13623122; doi:10.1371/journal.ppat.1014542)
Supplement: Supplementary file 10 [file ppat.1014542.s012.pdf]

S1A Table. Statistical analysis outcomes from GLMs or GLMMs and Kruskal-Wallis tests in the *ev*SCAs.

Model definition	Model Type	Estimates (transformed to probability scale / estimated probability	Contrasts	OR [95% CI] (Wald Z tests)	(Adjusted) *p*-value	Bayesian OR [credible interval]	Comments
**SCR+Choline ~ HBB genotype + (1|Sample)**<br><br>***Φ ~ HBB genotype***	**Beta-binomial GLMM**	AA = 0.027<br>AC = 0.048<br>AS = 0.186	HbAC vs. HbAA	1.82 [1.17−2.82]	0.0032	-	• Technical replicates modelled as random effect.<br>• Genotype-specific dispersion was modelled.<br>• Bayesian model failed to converge.<br>• Bonferroni correction for pairwise comparisons
			HbAS vs. HbAA	8.16 [4.34−15.37]	< 0.0001	-	

Abbreviations: CI, confidence interval; *ev*SCAs, *ex vivo* sexual conversion assays; GLMs or GLMMs, generalized linear (mixed) models; Hb, hemoglobin; HBB, human hemoglobin beta; iRBCs, infected red blood cells; OR, odds ratio; SC, sexual conversion; SCR, sexual conversion rate; Sexual conversion rates (SCRs) are the proportion of *Plasmodium falciparum* parasites that commit to sexual development relative to total parasitemia.

S1B Table. **Statistical analysis outcomes from GLMs or GLMM and Kruskal-Wallis test in the *iv*SCAs.**

Model definition	Model Type	Median[IQR]	Estimates (transformed to probability scale / estimated probability	Contrasts	OR [95% CI] (Wals Z tests)	(Adjusted) *p*-value	Bayesian OR [credible interval]	Comments
**SCR+Choline (Gen 1, 35-40hpi) ~ HBB genotype**	**Beta-binomial GLM**	**-**	AA = 0.114<br>AC = 0.138<br>AS = 0.182<br>SS = 0.076<br>SC = 0.081	HbAC vs. HbAA	1.25 [0.81−1.92]	0.77	1.25 [0.80−1.85]	• Bonferroni correction for pairwise comparisons
				HbAS vs. HbAA	1.73 [1.12−2.66]	0.0056	1.73 [1.09−2.52]	
				HbSS vs. HbAA	0.64 [0.43−0.95]	0.021	0.65 [0.43−0.92]	
				HbSC vs. HbAA	0.68 [0.35−1.33]	0.62	0.67 [0.28–1.15]	
**SCR+Choline (Gen 1, 25-30hpi) ~ HBB genotype**	**Kruskal-Wallis test**	AA= 7.58[0.98)<br>AC= 9.41[5.14]<br>AS= 15.00[6.48]<br>SS= 8.44 [3.76]<br>SC= 7.18 [1.81]	-	-	-	0.094	-	• Beta-binomial model assumptions violated (quantile deviations detected).
				-	-		-	
				-	-		-	
				-	-		-	
**SCR-Choline (Gen 1, 35-40hpi) ~ HBB genotype**	**Beta-binomial GLM**	**-**	AA = 0.321<br>AC = 0.326<br>AS = 0.333<br>SS = 0.162<br>SC = 0.124	HbAC vs. HbAA	1.02 [0.66−1.59]	1	1.02 [0.65-1.48]	• Bonferroni correction for pairwise comparisons
				HbAS vs. HbAA	1.06 [0.66−1.69]	1	1.06 [0.65-1.56]	
				HbSS vs. HbAA	0.41 [0.27−0.61]	< 0.0001	0.41 [0.27-0.58]	
				HbSC vs. HbAA	0.30 [0.14−0.64]	0.0003	0.29 [0.10-0.52]	
**SCR-Choline (Gen 1, 25-30hpi) ~ HBB genotype**	**Beta-binomial GLM**	**-**	AA = 0.250<br>AC = 0.255<br>AS = 0.299<br>SS = 0.149<br>SC = 0.106	HbAC vs. HbAA	1.03 [0.65−1.63]	1	1.04 [0.65-1.55]	• Bonferroni correction for pairwise comparisons
				HbAS vs. HbAA	1.28 [0.81−2.03]	0.73	1.29 [0.81-1.93]	
				HbSS vs. HbAA	0.52 [0.35−0.79]	0.0003	0.54 [0.35-0.76]	
				HbSC vs. HbAA	0.36 [0.17−0.77]	0.0032	0.35 [0.13-0.65]	
**Late stages proportion (Gen 0, 35-40hpi) ~ HBB genotype**	**Kruskal-Wallis test**	AA= 45.00[20.00]<br>AC= 58.00[10.00]<br>AS=26.00[18.20]<br>SS=9.00[16.50]<br>SC= 8.00[6.00]	-	-	-	0.05	-	• Beta-binomial model assumptions violated (dispersion and quantile deviations).<br>• *Not measured for some samples due to limited RBCs; SCR measured for all samples.*
				-	-		-	
				-	-		-	
				-	-		-	
**Late stages proportion (Gen 1, 25-30hpi) ~ HBB genotype**	**Kruskal-Wallis test**	AA= 2.00[3.00]<br>AC= 4.00[1.00]<br>AS=3.50[3.50]<br>SS= 2.50[3.25]<br>SC=4.00[1.00]	-	-	-	0.13	-	• Beta-binomial model assumptions violated (quantile deviations).
				-	-		-	
				-	-		-	
				-	-		-	
**Late stages proportion (Gen 1, 35-**	**Beta-binomial GLM**	-	AA = -1.45<br>AC = -0.21<br>AS = -0.52	HbAC vs. HbAA	0.81 [0.24−2.76]	1	0.81 [0.17-2.03]	• Bonferroni correction for pairwise comparisons
				HbAS vs. HbAA	0.60 [0.15−2.31]	1	0.59 [0.09-1.61]	
				HbSS vs. HbAA	0.21 [0.06−0.66]	0.0026	0.22 [0.06-0.55]	

**40hpi) ~ HBB genotype**			SS = -1.57<br>SC = -0.63	HbSC vs. HbAA	0.53 [0.09−3.02]	1	0.49 [0.01-1.64]	
**Parasitemia (Gen 0, 0-5hpi) ~HBB genotype**	**Beta-binomial GLM**	-	AA = -4.99<br>AC = 0.16<br>AS = 0.026<br>SS = 0.11<br>SC = 0.024	HbAC vs. HbAA	1.18 [0.40−3.48]	1	1.17 [0.29-2.72]	• Bonferroni correction for pairwise comparisons
				HbAS vs. HbAA	1.03 [0.31−3.37]	1	1.01 [0.19-2.49]	
				HbSS vs. HbAA	1.12 [0.45−2.82]	1	1.15 [0.42-2.49]	
				HbSC vs. HbAA	1.02 [0.24−4.41]	1	0.93 [0.04-2.60]	
**Reticulocytes ~HBB genotype**	**Beta-binomial GLM**	-	AA = -4.13<br>AC = -0.14<br>AS = 0.11<br>SS = 0.94<br>SC = 0.85	HbAC vs. HbAA	0.86 [0.32−2.36]	1	0.87 [0.27−1.94]	• Bonferroni correction for pairwise comparisons
				HbAS vs. HbAA	1.13 [0.41−3.07]	1	1.11[0.28−2.44]	
				HbSS vs. HbAA	2.56 [1.20−5.44]	0.008	2.54 [1.11−4.87]	
				HbSC vs. HbAA	2.33 [0.82−6.64]	0.18	2.19 [0.44−4.92]	

Abbreviations: CI, confidence interval; GLMs or GLMMs, Generalized Linear (Mixed) Models; Hb, hemoglobin; HBB, human hemoglobin beta; iRBCs, infected red blood cells; IQR, Interquartile range; *iv*SCAs, *in vitro* sexual conversion assays; OR, Odds Ratio; SC, sexual conversion; SCR, sexual conversion rate; Sexual conversion rates (SCRs) are the proportion of *Plasmodium falciparum* parasites that commit to sexual development on the total parasitemia.

S1C Table . **Statistical analysis outcomes from GLMs or GLMMs and Kruskal-Wallis tests in the IgG/IgM detection assays.**

Model definition	Model Type	Estimates	Contrasts	OR [95% CI] (Wald Z tests)	(Adjusted) *p*-value$	Bayesian OR [credible interval]	Comments
**IgG+ total iRBCs (%cells) ~ HBB genotype**	**Beta-binomial GLM**	AA = -1.52<br>AC = -0.10<br>AS = -0.23<br>CC = 0.25<br>SC = -0.031	HbAC vs. HbAA	0.90 [0.57−1.43]	1	0.90 [0.59−1.30]<br>0.78 [0.50−1.10]<br>1.23 [0.42−2.27]<br>0.96 [0.47−1.60]	• Bonferroni correction for pairwise comparisons
			HbAS vs. HbAA	0.78 [0.50−1.22]	0.67		
			HbCC vs. HbAA	1.28 [0.54−3.01]	1		
			HbSC vs. HbAA	0.97 [0.49−1.92]	1		
**IgG+ asexual iRBCs (%cells) ~ HBB genotype**	**Beta-binomial GLM**	AA = -1.28<br>AC = -0.091<br>AS = -0.30<br>CC = 0.29<br>SC = -0.10	HbAC vs. HbAA	0.91 [0.53−1.56]	1	0.92 [0.55−1.37]<br>0.74 [0.46−1.10]<br>1.30 [0.34−2.71]<br>0.89 [0.37−1.59]	• Bonferroni correction for pairwise comparisons
			HbAS vs. HbAA	0.74 [0.44−1.25]	0.61		
			HbCC vs. HbAA	1.34 [0.49−3.68]	1		
			HbSC vs. HbAA	0.91 [0.40−2.04]	1		
**IgG+ sexual iRBCs (%cells) ~ HBB genotype**	**Beta-binomial GLM**	AA = -3.70<br>AC = -0.11<br>AS = -0.15<br>CC = 0.27<br>SC = 0.030	HbAC vs. HbAA	0.89 [0.59−1.36]	1	0.89 [0.59−1.24]<br>0.86 [0.60−1.20]<br>1.25 [0.49−2.14]<br>1.01 [0.52−1.59]	• Bonferroni correction for pairwise comparisons
			HbAS vs. HbAA	0.86 [0.58−1.29]	1		
			HbCC vs. HbAA	1.31 [0.63−2.73]	1		
			HbSC vs. HbAA	1.03 [0.56−1.89]	1		
**IgM+ total iRBCs (%cells) ~ HBB genotype**	**Beta-binomial GLM**	AA = -3.33<br>AC = -0.067<br>AS = -0.039<br>CC = -0.067<br>SC = -0.44	HbAC vs. HbAA	0.94 [0.52−1.68]	1	0.94 [0.52−1.44]	• Bonferroni correction for pairwise comparisons
			HbAS vs. HbAA	0.96 [0.55−1.68]	1	0.97 [0.58−1.48]	
			HbCC vs. HbAA	0.94 [0.29−3.03]	1	0.85 [0.13−1.85]	
			HbSC vs. HbAA	065 [0.24−1.72]	1	0.62 [0.17−1.21]	
**IgM+ asexual iRBCs (%cells) ~ HBB genotype**	**Beta-binomial GLM**	AA = -3.35<br>AC = -0.076<br>AS = -0.039<br>CC = -0.069<br>SC = -0.42	HbAC vs. HbAA	0.93 [0.52−1.66]	1	0.93 [0.54−1.45]	• Bonferroni correction for pairwise comparisons
			HbAS vs. HbAA	0.96 [0.55−1.67]	1	0.97 [0.58−1.46]	
			HbCC vs. HbAA	0.93 [0.29−3.01]	1	0.84 [1.10−1.79]	
			HbSC vs. HbAA	0.65 [0.25−1.72]	1	0.64 [0.17−1.20]	
**IgM+ sexual iRBCs (%cells) ~ HBB genotype**	**Beta-binomial GLM**	AA = -7.01e+00<br>AC = 1.41e-01<br>AS = 2.06e-02<br>CC = -4.40e-01<br>SC = -1.97e+01	HbAC vs. HbAA	1.15 [0.34−4.00]	1	1.04 [0.28−2.47]	• Bonferroni correction for pairwise comparisons
			HbAS vs. HbAA	1.02 [0.32−3.00]	1	0.98 [0.26−2.25]	
			HbCC vs. HbAA	0.64 [0.05−9.00]	1	0.52 [0.001−2.44]	
			HbSC vs. HbAA	0.00 [0.00−Inf]	1	0.00 [0.00−0.027]	

**IgG+ total iRBCs (%cells) ~ *Pf*-infection**	**Beta-binomial GLM**	NO = -2.06<br>YES = 0.59	YES vs. NO	1.81 [1.35−2.43]	**0.001**	1.82 [1.31−2.43]	• Bonferroni correction for pairwise comparisons<br>•
**IgM+ total iRBCs (%cells) ~ *Pf*_infection**	**Beta-binomial GLM**	NO = -3.70<br>YES = 0.41	YES vs. NO	1.51 [1.01−2.23]	**0.043**	1.51 [0.96−2.20]	• Bonferroni correction for pairwise comparisons

Abbreviations: Asexual iRBCs, trophozoite-iRBCs; CI, confidence interval; GLMs or GLMMs, Generalized Linear (Mixed) Models; Hb, hemoglobin; HBB, human hemoglobin beta; iRBCs, infected red blood cells; OR, Odds Ratio; *Pf, P. falciparum*; Sexual iRBCs, stage I gametocyte-iRBCs; Total iRBCs, trophozoite- and stage I gametocyte-iRBCs. %cells are the proportion of IgG- or IgM-positive infected red blood cells.
