# Supplementary material for: Elevated Plasmodium falciparum sexual conversion in HbAC and HbAS red blood cells in naturally infected malaria patients
Source: PLoS Pathog. 2026 Sep 29;22(9):e1014542. doi: 10.1371/journal.ppat.1014542 (PMC13623122; doi:10.1371/journal.ppat.1014542)
Supplement: Supplementary file 11 [file ppat.1014542.s013.pdf]

S2 Table. Risk analysis of SCRs and demographic/clinical variables across HBB genotypes in *ev*SCAs.

	HbAA	HbAC	HbAS	*p*-value$
**SCR *n*(%)**	14 (100)	12 (100)	6 (100)	< 0.001
**Gender *n*(%)**				0.734
Female	8 (57.1)	5 (41.7)	3 (50)	
Male	6 (42.9)	7 (58.3)	3 (50)	
**Village *n*(%)**				0.710
Nanoro	2 (14.3)	2 (16.7)	2 (33.3)	
Nazoanga	5 (35.7)	3 (25)	1 (16.7)	
Soum	7 (50)	6 (50)	2 (33.3)	
Séguédin	0 (0)	1 (8.3)	1 (16.7)	
**Ethnicity *n*(%)**				0.231
Gourounsi	1 (7.1)	4 (33.3)	1 (16.7)	
Mossi	13 (92.9)	8 (66.6)	5 (83.3)	
**Symptomatic *n*(%)**				0.351
NO	5 (35.7)	4 (33.3)	4 (66.7)	
YES	9 (62.3)	8 (66.6)	2 (33.3)	
**RDT Positives *n*(%)**	14 (100)	12 (100)	6 (100)	NA
**LM Positives *n*(%)**	14 (100)	12 (100)	6 (100)	NA
***P. falciparum* infections *n*(%)**	14 (100)	12 (100)	6 (100)	NA
**Mixed Infections = None *n*(%)**	14 (100)	12 (100)	6 (100)	NA
**Age, mean (SD)**	9.31 (7.80)	7.58 (1.60)	8.03 (1.66)	0.957
**Axillary Temperature, mean (SD)**	36.54 (0.50)	36.53 (0.43)	36.37 (0.29)	0.747
**LM (par/µl), mean (SD)**	10603.14 (12153.08)	9653.67 (5609.73)	16620.33 (21368.16)	0.820
**LM (gam/µl), mean (SD)**	0 (0)	19.58 (48.74)	13.00 (31.84)	0.288
***VarATS* (par/µl), mean (SD)**	15354.50 (17269.06)	10262.25 (9008.48)	36290.50 (47430.62)	0.750

$Categorical variables test: Chi Square /Fisher exact test. Continuous variables test: Kruskal Wallis test.
Data from *P. falciparum*-infected samples (parasitemia ≥ 0.05% or 2500 parasites/µl) collected in Nanoro, Burkina Faso. Frequencies within each category group are expressed as percentages (%). **Abbreviations:** *ev*SCAs, *ex vivo* sexual conversion assays; Hb, hemoglobin; HBB, human hemoglobin beta; IQR, Interquartile range; LM, Light microscopy; Par, Parasites; RDT, Rapid Diagnostic test; SD, Standard Deviation; SCRs, Sexual conversion rates.
