# Supplementary material for: Elevated Plasmodium falciparum sexual conversion in HbAC and HbAS red blood cells in naturally infected malaria patients
Source: PLoS Pathog. 2026 Sep 29;22(9):e1014542. doi: 10.1371/journal.ppat.1014542 (PMC13623122; doi:10.1371/journal.ppat.1014542)
Supplement: Supplementary file 12 [file ppat.1014542.s014.pdf]

S3 Table. Median SCRs (IQR) by HBB genotype measured in *iv*SCAs with flow cytometry.

	SCRs (%), median (IQR)			
	+Choline		-Choline	
**HBB genotype**	**25-30 hpi**	**35-40 hpi**	**25-30 hpi**	**35-40 hpi**
**HbAA CTRL**	7.78 (2.59)	8.12 (1.35)	22.70 (1.71)	33.10 (3.15)
**HbAA**	7.58 (0.98)	10.80 (0.49)	24.00 (5.36)	35.50 (7.25)
**HbAC**	9.41 (5.14)	13.30 (4.71)	28.60 (7.80)	33.10 (10.9)
**HbAS**	15.00 (6.48)	16.50 (5.64)	29.10 (4.95)	31.70 (2.32)
**HbSS**	8.44 (3.76)	6.96 (3.17)	16.00 (7.53)	18.40 (5.88)
**HbSC**	7.18 (1.81)	7.99 (1.83)	10.20 (1.83)	11.90 (2.81)

Timepoints measured: at 25-30 hpi and 35-40 hpi (Generation 1). **Abbreviations:** CTRL, control; Hb, hemoglobin; HBB, human hemoglobin beta; hpi, hours post-invasion; IQR, inter-quartile range; *iv*SCAs, *in vitro* sexual conversion assays.
