# Supplementary material for: Elevated Plasmodium falciparum sexual conversion in HbAC and HbAS red blood cells in naturally infected malaria patients
Source: PLoS Pathog. 2026 Sep 29;22(9):e1014542. doi: 10.1371/journal.ppat.1014542 (PMC13623122; doi:10.1371/journal.ppat.1014542)
Supplement: Supplementary file 13 [file ppat.1014542.s015.pdf]

S4 Table. Risk analysis of factors across HBB genotype groups in *iv*SCAs.

	HbAA	HbAC	HbAS	HbSS	HbSC	*p*-value$
***n*(%)**	5 (100)	5 (100)	4 (100)	12 (100)	2 (100)	NA
**Blood group *n*(%)**						0.06
O+	1 (20)	2 (40)	2 (50)	7 (58)	1 (50)	
A+	0 (0)	0 (0)	0 (0)	4 (33)	0 (0)	
B+	0 (0)	2 (40)	1 (25)	0 (0)	0 (0)	
A-	2 (40)	0 (0)	0 (0)	1 (9)	0 (0)	
B-	2 (40)	1 (20)	1 (25)	0 (0)	1 (50)	
**Reticulocytes, median (IQR)**	1.42 (0.29)	1.21 (0.73)	1.72 (0.65)	4.55 (2.66)	3.78 (1.93)	<0.001
**Age, median (IQR)**	8 (19)	5 (1)	48.50 (17.50)	27 (7.75)	17.50 (12.50)	<0.05
**Gender *n*(%)**						0.05
Female	4 (80)	3 (60)	4 (100)	3 (25)	1 (50)	
Male	1 (20)	2 (40)	0 (0)	9 (75)	1 (50)	
**Village *n*(%)**						<0.001
Nanoro	5(100)	4(80)	3(75)	0 (0)	1(50)	
Nazoanga	0 (0)	1(20)	1(25)	0 (0)	0 (0)	
Soum	0 (0)	0 (0)	0 (0)	0 (0)	0 (0)	
Séguédin	0 (0)	0 (0)	0 (0)	0 (0)	0 (0)	
Other°	0 (0)	0 (0)	0 (0)	12(100)	1(50)	
**Ethnicity *n*(%)**						<0.001
Mossi	5(45)	4(80)	4(100)	0 (0)	1 (50)	
Gourounsi	0(0)	1(20)	0 (0)	0 (0)	0(0)	
Other°	0 (0)	0 (0)	0 (0)	12 (100)	1 (50)	

$Categorical variables test: Fisher's exact test, Continuous variables test: Kruskal Wallis test. °Other country of origin than Burkina Faso or other ethnicity outside Burkina Faso (not specified) *i.e.,* samples from the UZA. Data from *P. falciparum* non-infected samples collected in Nanoro, Burkina Faso and the UZA (Belgium). Frequencies within each category group are expressed as percentages (%). **Abbreviations:** Hb, hemoglobin; HBB, human hemoglobin beta; hpi, hours post-invasion; IQR, Interquartile range; *iv*SCAs, *in vitro* sexual conversion assays; SCR, Sexual conversion rates; UZA, Antwerp University Hospital.
