# Supplementary material for: Elevated Plasmodium falciparum sexual conversion in HbAC and HbAS red blood cells in naturally infected malaria patients
Source: PLoS Pathog. 2026 Sep 29;22(9):e1014542. doi: 10.1371/journal.ppat.1014542 (PMC13623122; doi:10.1371/journal.ppat.1014542)
Supplement: Supplementary file 14 [file ppat.1014542.s016.pdf]

S5 Table. RBC counts per µl analyzed during flow cytometry to exclude RBC loss of fragile mutant RBCs.

HBB genotype	RBCs/µl (Median[IQR])		
	35-40 hpi (Gen0)	25-30 hpi (Gen1)	35-40 hpi (Gen1)
**HbAA**	9901[8,884]	10,309[753]	9,009[11,102]
**HbAC**	13,699[998]	14,493[12,425]	17,857[19,491]
**HbAS**	17,857[2,660]	16,621[3,622]	17,908[6,241]
**HbSS**	13,246[3,619]	13,524[6,229]	16,270[10,272]
**HbSC**	14,925[0]	10,736[4,889]	17,801[5,454]
p	0.44 (ns)	0.67 (ns)	0.57 (ns)

Measurements were done at three timepoints: 35-40 hpi (Generation 0), and 25-30 hpi and 35-40 hpi (Generation1), for HbAC; HbAS, HbSS and HbSC RBCs. Statistical test: Kruskal-Wallis. Non-significant (ns) p-values are indicated in the table ($p > 0.05$). **Abbreviations:** Gen, generation; Hb, hemoglobin; HBB, human hemoglobin beta; hpi, hours post-invasion; RBCs, red blood cells.
