# Supplementary material for: Elevated Plasmodium falciparum sexual conversion in HbAC and HbAS red blood cells in naturally infected malaria patients
Source: PLoS Pathog. 2026 Sep 29;22(9):e1014542. doi: 10.1371/journal.ppat.1014542 (PMC13623122; doi:10.1371/journal.ppat.1014542)
Supplement: Supplementary file 15 [file ppat.1014542.s017.pdf]

S6 Table. Analysis of parasite stage development by individual parasite culture within each HBB genotype group.

Sample No	Sample type	HBB Genotype	PMR	Late stages (%) 35-40 hpi (Generation 0)	Late stages (%) 25-30 hpi (Generation 1)	Late stages (%) 35-40 hpi (Generation 1)	Fold change 1*	Fold change 2 £	PARASITE DEVELOPMENT PATTERN	SCR (%) 35-40 hpi (Generation 1)
1	HbAA CTRL	HbAA	3	60.00	10.05	33.57	0.2	3.3	EXPECTED DEVELOPMENT	8.81
2	HbAA CTRL	HbAA	3	46.74	7.69	25.27	0.2	3.3	EXPECTED DEVELOPMENT	7.27
3	HbAA CTRL	HbAA	5	11.32	2.02	19.81	0.2	9.8	EXPECTED DEVELOPMENT	9.04
4	HbAA CTRL	HbAA	4	50.00	5.09	12.98	0.1	2.6	EXPECTED DEVELOPMENT	8.51
5	HbAA CTRL	HbAA	5	42.86	5.54	14.36	0.1	2.6	EXPECTED DEVELOPMENT	7.72
6	HbAA CTRL	HbAA	4	40.91	5.07	12.70	0.1	2.5	EXPECTED DEVELOPMENT	6.82
7	HbAA	HbAA	3	52.81	6.79	34.10	0.1	5.0	EXPECTED DEVELOPMENT	10.93
8	HbAA	HbAA	3	21.99	1.68	35.68	0.1	21.2	EXPECTED DEVELOPMENT	10.78
9	HbAA	HbAA	1	45.16	1.66	18.73	0.04	11.3	EXPECTED DEVELOPMENT	9.34
10	HbAA	HbAA	2	70.41	5.29	24.41	0.1	4.6	EXPECTED DEVELOPMENT	10.44
11	HbAA	HbAA	5	8.22	0.00	20.92	0.0	NA	EXPECTED DEVELOPMENT	14.19
12	HbAC	HbAC	2	61.66	12.44	28.33	0.2	2.3	DELAYED DEVELOPMENT	11.31
13	HbAC	HbAC	3	51.43	4.29	31.83	0.1	7.4	EXPECTED DEVELOPMENT	10.42
14	HbAC	HbAC	1	60.67	3.79	8.72	0.1	2.3	DELAYED DEVELOPMENT	16.02
15	HbAC	HbAC	3	7.08	2.45	13.95	0.3	5.7	DELAYED DEVELOPMENT	13.32
16	HbAC	HbAC	2	57.69	4.58	27.49	0.1	6.0	EXPECTED DEVELOPMENT	17.77
17	HbAS	HbAS	3	40.16	5.22	14.52	0.1	2.8	EXPECTED DEVELOPMENT	26.27
18	HbAS	HbAS	3	32.86	5.91	15.90	0.2	2.7	EXPECTED DEVELOPMENT	18.26
19	HbAS	HbAS	2	18.67	1.70	5.14	0.1	3.0	DELAYED DEVELOPMENT	14.82
20	HbAS	HbAS	2	8.94	1.43	3.27	0.2	2.3	(PARTIAL) ARREST/EXTREMELY DELAYED DEVELOPMENT	14.02
21	HbSS	HbSS	2	28.81	5.60	16.41	0.2	2.9	DELAYED DEVELOPMENT	7.79
22	HbSS	HbSS	2	20.59	4.76	15.26	0.2	3.2	DELAYED DEVELOPMENT	7.16
23	HbSS	HbSS	3	7.58	1.87	11.46	0.2	6.1	DELAYED DEVELOPMENT	5.03
24	HbSS	HbSS	4	9.84	1.81	10.66	0.2	5.9	DELAYED DEVELOPMENT	6.69
25	HbSS	HbSS	4	4.71	3.33	1.99	0.7	0.6	(PARTIAL) ARREST/EXTREMELY DELAYED DEVELOPMENT	6.76
26	HbSS	HbSS	2	2.70	1.61	2.13	0.6	1.3	(PARTIAL) ARREST/EXTREMELY DELAYED DEVELOPMENT	4.92
27	HbSS	HbSS	1	1.54	2.13	3.57	1.4	1.7	(PARTIAL) ARREST/EXTREMELY DELAYED DEVELOPMENT	15.48
28	HbSS	HbSS	2	2.86	1.65	1.28	0.6	0.8	(PARTIAL) ARREST/EXTREMELY DELAYED DEVELOPMENT	4.83
29	HbSS	HbSS	2	20.97	6.06	9.41	0.3	1.6	(PARTIAL) ARREST/EXTREMELY DELAYED DEVELOPMENT	10.79
30	HbSS	HbSS	2	3.16	1.33	0.76	0.4	0.6	(PARTIAL) ARREST/EXTREMELY DELAYED DEVELOPMENT	5.16
31	HbSS	HbSS	3	14.97	4.28	5.71	0.3	1.3	(PARTIAL) ARREST/EXTREMELY DELAYED DEVELOPMENT	9.81
32	HbSS	HbSS	3	19.42	8.05	11.14	0.4	1.4	(PARTIAL) ARREST/EXTREMELY DELAYED DEVELOPMENT	7.57
33	HbSC	HbSC	1	1.77	4.76	4.67	2.7	1.0	(PARTIAL) ARREST/EXTREMELY DELAYED DEVELOPMENT	9.82
34	HbSC	HbSC	2	13.59	2.70	9.25	0.2	3.4	DELAYED DEVELOPMENT	6.16

Sample size *n* per group: HbAA *n* = 5, HbAC *n* = 5, HbAS *n* = 4, HbSS *n* = 12 and HbSC *n* = 2); and RBC samples of donors from a malaria non-endemic area ("HbAA CTRL" *n* = 6). For each parameter (PMR, late stages percentage at 35-40 hpi at Generation 0 and 25-30/35-40 hpi, at Generation 1, and fold change 1 and 2), the median (Q2), interquartile range (Q2-Q3), and full percentile range (Q1-Q4) were calculated in HbAA cultures, which served as the reference. Values from cultures in HbAC, HbAS, HbSS and HbSC genotypes were compared against these distributions and color-coded as follows: green = median value of sample is within the HbAA interquartile range (Q2-Q3) values. An expected decrease median value of sample is within the HbAA Q1 values and an expected increase median value of sample is within the HbAA Q4 values; orange = for an expected decrease median value of sample is within the HbAA Q4 values, and for an expected increase median value of sample is within the HbAA Q1 values, and red = for an expected decrease median value of sample is outside the HbAA Q4 values; for an expected increase median value of sample is outside the HbAA Q1 values. Values in red are outside the expected range (*i.e.,* HbAA full percentile range [Q1-Q4]). Cultures were then classified into three developmental classes according to the number of parameters outside the HbAA reference range: (1) *expected development* (≤ 1 parameter outside), (2) *delayed development* (2-3 parameters outside), and (3) (*partially) arrested/extremely delayed development* (≥ 4 parameters outside). Fold-change 1 was defined as the ratio of late-stage proportions between 35-40 hpi (Generation 0) and 25-30 hpi (Generation 1), with an expected decrease (< 1), whereas fold-change 2 was defined as the ratio between 25-30 hpi and 35-40 hpi (both Generation 1), with an expected increase (> 1). These expected patterns reflect the normal developmental progression in parasites grown in HbAA RBCs. Sexual conversion rates (SCR) at 35-40 hpi (Generation 1) are also shown. **Abbreviations:** CTRL, control sample; Fold change 1, late stages proportion at 25–30 hpi (Generation 1) divided by late stages proportion at 35–40 hpi (Generation 0); Fold change 2, late stages proportion at 35–40 hpi (Generation 1) divided by late stages proportion at 25–30 hpi (Generation 1); Hb, hemoglobin; HBB, human hemoglobin beta; hpi, hours post-invasion; PMR, parasite multiplication rate; SCR, sexual conversion rate.
