# Supplementary material for: Elevated Plasmodium falciparum sexual conversion in HbAC and HbAS red blood cells in naturally infected malaria patients
Source: PLoS Pathog. 2026 Sep 29;22(9):e1014542. doi: 10.1371/journal.ppat.1014542 (PMC13623122; doi:10.1371/journal.ppat.1014542)
Supplement: Supplementary file 16 [file ppat.1014542.s018.pdf]

**S7 Table. Median IgG- and IgM-positive proportions of total, asexual and sexual iRBCs by HBB genotype.**

| | IgG-positive % cells | | | IgM-positive % cells | | |
|---|---|---|---|---|---|---|
| HBB genotype | Total iRBCs | Asexual iRBCs | Sexual iRBCs | Total iRBCs | Asexual iRBCs | Sexual iRBCs |
| HbAA | 16.80 (5.91) | 21.40 (9.34) | 2.37 (0.66) | 2.17 (1.78) | 3.39 (2.87) | 0.06 (0.12) |
| HbAC | 19.10 (8.00) | 25.00 (11.60) | 2.58 (1.40) | 1.50 (2.38) | 2.34 (3.58) | 0.04 (0.28) |
| HbAS | 15.70 (9.89) | 18.30 (13.00) | 2.13 (1.30) | 1.96 (1.29) | 2.96 (2.28) | 0.05 (0.12) |
| HbCC | 20.60 (0.87) | 25.70 (2.31) | 2.95 (0.19) | 2.09 (0.33) | 3.26 (0.63) | 0.04 (0.04) |
| HbSC | 13.40 (10.10) | 14.80 (9.87) | 1.83 (1.02) | 1.27 (1.24) | 2.00 (1.94) | 0.00 (0.00) |

Measurements done by flow cytometry. **Abbreviations:** asexual iRBCs, trophozoites; Hb, hemoglobin; HBB, human hemoglobin beta; IgG and IgM, immunoglobulin G and M; iRBCs, infected red blood cells; sexual iRBCs, stage I gametocytes; total iRBCs, trophozoites and stage I gametocytes.
