# Supplementary material for: Elevated Plasmodium falciparum sexual conversion in HbAC and HbAS red blood cells in naturally infected malaria patients
Source: PLoS Pathog. 2026 Sep 29;22(9):e1014542. doi: 10.1371/journal.ppat.1014542 (PMC13623122; doi:10.1371/journal.ppat.1014542)
Supplement: Supplementary file 17 [file ppat.1014542.s019.pdf]

S8 Table. Demographics of participants in IgG/IgM detection assays across HBB genotype groups.

	HbAA	HbAC	HbAS	HbSS	HbSC	*p*-value[$]
***Pf* infection *n*(%)**						0.80
NO (*Pf*-)	4 (22)	5(28)	5 (28)	1 (50)	2 (40)	
YES (*Pf*+)	14 (78)	13 (72)	13 (72)	1 (50)	3 (60)	
**Parasites per µl (median [IQR])**	1518 (4244)	286 (1292)	496 (1363)	1916 (1916)	1235 (6353)	0.54
**Gametocytes in blood *n*(%)**						0.94
NO	16 (89)	15 (83)	17 (94)	2(100)	5 (100)	
YES	2 (11)	3 (17)	1 (6)	0 (0)	0 (0)	
**Age *n*(%)**						0.62
1 to 4 years	10 (56)	9 (50)	12 (67)	2 (100)	4 (80)	
5 to 19 years	8 (44)	9 (50)	6 (33)	0 (0)	1 (20)	
**Gender *n*(%)**						0.23
Female	9 (50)	8 (44)	12 (67)	0 (0)	1 (20)	
Male	9 (50)	10 (56)	6 (33)	2 (100)	4 (80)	
**Village *n*(%)**						0.98
Nanoro	7 (39)	7 (39)	7 (39)	2 (100)	2 (40)	
Nazoanga	6 (33)	6 (33)	8 (44)	0 (0)	1 (20)	
Soum	2 (11)	2 (11)	1 (6)	0 (0)	1 (20)	
Séguédin	3 (17)	3 (17)	2 (11)	0 (0)	1 (20)	
**Ethnicity *n*(%)**						-
Mossi	18 (100)	18 (100)	18 (100)	2 (100)	5 (100)	

Significant p-values ($p < 0.05$*, $p < 0.01$**, $p < 0.001$***). $Categorical variables test: Fisher's exact test; Continuous variables test: Kruskal Wallis test.

Data was analyzed in *P. falciparum* infected ($n = 44$) and non-infected ($n = 17$) plasma samples collected at the Clinical Research Unit (CRUN) in Nanoro, Burkina Faso within the context of the *InHost* study. The ethnicity of all study participants was "Mossi". Frequencies within each category group are expressed as percentages (%). **Abbreviations:** Hb, hemoglobin; HBB, human hemoglobin beta; IQR, Interquartile range; *Pf, P. falciparum.*
