# Supplementary material for: Elevated Plasmodium falciparum sexual conversion in HbAC and HbAS red blood cells in naturally infected malaria patients
Source: PLoS Pathog. 2026 Sep 29;22(9):e1014542. doi: 10.1371/journal.ppat.1014542 (PMC13623122; doi:10.1371/journal.ppat.1014542)
Supplement: Supplementary file 18 [file ppat.1014542.s020.pdf]

S9 Table. **Overview *Pfsa* genotypes detected in samples used in *ev*SCA grouped by HBB genotype: count, mean *ev*SCR and mean parasitemia (D0).**

Genotype	Pfsa.Genotype	Count_ID	Mean_evSCR	Mean_varATS
Pfsa1				
HbAA	-	14	2.9	12,309.5
**HbAA**	**Total**	**14**	**2.9**	**12,309.5**
HbAC	-	10	5.2	4,879.7
HbAC	na	2	4.8	458.5
**HbAC**	**Total**	**12**	**5.1**	**4,142.8**
HbAS	+	3	19.0	34,469.7
HbAS	-	3	18.7	4,737.0
**HbAS**	**Total**	**6**	**18.9**	**19,603.3**
**Grand Total**		**32**	**6.7**	**10,614.6**
Pfsa2				
HbAA	-	14	2.9	12,309.5
**HbAA**	**Total**	**14**	**2.9**	**12,309.5**
HbAC	-	10	5.2	4,879.7
HbAC	na	2	4.8	458.5
**HbAC**	**Total**	**12**	**5.1**	**4,142.8**
HbAS	-	6	18.9	19,603.3
**HbAS**	**Total**	**6**	**18.9**	**19,603.3**
**Grand Total**		**32**	**6.7**	**10,614.6**
Pfsa3				
HbAA	-	13	2.9	13,251.2
HbAA	na	1	3.0	68.0
**HbAA**	**Total**	**14**	**2.9**	**12,309.5**
HbAC	-	9	4.9	3,328.8
HbAC	na	3	5.8	6,585.0
**HbAC**	**Total**	**12**	**5.1**	**4,142.8**
HbAS	+	2	20.3	48,704.0
HbAS	-	3	18.7	4,737.0
HbAS	na	1	16.5	6,001.0
**HbAS**	**Total**	**6**	**18.9**	**19,603.3**
**Grand Total**		**32**	**6.7**	**10,614.6**
Pfsa4				
HbAA	-	12	2.9	5,596.7
HbAA	+	1	3.8	50,130.0
HbAA	-/+	1	1.2	55,043.0
**HbAA**	**Total**	**14**	**2.9**	**12,309.5**
HbAC	-	9	5.3	5,049.7
HbAC	-/+	1	4.0	3,350.0
HbAC	na	2	4.8	458.5
**HbAC**	**Total**	**12**	**5.1**	**4,142.8**
HbAS	-	4	18.2	5,053.0
HbAS	-/+	2	20.3	48,704.0

S9 Table. Overview *Pfsa* genotypes detected in samples used in *ev*SCA grouped by HBB genotype: count, mean *ev*SCR and mean parasitemia (D0).

Genotype	Pfsa.Genotype	Count_ID	Mean_evSCR	Mean_varATS
**HbAS**	**Total**	**6**	**18.9**	**19,603.3**
**Grand Total**		**32**	**6.7**	**10,614.6**
CLAG3.1				
HbAA	-	14	2.9	12,309.5
**HbAA**	**Total**	**14**	**2.9**	**12,309.5**
HbAC	-	10	5.2	4,879.7
HbAC	na	2	4.8	458.5
**HbAC**	**Total**	**12**	**5.1**	**4,142.8**
HbAS	-	2	18.4	4,966.0
HbAS	-/+	3	14.5	6,598.0
HbAS	na	1	33.2	87,894.0
**HbAS**	**Total**	**6**	**18.9**	**19,603.3**
**Grand Total**		**32**	**6.7**	**10,614.6**
FIKK3				
HbAA	-	14	2.9	12,309.5
**HbAA**	**Total**	**14**	**2.9**	**12,309.5**
HbAC	-	10	5.2	4,879.7
HbAC	na	2	4.8	458.5
**HbAC**	**Total**	**12**	**5.1**	**4,142.8**
HbAS	-	3	13.4	5,610.7
HbAS	-/+	3	24.3	33,596.0
**HbAS**	**Total**	**6**	**18.9**	**19,603.3**
**Grand Total**		**32**	**6.7**	**10,614.6**
hyp2				
HbAA	-	2	2.2	4,683.5
HbAA	na	12	3.0	13,580.5
**HbAA**	**Total**	**14**	**2.9**	**12,309.5**
HbAC	-	3	5.3	1,696.3
HbAC	na	9	5.1	4,958.3
**HbAC**	**Total**	**12**	**5.1**	**4,142.8**
HbAS	+	2	24.9	46,947.5
HbAS	-	3	18.7	4,737.0
HbAS	na	1	7.4	9,514.0
**HbAS**	**Total**	**6**	**18.9**	**19,603.3**
**Grand Total**		**32**	**6.7**	**10,614.6**
PTP7				
HbAA	-	11	2.9	13,258.3
HbAA	-/+	2	2.1	10,001.0
HbAA	na	1	3.8	6,490.0
**HbAA**	**Total**	**14**	**2.9**	**12,309.5**
HbAC	-	5	5.0	1,943.8
HbAC	+	1	7.0	10,360.0

S9 Table. Overview *Pfsa* genotypes detected in samples used in *ev*SCA grouped by HBB genotype: count, mean *ev*SCR and mean parasitemia (D0).

Genotype	Pfsa.Genotype	Count_ID	Mean_evSCR	Mean_varATS
HbAC	na	6	4.9	4,939.2
**HbAC**	**Total**	**12**	**5.1**	**4,142.8**
HbAS	-	3	20.0	5,608.3
HbAS	+	1	3.6	6,900.0
HbAS	-/+	1	33.2	87,894.0
HbAS	na	1	16.5	6,001.0
**HbAS**	**Total**	**6**	**18.9**	**19,603.3**
**Grand Total**		**32**	**6.7**	**10,614.6**
REX2				
HbAA	-	7	2.2	11,027.4
HbAA	na	7	3.5	13,591.6
**HbAA**	**Total**	**14**	**2.9**	**12,309.5**
HbAC	-	9	5.6	5,076.9
HbAC	na	3	3.6	1,340.7
**HbAC**	**Total**	**12**	**5.1**	**4,142.8**
HbAS	-	4	22.4	25,526.2
HbAS	na	2	11.9	7,757.5
**HbAS**	**Total**	**6**	**18.9**	**19,603.3**
**Grand Total**		**32**	**6.7**	**10,614.6**

Abbreviations: D0, Day 0; Hb, hemoglobin; HBB, human hemoglobin beta; *ev*SCAs, *ex vivo* sexual conversion assays; *ev*SCR, *ex vivo* sexual conversion rate; SCR, sexual conversion rate; *varATS*, parasite density as detected by PCR targeting *varATS* gene.
