# Supplementary material for: Elevated Plasmodium falciparum sexual conversion in HbAC and HbAS red blood cells in naturally infected malaria patients
Source: PLoS Pathog. 2026 Sep 29;22(9):e1014542. doi: 10.1371/journal.ppat.1014542 (PMC13623122; doi:10.1371/journal.ppat.1014542)
Supplement: Supplementary file 19 [file ppat.1014542.s001.docx]

# S1 Appendix: Assay optimization and validation

## *Ex vivo* sexual conversion assays

To ensure robustness and field feasibility of the *ex vivo* sexual conversion assays (*ev*SCAs) using *Plasmodium falciparum* (*P. falciparum*) isolates with varying parasitemias, we optimized key parameters using mock cultures of the NF54*-gexp02-Tom* transgenic line [1] (Fig 1A). Method optimization focused on: 1) parasite purification and enrichment methods suitable for field conditions, and 2) assessment of the minimal detectable parasitemia and culture volume for accurate SCR quantification.

First, for parasite purification we compared the magnetic activated cell separation (MACS) LD and LS columns (Miltenyi Biotec) using mixed stages of NF54-*gexp02-Tom* parasites (10 ml culture volume, 3% hematocrit [Hct], ~11% parasitemia). In brief, ~255-300 µl of infected-red blood cell (iRBC) pellet was diluted 1:4 with culture medium (final volume: ~1,020-1,200 µl) to reduce viscosity and facilitate efficient separation, and passed through the columns. We performed two elution steps with culture medium: the first elution (6 ml) containing non-infected RBCs and ring stages; and a second elution (6 ml) with the bound fraction after removing the column from the magnet yielding hemozoin-containing stage I gametocytes and schizonts. The first and second elution were then pelleted (1,648 g, 5 min centrifugation). From the purified RBC pellets, 3 µl (LD column) and 1.5 µl (LS column) were used to prepare thin blood smears to quantify the percentage of parasite stages by light microscopy (LM), and to confirm the presence of ring stages (first elution) and gametocytes stage I and schizonts (second elution). Results showed better purification with LD columns, yielding a larger RBC pellet (~8 µl vs. ~1.5 µl) and higher parasite enrichment (~75% vs. ~58%). LD columns were therefore used for all subsequent steps.

Second, to determine the minimal detectable parasitemia, NF54-*gexp02-Tom* parasites were cultured at 3% Hct in 10 and 20 ml volumes to a parasitemia of ~1% iRBC, followed by a 10-fold serial dilution with non-infected RBCs to obtain parasitemias from ~1% to ~0.001%. Then, for each condition (dilution point) RBC pellets were diluted 1:4 with culture medium (end volume of ~1,020 µl) and purified with MACS LD columns. A thin blood smear per condition was prepared from 3 µl of the purified RBC pellet without dilution, followed by IFAs to quantify Pfs16-positive parasites. For fresh (*i.e.,* non-cryopreserved) infected red blood cells (iRBCs), the minimum detectable parasitemia was 0.1% in 10 ml cultures (~3 million parasites) and 0.01% in 20 ml cultures (~600,000 parasites).

Additionally, we compared the minimal detectable parasitemia between matched cultures with fresh and cryopreserved iRBCs at 1% parasitemia (3% Hct, 20 ml culture, and 600 µl RBC pellet at 100 % Hct) to evaluate whether *ev*SCAs could be performed without loss of sensitivity after parasite cryopreservation. Cryopreservation and thawing was done as described in section below *“In vitro sexual conversion assays”: RBC integrity after thawing across human hemoglobin beta (HBB) genotypes”*. In cryopreserved iRBCs, matched to cultures with non-cryopreserved iRBCs (same total and RBC pellet volume, and number of parasites before cryopreservation), the detection threshold increased to 0.1% (~6 million parasites), due to reduced parasitemia after cryopreservation (1 % to ~0.17%). These findings identified 20 ml fresh iRBC cultures as the optimal setup for subsequent assays.

Lastly, to ensure comparable sexual conversion (SC) measurements across smears regardless of parasitemia, we optimized conditions to obtain a homogeneous monolayer of iRBCs mixed with non-iRBCs in the thin blood smears prepared after MACS. We evaluated different dilutions of the MACS-purified iRBC pellet in non-iRBCs and PBS using fresh mock cultures (3% hematocrit, 20 ml) with 10-fold serial parasitemia dilutions (~1% to ~0.01%). The most effective strategy resulted in a two-step dilution: first 1:2 with fresh non-iRBCs, then 1:2 with 1× PBS. The final *ev*SCA protocol was validated on clinical *P. falciparum* samples cultured *ex vivo* (3% Hct, 20 ml fresh culture) at the Clinical research unit in Nanoro, Burkina Faso (CRUN), confirming field feasibility and reliable detection of sexual parasites at parasitemias as low as 0.05% (vs. 0.01% in mock samples). Among 32 successful *ev*SCAs, 41% (13/32) had starting parasitemias of 0.05-0.08%, and the remaining 59% (19/32) ranged from 0.08-1%.

Inter-reader reliability was assessed by re-reading three IFA slides per culture in 12.5% of the total cultures (4/32) by a second reader. Intraclass correlation coefficient (ICC) analysis of the resulting SC rates (SCRs), demonstrated very strong inter-reader agreement with an ICC (2,1) (two-way random effects, single measures, consistency) of 0.96 (95% CI: 0.543-0.998, *p* = 0.0055**). IFA smears showed consistent Pfs16 fluorescence, with a median of 44 stage I gametocytes per smear (IQR: 28-64), an average of 19 stage I gametocytes per 10 fields (confirming at least one visible stage I gametocyte per 10 fields), a median of 1,021 total parasites counted per smear (IQR: 30, with Q1: 1,009 and Q3: 1,039), and 20 counted fields per smear (range: 14-32). Inter-smear reproducibility of SCRs across three IFA replicate slides per culture showed good consistency (ICC of 0.79 [95% CI: 0.66-0.88]; *p* < 0.001). Giemsa-stained replicate smears confirmed a MACS enrichment efficiency of ~70% and that ~54% of parasites observed after MACS were late schizonts [2]. Therefore, if circulating asexual rings had sufficient *ex vivo* culture time to progress to the schizont stage, it can be assumed that circulating sexual rings had sufficient time to progress to Pfs16-expressing stage I gametocytes (note that progression to stage I gametocytes takes a similar time to progression to trophozoites).

To assess whether MACS enrichment could bias SCR measurements by selectively excluding delayed early trophozoite stages in HbAC and HbAS cultures, we examined Giemsa-stained thin blood smears prepared before and after the MACS enrichment from independent *ex vivo* cultures representing HbAA, HbAC and HbAS genotypes.

Parasitemia was quantified in paired before and after MACS samples from each culture. MACS enrichment increased parasitemia as expected, and no evidence was found for differences in enrichment efficiency between HBB genotype groups (Kruskal-Wallis test, *p* = 0.20).

To directly assess potential stage-specific losses during enrichment, parasite stage composition was quantified before and after MACS enrichment. Both early and late trophozoite stages were observed in paired before and after MACS thin blood smears across all HBB genotypes. In addition, trophozoites/stage I gametocytes together with schizonts, represented the majority of parasites before enrichment (83%) and after enrichment (97%), indicating that MACS primarily depleted ring-stage parasites while retaining trophozoite and early gametocyte stages.

Stage distributions were compared between HBB genotype groups using Kruskal-Wallis tests. No significant differences were observed for any parasite stage in thin blood smears of either before or after MACS enrichment (rings: *p* = 0.66 and *p* = 0.86; trophozoites/stage I gametocytes: *p* = 0.12 and *p* = 0.17; schizonts: *p* = 0.17 and *p* = 0.12 for before and after MACS enrichment, respectively). Although the number of cultures was limited, these data provide no evidence that delayed parasite development differentially affected parasite stage composition across HBB genotypes or that MACS enrichment selectively excluded delayed early trophozoite stages. Collectively, these findings support that the elevated SCR observed in HbAS cultures is unlikely to be explained by genotype-specific effects of the MACS enrichment procedure.

## *In vitro* sexual conversion assays

### Red blood cell integrity after thawing across HBB genotypes

To validate the experimental set-up of the *in vitro* SC assay (*iv*SCA), we first evaluated RBC integrity and recovery after thawing across a subset of RBC samples with different genotypes (*n* = 1 for each cryopreserved RBC sample from a malaria-endemic area in Nanoro, Burkina Faso (CRUN): hemoglobin AA [HbAA], HbAC, and HbAS, compared to fresh HbAA RBCs of donors from a non-endemic area at the Institute of Tropical medicine in Antwerp, Belgium (ITM). Thawing was performed using a three-step protocol, sequentially adding three volumes of 3.5% NaCl solution (10× the RBC volume each time), followed by RPMI washes and centrifugation (1,648 g, 5 min) as per standard procedure [3]. Since RBCs with mutant HBB genotypes are more fragile and lysis-prone, RBC loss was assessed across HBB genotypes on day 0 to evaluate whether the thawing procedure led to RBC lysis and at days 0, 3 and 7 to assess for how long the RBCs could be used for culture after thawing. The recovered RBC pellet volume (µl) at day 0 (after thawing) was measured using a pipette and the number of RBCs per milliliter was assessed using the hemocytometer [Scepter™ 3.0 Handheld Cell Counter, Millipore® Cat: PHCC30000]) on day 0, 3, and 7 (after thawing). The minimum required volume of recovered RBC pellet at day 0 (after thawing) to initiate culture wells was at least 30 µl after dilution with RPMI to 50% Hct, samples below this threshold were removed (S1 Fig). RBC counts per milliliter at day 0, 3 and 7 were: fresh HbAA RBCs (non-endemic donors), 3.61e+09, 9.65e+09 and 3.35e+09; cryopreserved HbAA RBCs, 1.06e+10, 1.73e+09 and 3.31e+09; cryopreserved HbAC RBCs, 4.62e+09; 5.41e+09 and 2.67e+09; and cryopreserved HbAS RBCs, 7.07e+10, 1.10e+10 and 3.35e+09. RBC counts decreased by day 7 across all HBB genotypes and thawed RBCs at day 0 appeared morphologically distressed by LM right after the thawing procedure; therefore, we used RBCs three days after thawing for the *iv*SCAs.

Finally, as the cryopreserved RBC samples collected at the Clinical Research Unit in Nanoro, Burkina Faso (CRUN) and the Antwerp University Hospital (UZA) were stored in differently (CRUN: at -80°C before transfer to liquid nitrogen at ITM, three months after sample collection; UZA: directly snap-frozen in liquid nitrogen at ITM) we assessed if this difference in storage had an impact on RBC lysis by comparison of the recovered RBC pellet volume (µl) in three donor-matched (non-endemic) HbAA control samples, preserved according to either the CRUN or UZA protocol, or used fresh. No differences were found in the two cryopreserved samples as these yielded 200 µl of recovered RBC pellet (at 100% Hct) after thawing from an input cryopreserved volume of RBCs at 100% Hct of 500µl (40% of RBC pellet recovery after cryopreservation), compared to 1,000 µl of RBCs at 100% Hct obtained in fresh RBCs. Importantly, SCRs were similar across conditions: 8.5% in fresh HbAA RBCs, and 7.7% versus 6.8% in the cryopreserved HbAA RBCs with storage according to CRUN and UZA, respectively. The results suggest that neither cryopreservation nor subsequent storage methods affected RBC integrity after thawing or SCRs in the *iv*SCA. SCRs showed comparable variability within HBB genotypes across experiments, with tightly clustered values in wild-type HbAA controls (median 8.11 and IQR 1.36; S5 Fig).

### Parasite development in red blood cells of different HBB genotypes

Parasite development in RBCs with different HBB genotypes was assessed during the *iv*SCAs using LM and flow cytometry at multiple time points (25-30 hours post-invasion [hpi] and 35-40 hpi) across two asexual replication cycles (Generation 0 and 1; Fig 2A).

LM data showed that in Generation 0 (35-40 hpi, Fig 3A left graph), parasites in wild-type HbAA RBCs reached the expected mean parasite age of ~43 hpi, closely matching the ~44 hours intraerythrocytic cycle, with schizonts representing the predominant stage (87%). In contrast, parasites growing in mutant HBB RBCs showed genotype-specific developmental delay relative to HbAA cultures, with estimated mean parasite ages of ~40 hpi (HbAC), ~33 hpi (HbAS), ~34 hpi (HbSS), and ~28 hpi (HbSC), corresponding to approximate developmental delays of ~3-15 hours. These delays were reflected in parasite stage distributions, with a shift toward earlier stages (increased ring and trophozoite proportions) vs. and reduced schizont proportions), which was most pronounced in HbSC RBCs. Similar developmental delays persisted in Generation 1 (35-40 hpi; Fig 3A right graph), indicating prolonged parasite development across cycles. Even in HbAA cultures, the mean parasite age at 35-40 hpi in Generation 1 was ~38 hpi, with parasite stage proportions shifted toward earlier stages compared with Generation 0 (60% schizonts versus 87%), suggesting a slightly longer intraerythrocytic cycle duration during these experimental conditions. Developmental delays relative to HbAA cultures persisted across mutant HBB genotypes, and were again supported with increased proportions of earlier stages (rings and trophozoites) and reduced schizont proportions.

Flow cytometry analysis of late stage (schizonts) proportions at 35-40 hpi confirmed the LM observations. In Generation 1 at 35-40 hpi, late stage proportions were significantly lower in HbSS compared with HbAA RBCs (beta-binomial GLM, OR[95% CI] = 0.13 [0.03−0.60], *p [Bonferroni]* = 0.0026; S1B Table), whereas HbAS and HbSC RBCs showed non-significant reductions (Beta-binomial GLM; *p* = 1; S1B Table). Same trends were observed in Generation 0 at 35-40 hpi, but were not-significant (Kruskal-Wallis test, overall *p* = 0.05; S1B Table). These findings support the observations of significantly delayed parasite development in mutant HBB RBCs, particularly in HbSS (and HbSC) cultures, which compromise SCR measurements. At 25-30 hpi (Generation 1), parasites in all HBB genotypes were predominantly at early stages (rings/trophozoites), as expected, indicated by low late stage proportions (Fig 3B), with no significant differences between genotypes (Kruskal Wallis test, *p* = 0.13; S1B Table). The progression from predominantly late stages at 35-40 hpi in Generation 0 to predominantly early stages at 25-30 hpi (Generation 1), followed by reappearance of late stages in at 35-40 hpi in Generation 1 indicates that parasites in HbAA, HbAC and most HbAS cultures retained the capacity to progress through the intraerythrocytic cycle (Fig 3B). Parasite multiplication rate (PMR) analysis further supported continued parasite replication across Generation 0 to 1, with median [IQR] PMRs >1 for all genotypes and slightly lower values in HBB mutant HBB RBCs compared with HbAA (HbAA, 3.5 [2.0]; HbAC, 2.0 [1.0]; HbAS, 3.5 [1.5]; HbSS, 4.0 [2.0]; and HbSC, 2.0 [1.0]) (Kruskal-Wallis test, *p* = 0.83).

As late-stage proportions showed substantial variability (high IQRs) within each genotype, we further investigated whether developmental delay or arrest was more pronounced in specific cultures, taking into account that other host factors may vary apart from HBB genotype. We analyzed six flow cytometry-derived parameters to characterize parasite development: (1) the PMR calculated as the parasitemia at 25-30 hpi in Generation 1 divided by the parasitemia at 35-40 hpi Generation 0; (2) the proportion of late-stage parasites at 35-40 hpi in Generation 0; (3) the proportion of late-stage parasites at 25-30 hpi in Generation 1; (4) the proportion of late-stage parasites at 35-40 hpi in Generation 1; (5) fold change 1, calculated as the proportion of late-stage parasites at 25-30 hpi in Generation 1 divided by the proportion of late-stage parasites at 35-40 hpi in Generation 0; and (6) fold change 2, calculated as the proportion of late-stage parasites at 35-40 hpi in Generation 1 divided by the proportion of late-stage parasites at 25-30 hpi in Generation 1 (S6 Table and S7 Fig).

For each of the six parameters, values from individual cultures of HbAC, HbAS, HbSS and HbSC RBCs were compared with the HbAA reference Q1-Q4 range, which was calculated on 11 HbAA cultures, including the controls, and was used as the reference range for each parameter. An individual culture was considered to deviate from the HbAA reference for a given parameter when its value fell outside this Q1-Q4 range. The number of parameters outside the HbAA reference Q1-Q4 range was then counted for each individual culture. Based on this number, cultures were classified into three developmental classes: *expected development* when ≤ 1 parameter outside the HbAA reference range, *delayed development* when 2-3 parameters outside the HbAA reference range; and (*partially) arrested/extremely delayed* development when ≥ 4 parameters outside the HbAA reference range (S6 Table and S7 Fig). The resulting developmental classification for each culture is reported in S6 Table. The complete workflow from flow cytometry measurements to the developmental classification is illustrated in S7 Fig. In HbAA (5/5 [100%]) and HbAA CTRL RBCs (6/6 [100%]), all cultures exhibited the expected developmental pattern. In contrast, the HBB mutant RBCs showed variable developmental patterns: HbAC −40% (2/5) expected, 60% (3/5) delayed; HbAS−50% (2/4) expected, 25% (1/4) delayed, 25% (1/4) (partial) arrest/extremely delayed; HbSS −0% (0/12) expected, 33% (4/2) delayed, 67% (8/12) (partial) arrest/extremely delayed; HbSC −0% (0/2) expected, 50% (1/2) delayed, 50% (1/2) (partial) arrest/extremely delayed (S6 Table).

## IgG and IgM detection by flow cytometry

To establish the assay for IgG and IgM detection by flow cytometry, plasma titration was performed. Plasma samples from individuals living in malaria-endemic area were defined as “endemic plasma”, whereas samples from individuals living in non-endemic area were defined as “non-endemic plasma”. The plasma titration curves showed a clear dose-response dynamic for IgG detection with the “*P. falciparum-*infected hyperimmune endemic plasma pool”, while for IgM detection the measured percentage of positive cells was low even at the lowest plasma dilution. Therefore, four additional plasma samples were titrated for IgM detection (*i.e.,* “naïve non-endemic plasma” and “acute *P. falciparum-infected* non-endemic plasma at D0, D21 and D23”). While the “naïve non-endemic plasma” remained under the threshold of the “hyperimmune *P. falciparum*-infected endemic plasma pool”, the “acute *P. falciparum*-infected non-endemic plasma at D21 and D23” exceeded the values of both the “*P. falciparum*-infected hyperimmune endemic plasma pool” and the “naïve non-endemic plasma” and showed a dose-response dynamic. Due to the generally low proportions of IgM-positive early trophozoite/stage I gametocyte-iRBCs, we chose the optimal plasma dilution with highest percentage of positive cells detected for IgM (1:320), while for IgG detection we used the dilution in the slope (1:640) (S6 Fig).

## References

1.	Portugaliza HP, Llorà-Batlle O, Rosanas-Urgell A, Cortés A. Reporter lines based on the *gexp02* promoter enable early quantification of sexual conversion rates in the malaria parasite *Plasmodium falciparum*. Sci Rep. 2019;9(1):14595. doi: 10.1038/s41598-019-50768-y.

2.	Bancells C, Llora-Batlle O, Poran A, Notzel C, Rovira-Graells N, Elemento O, et al. Revisiting the initial steps of sexual development in the malaria parasite *Plasmodium falciparum*. Nat Microbiol. 2019;4(1):144-54. doi: 10.1038/s41564-018-0291-7.

3.	EVIMalaR. Methods in Malaria Research 2013.
