# Supplementary material for: Elevated Plasmodium falciparum sexual conversion in HbAC and HbAS red blood cells in naturally infected malaria patients
Source: PLoS Pathog. 2026 Sep 29;22(9):e1014542. doi: 10.1371/journal.ppat.1014542 (PMC13623122; doi:10.1371/journal.ppat.1014542)
Supplement: Supplementary file 20 [file ppat.1014542.s002.docx]

# S2 Appendix: Whole genome sequencing analysis of parasite DNA

Sequencing data were processed using the inhouse plasmovar (v0.3.0) pipeline developed in Nextflow (v26.04.3) [1] leveraging the nf-core framework and resources [2]. The pipeline is openly available at <https://github.com/ITMmalaria/plasmovar> [3] and the parameter and configuration files listing all non-default pipeline options are available in this manuscript’s Zenodo repository (DOI: [https://doi.org/10.5281/zenodo.18419143](https://swe01.safelinks.protection.outlook.com/?url=https%3A%2F%2Fdoi.org%2F10.5281%2Fzenodo.18419143&data=05%7C02%7C%7C7ffca5de7c114ac38e5508de5f5b11ff%7C84df9e7fe9f640afb435aaaaaaaaaaaa%7C1%7C0%7C639053041979484038%7CUnknown%7CTWFpbGZsb3d8eyJFbXB0eU1hcGkiOnRydWUsIlYiOiIwLjAuMDAwMCIsIlAiOiJXaW4zMiIsIkFOIjoiTWFpbCIsIldUIjoyfQ%3D%3D%7C0%7C%7C%7C&sdata=Qi3rpcgnoV3Io09wkoKBgpiOiNY9mQqN3Rm3%2F7TX%2FmM%3D&reserved=0)). Briefly, the pipeline integrates read quality control, trimming, host read removal, alignment, variant discovery, and functional annotation steps

First, reads were subject to quality control using FastQC v0.12.1 [4] and FastQ Screen v0.16.0 [5]. Host reads were depleted using deacon v0.13.2 [6] against a human pangenome index consisting of HPRC Year 1 [7], CHM13v2.0 [8] and GrCh38.p14 genomes (built with the default (*k*=31, w=15) parameters), from which the *P. falciparum* 3D7 reference genome (PlasmoDB release 68 [9]) was subtracted. Fastp v1.0.1 [10] was used to trim adapters and filter out low-quality and short reads.

Filtered reads were then aligned to the *P. falciparum* 3D7 reference using bwa-mem v0.7.19 [11] using the `-Y` option to force soft-clipping for supplementary alignments. PCR duplicates were marked with Picard GATK4 v4.6.2.0 [12] and libraries merged using appropriate read groups (libraries / multi-lane runs). Alignment metrics and coverage statistics were computed with mosdepth v0.3.14 [13] and samtools v1.23.1 [14].

Next, GATK4 v4.6.2.0 [12] was used for variant calling. All compatible GATK4 processes were scattered across 10 intervals created by GATK4 IntervalListTools, encompassing the 14 3D7 plus the mitochondrial and apicoplast chromosomes. Base quality scores were recalibrated using GATK4 BaseRecalibrator and ApplyBQSR using the MalariaGEN Pf8 [15] PASS sites as the training set.

Variant calling was performed using the GATK4 HaplotypeCaller [16], generating (per-interval) gVCF files for each sample. Smith-Waterman parameters were selected to prioritize SNPs over INDELs, based on the pipeline used for the MalariaGEN Pf8 dataset [15]. Joint genotyping across the samples was performed using GenomicsDBImport followed by GenotypeGVCFs (using the `--include-non-variant-sites` option) to produce consolidated variants across all samples (per interval).

Variant quality scores were then recalibrated using GATK4 VariantRecalibrator and ApplyVQSR [12], separately for SNPs and INDELs, after gathering the per-interval VCFs. The Gaussian mixture model was trained on the variants in the *P. falciparum* genetic crosses 1.0 dataset ([17]; The *Plasmodium falciparum* Genetic Crosses project, n.d.) (7g8_gb4, hb3_dd2 and 3d7_hb3) as the true sites training resource, and the Pf8 PASS sites were used as a known sites resource (not used for training). The filter threshold was set at 99.5 for both SNPs and indels during ApplyVQSR.

The outputs of the previous tools were summarized using multiQC v1.35 [18]**.** Lastly, genotypes for the loci of interest were extracted from the VCF file using the bcftools v1.23.1 [19].

# References

1.	Di Tommaso P, Chatzou M, Floden EW, Barja PP, Palumbo E, Notredame C. Nextflow enables reproducible computational workflows. Nat Biotechnol. 2017;35(4):316-9. doi: 10.1038/nbt.3820.

2.	Ewels PA, Peltzer A, Fillinger S, Patel H, Alneberg J, Wilm A, et al. The nf-core framework for community-curated bioinformatics pipelines. Nat Biotechnol. 2020;38(3):276-8. doi: 10.1038/s41587-020-0439-x.

3.	Moris P. ITMmalaria/plasmovar: 0.3.0 (0.3.0). 2026. Zenodo. doi: 10.5281/zenodo.21107324.

4.	Andrews S. FastQC: a quality control tool for high throughput sequence data. 2010.

5.	Wingett SW, Andrews S. FastQ Screen: A tool for multi-genome mapping and quality control. F1000Research. 2018;7:1338. doi: 10.12688/f1000research.15931.2.

6.	Constantinides B, Lees J, Crook DW. Deacon: fast sequence filtering and contaminant depletion. Bioinformatics; 2025.

7.	Liao W-W, Asri M, Ebler J, Doerr D, Haukness M, Hickey G, et al. A draft human pangenome reference. Nature. 2023;617(7960):312-24. doi: 10.1038/s41586-023-05896-x.

8.	Nurk S, Koren S, Rhie A, Rautiainen M, Bzikadze AV, Mikheenko A, et al. The complete sequence of a human genome. Science. 2022;376(6588):44-53. doi: 10.1126/science.abj6987.

9.	Alvarez-Jarreta J, Amos B, Aurrecoechea C, Bah S, Barba M, Barreto A, et al. VEuPathDB: the eukaryotic pathogen, vector and host bioinformatics resource center in 2023. Nucleic Acids Res. 2024;52(D1):D808-16. doi: 10.1093/nar/gkad1003.

10.	Chen S, Zhou Y, Chen Y, Gu J. fastp: an ultra-fast all-in-one FASTQ preprocessor. Bioinformatics. 2018;34(17):i884-90. doi: 10.1093/bioinformatics/bty560.

11.	Li H. Aligning sequence reads, clone sequences and assembly contigs with BWA-MEM. arXiv; 2013.

12.	Van Der Auwera GA, O'Connor BD. Genomics in the Cloud: Using Docker, GATK, and WDL in Terra (1st Edition). 1st ed: O'Reilly Media; 2020 2020.

13.	Pedersen BS, Quinlan AR. Mosdepth: quick coverage calculation for genomes and exomes. Bioinformatics. 2018;34(5):867-8. doi: 10.1093/bioinformatics/btx699.

14.	Li H, Handsaker B, Wysoker A, Fennell T, Ruan J, Homer N, et al. The Sequence Alignment/Map format and SAMtools. Bioinformatics. 2009;25(16):2078-9. doi: 10.1093/bioinformatics/btp352.

15.	MalariaGen. Supplementary data to: Pf8: an open dataset of <i>Plasmodium</i> falciparum genome variation in 33,325 worldwide samples. figshare; 2025.

16.	Poplin R, Ruano-Rubio V, DePristo MA, Fennell TJ, Carneiro MO, Van Der Auwera GA, et al. Scaling accurate genetic variant discovery to tens of thousands of samples. Genomics; 2017.

17.	Miles A, Iqbal Z, Vauterin P, Pearson R, Campino S, Theron M, et al. Genome variation and meiotic recombination in <i>Plasmodium</i> falciparum : insights from deep sequencing of genetic crosses. Genomics; 2015.

18.	Ewels P, Magnusson M, Lundin S, Käller M. MultiQC: summarize analysis results for multiple tools and samples in a single report. Bioinformatics. 2016;32(19):3047-8. doi: 10.1093/bioinformatics/btw354.

19.	Danecek P, Bonfield JK, Liddle J, Marshall J, Ohan V, Pollard MO, et al. Twelve years of SAMtools and BCFtools. GigaScience. 2021;10(2):giab008. doi: 10.1093/gigascience/giab008.
