# Supplementary material for: Elevated Plasmodium falciparum sexual conversion in HbAC and HbAS red blood cells in naturally infected malaria patients
Source: PLoS Pathog. 2026 Sep 29;22(9):e1014542. doi: 10.1371/journal.ppat.1014542 (PMC13623122; doi:10.1371/journal.ppat.1014542)
Supplement: Supplementary file 21 [file ppat.1014542.s021.docx]

| **S10 Table. Key resources and primers/probe sequences.** | | | | |
| --- | --- | --- | --- | --- |
| **Reagent type (species) or resource** | **Designation** | **Source or reference** | **Identifiers** | **Additional information** |
| **Gene**  **(*P. falciparum*)** | *pfap2-g* | PlasmoDB | PF3D7_1222600 |  |
|  | *gexp02* | PlasmoDB | PF3D7_1102500 |  |
| **Gene [Homo sapiens(human)]** | HBB hemoglobin subunit beta | NLM-NCBI | [NCBI_Gene:3043](https://www.ncbi.nlm.nih.gov/gene/3043) | HBBseq |
| **Cell line**  **(*P. falciparum*)** | NF54-*gexp02*-Tom | PMID:[31601834](https://pubmed.ncbi.nlm.nih.gov/31601834/) |  | Maintained in culture with 2 mM choline |
| **Commercial**  **assay or kit** | DNA extraction kit: QIAamp DNA Mini Kit | Qiagen | Cat. No. 51306 |  |
| **Chemical**  **compound,**  **drug** | ML10 | PMID:[28874661](https://pubmed.ncbi.nlm.nih.gov/28874661/)  S. Osborne (LifeArc) and D. Baker  (LSHTM) |  | cGMP-dependent  protein kinase  inhibitor |
|  | Choline  chloride | Sigma-  Aldrich | Cat. No. C7527 |  |
| **Software, algorithm** | BioEdit 7.2., Biological sequence alignment editor | (Informer Technologies) | RRID:  [SCR_007361](https://bioedit.software.informer.com/) | HBBseq |
|  | FlowLogic™ Flow Cytometry Analysis Software version 8.7. | Inivai Technologies™ | [Inivai Technologies™](https://flowlogic.software/) | Flow cytometry analysis software |
|  | Rstudio 2024.12.1 | © 2025 Posit Software, PBC formerly RStudio, PBC | RRID:[SCR_000432](https://posit.co/) | Statistics analysis software |
| **Primers (and probe for *var*ATS qPCR**) | HBB-fwd (5’->3’): GGGTTGGCCAATCTACTCCC  HBB-rev (5’->3’): TCAAGCGTCCCATAGACTCAC |  | RRID:[SCR_003095](http://www.ncbi.nlm.nih.gov/tools/primer-blast/) | HBBseq |
|  | *var*ATS Primer-fw (5’-3’): cccatacacaaccaaytgga  *var*ATS Primer-rev (5’-3’): ttcgcacatatctctatgtctatct  *var*ATS Probe (5’-3’): 6-FAM-trttccataaatggt-NFQ-MGB | Hofmann N,et al., PLoS Med. 2015. [23] |  | *var*ATS qPCR |
| **Antibody** | Pfs16 (mouse, monoclonal) | R.Sauerwein,Teun Bousema, Radboud University | 32F717:B02 | IFA (1:400) |
|  | Goat anti-mouse IgG (H+L) Alexa Fluor 488 | Abcam | ab150113 | IFA (1:1000) |
|  | Alexa Fluor® 488 AffiniPure™ Mouse Anti-Human IgG | Jackson ImmunoResearch Europe | Code: 209-545-098 | Flow Cytometry (1:640) |
|  | PerCP/Cy5.5® Anti-Human IgM antibody (Mouse monoclonal [CH2]) | Abcam | ab201295 | Flow cytometry (1:320) |
|  | APC anti-human CD236 (Glycophorin C) Recombinant Antibody | Biolegend® | Catalog# 378204 / 100 tests |  |
| **Other** | Mounting medium with DAPI, aqueous Fluoroshield | Abcam | ab104139 | IFA |
|  | Hoechst 33342 | BD Pharmingen | Cat. No: 561908 | Flow Cytometry (1:500) |
|  | Agglutination tests:  Seraclone™ Anti-A (ABO1)  Seraclone™ Anti-B (ABO2)  Seraclone™ Anti-D (RH1) Blend | BIO-RAD | REF :  801320  801345  802032 |  |

**Abbreviations:** Hb, hemoglobin; HBB, human hemoglobin beta; IFA, immunofluorescence assay.
