# Supplementary material for: Elevated Plasmodium falciparum sexual conversion in HbAC and HbAS red blood cells in naturally infected malaria patients
Source: PLoS Pathog. 2026 Sep 29;22(9):e1014542. doi: 10.1371/journal.ppat.1014542 (PMC13623122; doi:10.1371/journal.ppat.1014542)
Supplement: Supplementary file 22 [file ppat.1014542.s022.docx]

| **S11 Table. Number of samples (*n*) per *Plasmodium falciparum* infection status and donor HBB genotype at collection.** Information given for the *ex vivo* SC assays *(ev*SCAs), *in vitro* SC assays (*iv*SCAs) and Immunoglobulin (Ig) detection assays. | | | | | | | |
| --- | --- | --- | --- | --- | --- | --- | --- |
|  | **HbAA** | **HbAC** | **HbAS** | **HbSC** | **HbCC** | **HbSS** | **Total** |
| ***Ev*SCAs** | | | | | | | |
| *Pf+* (CRUN, total *n*) | 14 | 12 | 6 | - | - | - | 32 |
| ***IvSCAs*** | | | | | | | |
| *Pf-* (total *n*)  CRUN (*n*)  UZA (*n*) | 5  5  - | 5  5  - | 4  4  - | 2  1  1 | -  -  - | 12  -  12 | 28 |
| ***Ig detection*** | | | | | | | |
| *Pf+* (CRUN)  *Pf-* (CRUN)  Total n (*Pf+* and *Pf-)* | 13  5  18 | 13  5  18 | 13  5  18 | 3  2  5 | 1  1  2 | -  -  - | 43  18  61 |
| Note: *P. falciparum-infected (Pf+)* samples used in the *ev*SCAs must have a parasitemia ≥ 0.05% (*i.e.,* ≥ 2500 parasites/µl), as the established limit of accurate assessment of SC with these assays. **Abbreviations:** CRUN, Clinical Research Unit in Nanoro; Hb, hemoglobin; ITM, Institute of Tropical Medicine in Antwerp; *Pf, P. falciparum*; UZA, Antwerp University Hospital. | | | | | | | |
