# Supplementary material for: Elevated Plasmodium falciparum sexual conversion in HbAC and HbAS red blood cells in naturally infected malaria patients
Source: PLoS Pathog. 2026 Sep 29;22(9):e1014542. doi: 10.1371/journal.ppat.1014542 (PMC13623122; doi:10.1371/journal.ppat.1014542)
Supplement: Supplementary file 23 [file ppat.1014542.s023.docx]

| **S12 Table:** **Overview of experiments and number of cryopreserved RBC samples by HBB genotype in *iv*SCAs.** | | | | | |
| --- | --- | --- | --- | --- | --- |
|  | **Experiments** | | | | |
| **Sample by HBB genotype** | **1** | **2** | **3** | **4** | **Total *n*** |
| **HbAA (CRUN)** | - | 1 | 3 | 1 | 5 |
| **HbAC (CRUN)** | 1 | 2 | 1 | 1 | 5 |
| **HbAS (CRUN)** | 2 | 1 | 1 | - | 4 |
| **HbSS (UZA)** | - | 2 | 2 | 8 | 12 |
| **HbSC (CRUN/UZA)** | - | 2 | - | - | 2 |
| **Total *n*** | **3** | **8** | **7** | **10** | **28** |
| **Abbreviations:** Hb, hemoglobin; HBB, human hemoglobin beta; *iv*SCAs, *in vitro* sexual conversion assays; n, sample size; RBC, red blood cells. | | | | | |
